# Optimizing Exosome Lipid Hybrid Nanoparticles for Enhanced siRNA Delivery and Improved Therapeutic Anticancer Efficacy In Vivo

**DOI:** 10.1021/acsnano.5c16991

**Published:** 2025-12-11

**Authors:** Hend Mohamed Abdel-Bar, Steven Tandiono, Revadee Liam-Or, Calvin C. L. Cheung, Osama W. M. Hassuneh, Qingyang Lyu, Yue Qin, Shunping Han, Nadia Rouatbi, Julie Tzu-Wen Wang, Adam A. Walters, Khuloud T. Al-Jamal

**Affiliations:** † Institute of Pharmaceutical Science, 4616 King’s College London, Franklin-Wilkins Building, 150 Stamford Street, London SE1 9NH, U.K.; ‡ Department of Pharmaceutics, Faculty of Pharmacy, 392053 University of Sadat City, P.O. Box: 32958, Sadat City 32897, Egypt; § Department of Pharmacology and Pharmacy, Li Ka Shing Faculty of Medicine, 71020 The University of Hong Kong, Hong Kong, Hong Kong Special Administrative Region 999077, China

**Keywords:** extracellular vesicles, lipid nanoparticles, DoE, triple negative breast cancer, CD24, CD44, CD47

## Abstract

Exosome lipid hybrid nanoparticles (ELNs) have emerged as promising drug delivery vehicles, integrating the innate targeting capabilities of exosomes with efficient cytosolic delivery of lipid nanoparticles. However, despite growing interest, the development of ELNs for nucleic acid delivery remains a formidable challenge, compounded by diverse production methods and a lack of systematic approaches to optimize their formulation and performance. This study employed a Box-Behnken design and two fabrication methods: freeze–thaw and sonication, to optimize the formulation of ELNs derived from exosomes of five distinct cancer cells. Formulation criteria focused on maximizing the fusion efficiency while minimizing particle size. The impact of the fusion method on cellular association and gene silencing of promising therapeutic targets, CD24, CD44, and CD47, was evaluated. The optimized formulations were subsequently assessed for therapeutic efficacy in 4T1 and B16F10 tumor models. Through careful manipulation of formulation variables, we obtained optimal ELNs with fusion efficiencies exceeding 50% and particle sizes under 170 nm while preserving exosomal markers CD9, CD63, and CD81. Cellular association studies revealed that ELNs specifically targeted their parental cell line, achieving ∼2.5-fold higher siRNA association compared to LNPs. Furthermore, the optimized ELNs facilitated the delivery of therapeutic siRNAs, resulting in robust gene silencing and consequently improved the in vitro macrophage-mediated phagocytosis of treated cancer cells. In vivo studies using 4T1 and B16F10 tumor models highlighted the enhanced therapeutic potential of the optimized ELNs, as evidenced by significant tumor targeting and growth inhibition. These findings underscore the importance of systematic formulation and method optimization in advancing ELNs as effective nucleic acid delivery platforms for cancer therapy.

## Graphical abstract

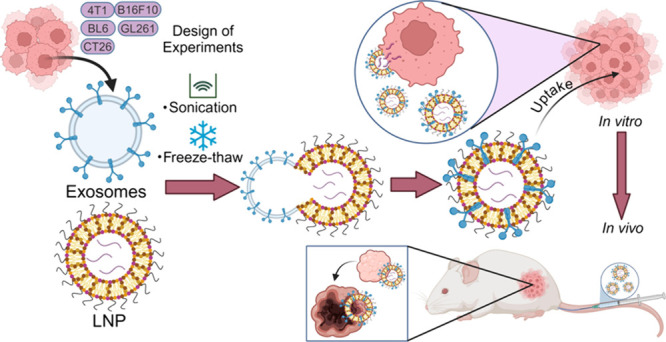

## Introduction

One of the most promising modalities that has yet to achieve clinical application in cancer therapy is small interfering RNA (siRNA). siRNA can selectively knock down oncogenes or pathways contributing to cancer progression, leading to a therapeutic outcome.^1^ However, the efficient delivery of siRNA is restricted by its intrinsic physicochemical properties, including high molecular weight and strong negative charge, which limit passive cellular uptake. Moreover, unmodified siRNAs are prone to rapid degradation in the bloodstream by nucleases, necessitating chemical modification or carrier systems for stabilization and effective delivery.^2^ To overcome these obstacles, researchers have developed various delivery platforms, with nucleic acid-lipid nanoparticles (LNPs) being the most clinically advanced. LNP are based around an ionizable lipid which is neutrally charged at physiological pH, providing biocompatibility, but positively charged at low pH, allowing for formulation with negatively charged nucleic acid and endosomal escape.^3–5^ In addition to the ionizable lipid, cholesterol is included as a stabilizer; it intercalates between other lipids in the LNP, filling voids and ordering the lipid packing to enhance particle stability.^6^ A minor fraction of a PEGylated lipid is included to form a hydrophilic polyethylene glycol coat. This PEG coating provided steric stabilization. It prevents LNP aggregation and shields the particle from opsonization by serum proteins, thereby prolonging circulation time and improving the biocompatibility of the LNP in vivo.^7^ Finally, a helper phospholipid, such as DOPE, is added to facilitate intracellular delivery. DOPE is an unsaturated cone-shaped phospholipid that can adopt an inverted hexagonal phase under acidic conditions, which destabilizes the endosomal membrane and promotes lipid fusion, effectively aiding the escape of the nucleic acid into the cytoplasm.^6^ While LNP are highly effective at delivering siRNA to the cytosol, they are highly liver tropic when delivered systemically and can induce inflammation.^8^ Fusion of LNP with exosomes has been proposed to overcome such shortcomings.

Exosomes are extracellular nanovesicles, ranging in size from 30 to 150 nm, that play a crucial role in cellular signaling and cell-to-cell communication.^9^ Exosomes are composed of a lipid bilayer membrane and contain a diverse cargo of proteins, lipids, and nucleic acids.^10,11^ Exosomes possess several desirable properties for therapeutic applications, such as biocompatibility, stability in circulation, and the ability to cross biological barriers. Moreover, exosomes derived from autologous sources are generally well tolerated and exhibit low immunogenicity compared to synthetic delivery systems, a property that makes them particularly attractive for clinical applications.^12–15^ Their ability to be isolated from a host’s own cells also supports personalized medicine approaches, where exosomes can be tailored to an individual’s specific therapeutic needs. Exosomes, like other nanocarriers, can deliver therapeutic cargos to recipient cells via endogenous uptake mechanisms such as endocytosis.^11^ However, they possess an inherent cellular tropism, determined by parent cell-derived surface molecules such as tetraspanins, integrins, and adhesion proteins, which facilitates precise receptor binding on target cells.^16,17^ This specificity is particularly advantageous in cancer therapy, as it allows exosomes to home and accumulate within the tumor microenvironment, enhancing the localized delivery of therapeutic agents.^17,18^

The application of exosomes has faced challenges in cargo loading, scalability, and manufacturing. Therefore, combining exosomes with synthetic LNP in the form of exosome lipid hybrid (ELNs) could enhance cargo loading efficiency and targeting capabilities while reducing the dependency on large quantities of exosomes.^20^ The integration of exosomes and the LNP leverages the strengths of both platforms and represents a promising approach for advanced therapeutic applications, particularly in the field of precision medicine. ELNs have gained further attention due to their unique characteristics, such as improved stability, extended circulation time, and enhanced cellular uptake.^19–24^ There are ongoing efforts focused on optimizing ELNs' design, improving cargo loading efficiency, enhancing targeting specificity, and maximizing therapeutic efficacy.^22^ The hybridization of exosomes with LNP can be achieved through various strategies, including physical mixing, coextrusion, sonication, or freeze–thaw cycling to form a stable structure.^20,25^ Physical mixing is the simplest method for inducing exosome-LNP fusion, relying on spontaneous membrane interactions under controlled conditions.^23^ However, this simple approach often results in partial fusion, producing a heterogeneous population of hybrid vesicles with inconsistent properties, which can complicate large-scale production and therapeutic application.^20^ Co-extrusion promotes exosome-LNP fusion by repeatedly forcing a mixture of the two vesicle types through polycarbonate membranes with defined pore sizes at controlled temperatures to maintain the fluidity of the lipid bilayers. The mechanical force exerted during this process facilitates the merging of the lipid bilayers, leading to the formation of hybrid vesicles.^25^ Co-extrusion allows for precise control over the final vesicle size, which is crucial for drug delivery applications. However, there is a risk of compromising the integrity of sensitive biological components within the exosomes due to the shear stresses involved.^20^ In the sonication technique, a mixture of exosomes and LNP is subjected to ultrasonic waves. The energy from the ultrasonic waves disrupts the lipid bilayers, allowing them to merge and form hybrid vesicles. Sonication is an efficient method for producing hybrid vesicles with a high degree of membrane fusion. However, excessive sonication can damage the vesicles, leading to the loss of functional components or the generation of lipid debris.^26^ Therefore, the sonication parameters, such as the amplitude and duration of sonication, must be carefully optimized to balance fusion efficiency and vesicle integrity. Freeze–thaw cycling exploits the stress induced by rapid freezing and thawing to promote the fusion of the exosome and liposome membranes. A mixture of exosomes and LNP is subjected to several cycles of freezing at ultralow temperatures followed by rapid thawing at room temperature.^25^ The repeated phase transitions disrupt the bilayers, leading to their eventual fusion. This method is particularly useful for encapsulating sensitive biological molecules as it avoids the use of harsh physical forces. However, the process can be time-consuming, and the degree of fusion may vary depending on the lipid composition and freezing rates used.

We have previously utilized LNP and lipid polymer hybrid systems for the delivery of siRNA targeting CD47.^3,27,28^ CD47 is a transmembrane protein that acts as a “don’t eat me” signal by binding to signal regulatory protein alpha (SIRPα) on macrophages, preventing phagocytosis of cancer cells.^3^ Its overexpression is a common immune evasion mechanism in various malignancies. Preclinical studies have demonstrated that downregulating CD47 can enhance phagocytosis, promote tumor regression, and improve survival outcomes across multiple cancer types, including colorectal, lung, breast, leukemia, and lymphoma.^3,27,29^

While this target has shown promise, as tumorigenesis and metastasis are often multicausal, involving dysregulation of multiple signaling pathways, in addition to CD47, we propose a combinatorial siRNA strategy to simultaneously silence CD24, CD44, and CD47, three distinct but interrelated molecules critical for tumor progression. CD24 is a cell surface glycoprotein that is overexpressed in different cancer cells and contributes significantly to cancer-related mortality. It has been implicated in tumor growth, invasion, and metastasis.^30^ Like CD47, CD24 is also a “don’t eat me signal”, acting as an inhibitor for Siglec-10 on macrophages and other immune cells.^31^ Furthermore, CD24 has been recognized as a marker for cancer stem cells, which are a small cell subpopulation within tumors that are notorious for their ability to self-renew and drive tumor recurrence.^32^ These cells are thought to be responsible for tumor recurrence and resistance to both chemotherapy and radiation therapy. Silencing or inhibiting CD24 expression in cancer cells has shown potential as a therapeutic strategy in cancer treatment with improved outcomes.^33^ Li and co-workers reported an improved therapeutic outcome of attenuated Salmonella carrying siRNA-CD24 interference plasmid and oxaliplatin in a subcutaneous hepatocellular carcinoma model following intratumoral injection.^34^ In addition, knockdown of CD24 expression by developing short hairpin RNA-loaded liposome successfully retarded ovarian tumor growth in nude mice.^35^ The growth of pancreatic cancer was also inhibited through transfection via IV injection of either CD24 monoclonal antibody or lipofectamine short hairpin RNA targeting CD24.^36^ Moreover, silencing of CD24 in lung HARA-B4 cancer cells showed reduced bone metastasis in vitro and in vivo.^37^ Currently, clinical trials are ongoing to assess the efficiency and safety of CD24-targeted therapies, including CD24 antibodies and siRNA-based approaches (siCD24), in cancer patients.^31,38^ Similarly, CD44, a transmembrane receptor for hyaluronic acid, is responsible for tumor proliferation, differentiation, invasion, and motility.^39^ Upon binding to hyaluronic acid, CD44 activates several downstream signaling pathways, including the Ras-MAPK, PI3K-Akt, and Rho family GTPase pathways, which collectively contribute to enhanced tumor growth, metastasis, and chemotherapy resistance.^39,40^ Additionally, CD44 on tumor and stromal cells can enhance the production of immunosuppressive cytokines and the recruitment of regulatory T cells (Treg) and myeloid-derived suppressor cells (MDSCs).^41^ Silencing of CD44 using RNAi has shown promise in preclinical studies and has demonstrated inhibition of tumor growth, suppression of metastasis, and sensitization of cancer cells to chemotherapy.^42^ Systemic administration of self-cross-linkable chitosan-hyaluronic acid dialdehyde nanoparticles improved the antitumor effect of siCD44 in bladder cancer.^43^ Likewise, liposomes loaded with CD44 siRNA successfully inhibited the expression of CD44 on resistant lung cancer cells and improved the sensitivity to cisplatin.^44^

The primary objective of this study was to develop and optimize a novel ELNs system to enhance the delivery and therapeutic efficacy of siRNA. Identifying the lack of systematic approaches within the literature, we employed a structured Design of Experiments (DoE) model to optimize critical process parameters (CPP), including the particle size and fusion efficiency, across exosomes derived from five distinct cancer cell lines. This statistical framework ensured robust, reproducible, and scalable platform fabrication. The optimized ELNs were subsequently evaluated for their ability to codeliver siRNA targeting CD24 (siCD24), CD44 (siCD44), and CD47 (siCD47) in vitro and in vivo models. This study represents the first comprehensive optimization and biological validation of ELNs coloaded with multiple functional siRNAs, offering a translationally viable and mechanistically advanced platform for precision RNAi-based cancer therapy.

## Results and Discussion

### Exosomes Were Successfully Isolated from Cancer Cell Lines

Physicochemical properties of exosomes isolated from five different cancer cell lines are summarized in Table 1. NTA indicated that all isolated exosomes exhibited modal sizes ranging approximately from 100 to 130 nm, with no significant differences observed between the various exosomes. These size measurements are consistent with the reported range for exosomes.^11^ In addition to NTA, the hydrodynamic diameter of the exosomes was measured using DLS, which showed slightly larger size values, ranging from 127.1 ± 3.9 nm (B16F10) to 148.9 ± 5.1 nm (BL6). This increase is consistent with the known tendency of DLS to overestimate particle size due to its sensitivity to light scattering from larger particles and aggregates.^45,46^ The yield of exosomes, expressed as particle concentration, was highest for BL6 (9.5 ± 1.2 × 10^13^ particles/mL) and GL261 (7.2 ± 0.9 × 10^13^ particles/mL) cells, followed by B16F10 (7.1 ± 0.4 × 10^13^ particles/mL) and 4T1 (6.2 ± 0.5 × 10^13^ particles/mL) cells, with the lowest yield observed for CT26 cells (5.1 ± 0.7 × 10^12^ particles/mL). All exosomes exhibited negative surface charges ranging from −7.7 ± 1.2 to −13.3 ± 0.9 mV, consistent with previous findings.^10,11,47^ Total protein concentrations in exosomes varied among the cell lines, with the highest levels observed in 4T1-derived exosomes (397 ± 10.5 μg/mL), followed by B16F10 (350.1 ± 12.8 μg/mL), BL6 (215.1 ± 14.2 μg/mL), GL261 (204.6 ± 10.9 μg/mL), and the lowest in CT26 (98.6 ± 12.5 μg/mL). The particle-to-protein ratio (>5 × 10^10^ particles/μg protein) indicated high exosome purity across all isolates.^11^ The expression of tetraspanins (CD9, CD81, and CD63) on exosome surfaces was confirmed by flow cytometry (Figure S1A–E). MFI was used to quantify the expression of these markers, confirming their enrichment on exosomes, with the highest abundance observed in 4T1 cells and the lowest abundance observed in GL261 cells (Figure 1A). EM images depicted the morphological architecture of isolated exosomes, revealing nearly spherical to subrounded vesicles, nonaggregated vesicular nanostructures (Figure 1B, C). As expected, TEM reports smaller geometric sizes than the hydrodynamic diameters obtained by NTA, consistent with prior observations.^48^

**Table 1:** Characterization of the Isolated Exosomes

	in vitro characterization					
	size (nm)					
exosome source	NTA^a^,^b^ (mode)	DLS^c^	particle concentration (particles/mL)^a^,^b^	zeta potential (mV)^d^,^b^	total protein concentration (μg/mL)^e^,^b^	exosome to protein ratio (particles/μg)^a^,^e^
4T1	119.0 ± 13.5	131.5 ± 5.9	6.2 ± 0.5 × 10^13^	–12.5 ± 0.6	397 ± 10.5	1.5 × 10^11^
B16F10	102.0 ± 18.2	127.1 ± 3.9	7.1 ± 0.4 × 10^13^	–13.3 ± 0.9	350.1 ± 12.8	2.7 × 10^11^
BL6	133.0 ± 12.9	148.9 ± 5.1	9.5 ± 1.2 × 10^13^	–11.0 ± 1.6	215.1 ± 14.2	4.4 × 10^11^
CT26	101.0 ± 17.8	128.9 ± 4.7	5.1 ± 0.7 × 10^12^	–11.5 ± 1.8	98.6 ± 12.5	5.2 × 10^10^
GL261	128.0 ± 12.8	139.3 ± 4.8	7.2 ± 0.9 × 10^13^	–7.7 ± 1.2	204.6 ± 10.9	3.5 × 10^11^

a Values were obtained using nanoparticle tracking analysis (NTA). Exosomes were isolated from 15 mL of supernatants from four T75 tissue culture flasks. Pellets were resuspended in 0.4 mL of PBS prior to NTA measurement.

b Results are expressed as mean ± SD (*n* = 3).

c Measured using the dynamic light scattering technique (DLS).

d Samples were diluted 10 times with deionized water before measurement by Zetasizer Nano ZS at 25 °C.

e Quantified using micro-BCA protein assay.

**Figure 1:**
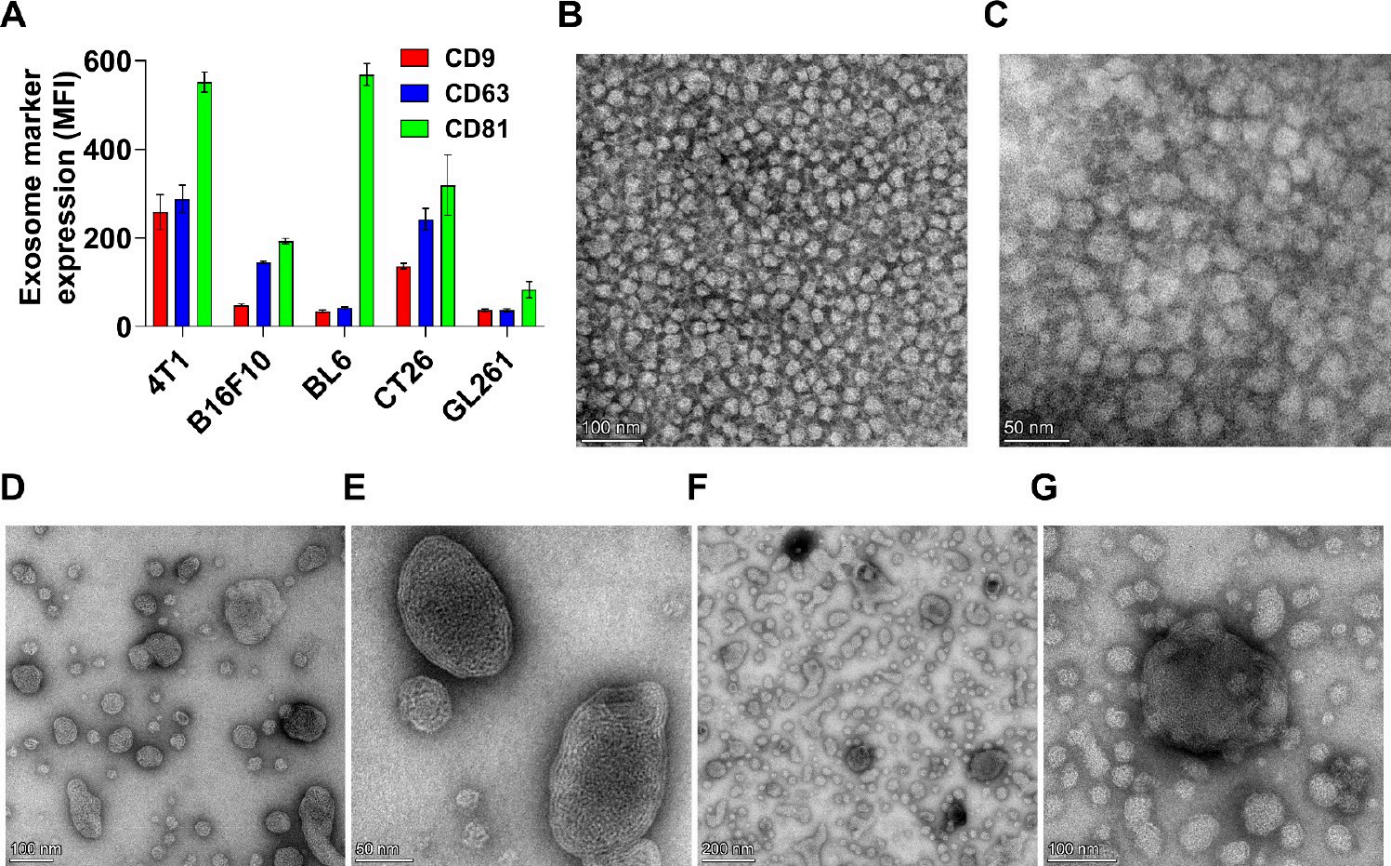
Characterization of the isolated exosomes and the corresponding ELNs. (A) Detection of tetraspanins on the exosome surface by flow cytometry. Exosomes (50 μL, 10 × 10^10^ particles/mL) isolated from 4T1, B16F10, BL6, CT26, and GL261 cells were coupled with aldehyde sulfate latex beads and stained with primary antibodies (anti-CD9, anti-CD63, or anti-CD81, 1:100 v/v) followed by Alexa Fluor 647 Donkey antirabbit IgG secondary antibody (1:100 v/v). Mean fluorescence intensity (MFI) values were used to visualize variations in tetraspanin expression levels among different exosome types, highlighting the predominant abundance of tetraspanins on exosomes derived from 4T1 cells. (B) and (C) Morphological characterization of 4T1-derived exosomes at different magnifications, (D) and (E) the prepared LNP at different magnifications, (F) and (G) 4T1 based ELNs at different magnifications by transmission electron microscopy (TEM), showing nearly spherical nonaggregated nanostructures.

### Exosome-Lipid Nanoparticles (ELNs) Were Optimized via the Box-Behnken Design (BBD)

LNP were prepared comprising four lipids, namely, cholesterol, DOPE, Dlin-MC3-DMA, and C16-PEG2000 (at a molar ratio of 45:15:35:5), using the ethanol injection method. The prepared LNP had a particle size of 118.5 ± 7.5 nm and size distribution expressed as polydispersity index (PDI) of 0.2 ± 0.02, indicating a monodisperse system.^49^ Due to the presence of ionizable cationic lipid, LNP exhibited a partial positive charge of 5.8 ± 0.7 mV and achieved siRNA encapsulation efficiency (EE%) of 93.6 ± 4.8%. LNP exhibited a rounded morphology as revealed in the TEM images, 50–100 nm in diameter, with shapes spanning spherical to mildly elongated and a lamellar rim at the periphery (Figure 1D, E). Exosomes isolated from 4T1, B16F10, BL6, CT26, or GL261 cells were fused with LNP using either the freeze–thaw or sonication method. Previous studies have reported ELNs could be fabricated using different techniques, including fusion, freeze–thaw, sonication, coextrusion, or incubation methods.^22,50^ We selected freeze–thaw and sonication methods, as they represent a compromise between fusion efficiency and preservation of exosomal integrity. During freezing, ice crystals exert pressure on the LNP membrane, causing its disruption and subsequently allowing fusion with exosomes upon thawing.^25,51^ On the other hand, sonication promotes the fusion of exosomes and the prefabricated LNP by generating acoustic cavitation, which leads to the formation and collapse of microbubbles.^51,52^ Freeze–thaw provided a gentle approach suitable for sensitive siRNA payloads, while optimized sonication yielded a high fusion efficiency and uniform particle size, supporting the robustness of our ELNs platform for therapeutic applications.

Expression of exosome markers CD9, CD63, and CD81 was confirmed in ELNs prepared following both hybridization methods (Figure S2). MFI values demonstrated preservation of exosomal markers across both methods and exosome sources (Figure S2E). Consequently, we employed a structured BBD to systematically optimize key Critical Quality Attributes (CQAs), including particle size and fusion efficiency (Tables S1 and S2). These parameters were selected based on their well-established impact on nanocarrier-based siRNA delivery. Particle size governs cellular uptake efficiency as well as systemic circulation, in vivo behavior, and tumor accumulation.^53–56^ Moreover, fusion efficiency determines the extent of lipid-exosome integration, which influences siRNA encapsulation, cellular uptake, and targeting ability.^20,57,58^ Optimizing these CQAs across exosomes from five tumor models enabled robust and scalable fabrication of ELNs suitable for systemic administration. Accordingly, the design generated 17 formulas, and the investigated variables were LNP-Exosome ratio, number of cycles employed, and duration for both fabrication methods across each exosome type (Tables S3–S6). In the freeze–thaw method, each cycle consists of two steps: freezing at −80 °C followed by defrosting at room temperature. Each step duration (either freezing or defrosting) is either 15, 22.5, or 30 min. Each freeze–thaw cycle was repeated 3, 4, or 5 times. For ELNs fabricated by the sonication method, the LNP and exosomes mixture was sonicated at 60 °C for 30, 45, or 60 s, followed by an equivalent cooling duration on ice. Each sonication/cooling cycle was repeated 3, 4, or 5 times. Model validation criteria included selecting models with adjusted and predicted *R* ^2^ values closest to 1, differing by less than 0.2, and minimizing the Predicted Residual Error Sum of Squares (PRESS) after removing the nonsignificant factors. A signal-to-noise ratio, expressed as adequate precision, more than 4, was assigned to ensure the navigation of the model in the design space.^59^ ELNs derived from 4T1, B16F10, BL6, CT26, and GL261 cells are denoted as ELNs_4T1_, ELNs_B16F10_, ELN_BL6_, ELNs_CT26_, or ELNs_GL261_, respectively. The prefix “FT” or “S” was added to indicate preparation method, either freeze–thaw (FT) or sonication (S), e.g., FT- ELN_4T1_.

### Both Exosome and the Critical Process Variables Drive the ELN Fusion Efficiency

Fusion efficiency was quantitatively measured using a FRET assay, where higher FRET dissolution efficiency corresponds to a greater degree of membrane fusion.^60^ FITC exhibits an emission peak at around 520 nm, while DiI emits at approximately 590 nm. Tables S3–S6 indicated that fusion efficiency % for FT-ELNs_4T1_, FT-ELNs_B16F10_, and FT-ELNs_BL6_ was significantly higher than their counterparts prepared using the sonication method, with values ranging from 54.3 ± 3.11 to 97.24 ± 1.25% and from 41.57 ± 3.32 to 96.47 ± 3.32%, respectively. The fusion efficiencies of FT-ELNs_CT26_ and S-ELNs_CT26_ ranged from 35.1 ± 4.6 to 65.1 ± 5.3% and 29.6 ± 2.3 to 75.6 ± 8.9%, respectively.

According to the highest *R* ^2^ values >0.9 and lowest PRESS (Table S7), the quadratic model was suggested as the best fitting model with *p*-values <0.0001 and a nonsignificant lack of fit *p*-values (>0.05). Figures S3 and S4 illustrate the correlation between the experimentally observed and model-predicted values for fusion efficiency and particle size in ELNs prepared using freeze–thaw and sonication techniques, respectively. These parity plots, generated for five different cell line-derived formulations (4T1, B16F10, BL6, CT26, and GL261), demonstrate the predictive accuracy of the BBD models used for formulation optimization. In both techniques, the data points closely align with the identity line (*y* = *x*), indicating a strong linear correlation and minimal deviation between actual and predicted responses. This confirms that the developed models are robust and reliable for guiding the optimization of key formulation parameters affecting the physicochemical properties of ELNs. The influence of the significant variables and interactions is summarized in the model equations listed in Table S8. To quantify the relative influence of each CPP and their interactions on ELNs properties, Pareto charts were generated for all cell lines and fabrication methods (Figures S5 and S6). These charts highlight the statistically significant factors (*p* < 0.05) and their directional effects (positive/negative) on fusion efficiency and particle size. Consistent with earlier reports, which suggested that increasing the exosome concentration improves fusion efficiency,^61,62^ our data revealed that a higher LNP-Exosome ratio negatively impacted fusion efficiency across both freeze–thaw and sonication methods (Figures S7–S11). These findings suggest that excessive lipid content might overwhelm or disrupt the structural balance necessary for optimal fusion, leading to inefficient merging of the LNP and exosome membranes and potentially creating a heterogeneous population of hybrids. Moreover, at higher LNP-Exosome ratios, the available exosomal membrane surface area may be insufficient to effectively interact with or envelop all LNPs, resulting in incomplete fusion and increased heterogeneity. This underscores a critical point that merely increasing the lipid content does not necessarily enhance fusion efficiency and, in fact, may impede it, highlighting the need for a more nuanced approach to ratio optimization. On the contrary, the data obtained confirmed that fusion efficiency is directly proportional to both the number of cycles and the duration, regardless of whether the ELNs were prepared by freeze–thaw or sonication (Figures S7–S11). This is in line with studies by Sato et al., which showed that increasing the number of cycles promotes greater fusion by enhancing opportunities for membrane interaction.^60^ Response contour plots and 3D plots illustrating the significant parameters’ interaction on fusion efficiency for different ELNs prepared by the freeze–thaw technique are presented in Figures 2 and S12. The interaction between the LNP-Exosome ratio and the number of cycles (AB) significantly increased fusion efficiency for ELNs derived from 4T1, B16F10, and GL261 cells prepared using the freeze–thaw method. This suggests that optimizing the balance between the LNP-Exosome ratio, alongside increasing the number of cycles (AB), enhances the fusion process for these cell types. However, the results also show that prolonging the fusion duration can negatively impact efficiency. Specifically, the interaction of the LNP-Exosome ratio and duration (AC), as well as the number of cycles and duration (BC) was detrimental to fusion efficiency in ELNs_4T1_, ELNs_B16F10_, and ELNs_GL261_. This indicates that longer cycle times may be counterproductive for these cell lines, possibly leading to destabilization or degradation of the particles.^58^ In the case of ELNs_BL6_, the only significant factor negatively affecting fusion efficiency was the interaction between the LNP-Exosome ratio and the duration (AC). This suggests that for BL6-derived ELNs, the balance of LNP to exosome must be carefully controlled to prevent reduced fusion efficiency over time. For ELNs_CT26_, the interaction between the number of cycles and duration (BC) was the only parameter found to have a significant negative influence on the fusion efficiency, indicating that increasing the number of fusion cycles over extended durations can be counterproductive for this cell type. In terms of ELNs prepared by the sonication method, the interaction between the LNP-Exosome ratio and duration (AC) negatively influenced both fusion efficiency and particle size across S-ELNs_4T1_, S-ELNs_B16F10_, S-ELNs_BL6_, and S-ELNs_GL261_ (Figure S13). The interaction between the number of cycles and duration (BC) was the only significant interaction that positively increased the fusion efficiency of S-ELNs_CT26_.

**Figure 2:**
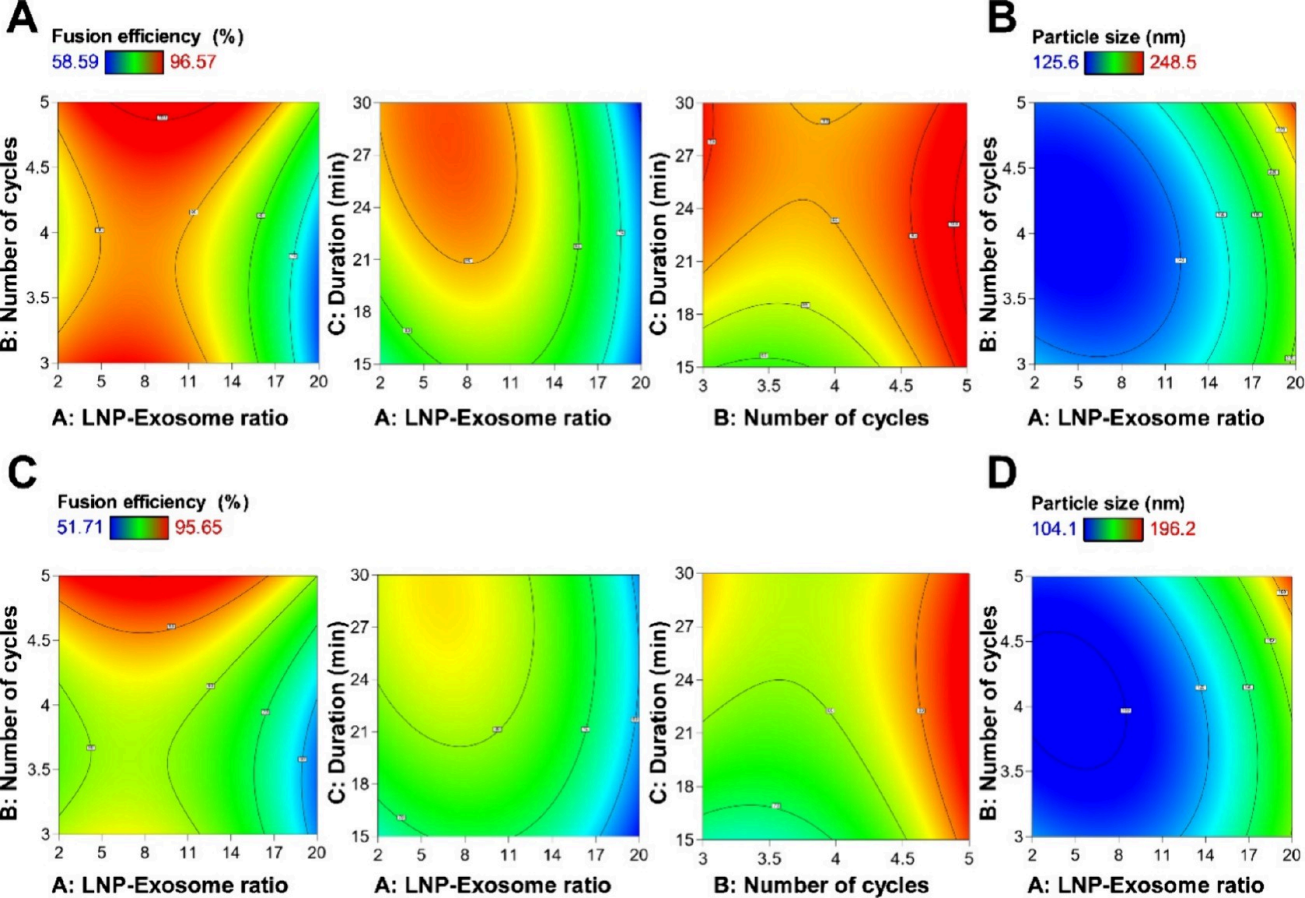
Response contour plots for the significant parameters’ interaction on fusion efficiency and particle size of different ELNs prepared by freeze–thaw technique. (A) FT-ELNs_4T1_ fusion efficiency, (B) FT-ELNs_4T1_ particle size, (C) FT-ELNs_B16F10_ fusion efficiency, and (D) FT-ELNs_B16F10_ particle size. The interaction of the LNP-Exosome ratio and number of cycles (AB) increased the fusion efficiency of FT-ELNs_4T1_ and FT-ELNs_B16F10_. The interaction of the LNP-Exosome ratio and duration (AC) and of number of cycles and duration (BC) had a negative influence on the fusion efficiency of both ELN. The interaction of the LNP-Exosome ratio and the number of cycles (AB) increased the particle size across both ELNs.

### Understanding the Impact of Different Variables on ELNs Particle Size

By inspecting data displayed in Tables S3 and S4 and Figures S7–S11, we noticed that the prepared ELNs prepared by the freeze–thaw method had particle sizes ranging from 125.6 ± 5.9 to 248.5 ± 10.3 nm, 104.1 ± 4.6 to 196.2 ± 13.7 nm, 100.5 ± 8.9 to 185.2 ± 4.9 nm, 127.5 ± 3.2 to 249.6 ± 11.3 nm, and 100.2 ± 8.2 to 186.4 ± 4.5 nm for FT-ELNs_4T1_, FT-ELNs_B16F10_, FT-ELNs_BL6_, FT-ELNs_CT26_ and FT-ELNs_GL261_, respectively. Meanwhile, as shown in Tables S5 and S6 and Figures S7–S11, the respective particle size ranges for S-ELNs_4T1_, S-ELNs_B16F10_, S-ELNs_BL6_, S-ELNs_CT26_, and S-ELNs_GL261_ were 141.3 ± 10.32 to 258.4 ± 6.33 nm, 94.2 ± 10.32 to 172.24 ± 4.65 nm, 120.1 ± 2.32 nm to 219.6 ± 3.65 nm, 133.5 ± 4.65 to 245.6 ± 3.65 nm, and 94.6 ± 4.56 to 176.8 ± 6.36 nm. Equations listed in Table S8 indicate the effect of significant variables on ELNs particle size for each ELNs type prepared by either freeze–thaw or sonication methods. Increasing the LNP-Exosome ratio, number of cycles, and duration significantly increased the particle size of the ELNs fabricated by the freeze–thaw method. Contrarily, the LNP-Exosome ratio had a negative influence on the particle size of all ELNs prepared using the sonication technique. Additionally, the particle size of the ELNs prepared using the sonication method correlated directly with both the number of cycles and duration. While more cycles are beneficial for fusion, they must be controlled to prevent excessive particle growth. As longer cycles allowed for more thorough membrane disruption and mixing, but also increased particle size. These results reinforce the idea that cycle duration must be optimized alongside other variables to achieve the desired nanoparticle characteristics that can affect therapeutic performance.^20^ Earlier studies often optimized these parameters in isolation, which could overlook the complex interplay between variables.^60^ Overall, cell-type-specific responses to these interactions underscore the need for tailored fusion protocols for different ELNs to maximize their therapeutic potential.

These results were used by the Design-Expert software to determine the predictive models. For both freeze–thaw and sonication methods, the quadratic model was the best-fitting model with *p*-values <0.0001 and lack of fit *p*-values >0.05, and *R* ^2^ values exceeding 0.9, indicating strong model fitness. Figures 2, S14 and S15 depict contour plots and 3D plots illustrating how different variable interactions influence the ELNs particle size. For ELNs prepared using the freeze–thaw method, the interaction of the LNP-Exosome ratio and the number of cycles (AB) increased particle size across all preparations. Conversely, the interaction of the number of cycles and duration (BC) had a negative influence on the particle size of FT-ELNs_CT26_ and FT-ELNs_GL261_. FT-ELNs_GL261_ particle size is directly proportional to the interaction of the LNP-Exosome ratio and duration (AC). Data presented in Figure S15 show that the interactions of LNP-Exosome ratio and time (AC), as well as number of cycles and duration (BC), had a negative influence on particle size of S-ELNs_4T1_, S-ELNs_B16F10_, S-ELNs_BL6_, S-ELNs_CT26_, and S-ELNs_GL261_.

The obtained BBD statistical models are valid and can predict the optimum conditions to fabricate ELNs with a maximum fusion efficiency and minimum particle size.

The design space represents a multidimensional combination and interaction of the investigated variables identified through experimental data and verified by BBD to achieve ELNs with maximum fusion efficiency and the smallest particle size. Based on the highest desirability index calculated by the Design Expert (0.808–0.947), one formula for each ELNs was selected and prepared as a checkpoint to validate the models developed by BBD, comparing the predicted response values with the actual observed values. Tables S9 and S10 list the compositions along with predicted and experimental fusion efficiencies and particle sizes. The low percent predicted error confirms that the chosen models effectively manipulate different preparation variables to achieve ELNs with desired physicochemical properties, maximizing fusion efficiency and minimizing particle size as quality target process parameters (QTPP). The particle size of ELNs was further evaluated using NTA complementary to DLS data. As shown in Table 2, NTA confirmed consistent particle sizes across different exosome sources and preparation methods. For freeze–thaw-derived ELNs, the average sizes ranged from 102.5 ± 3.25 nm (GL261) to 112.5 ± 6.36 nm (4T1). Similarly, ELNs prepared by sonication exhibited sizes between 100.1 ± 7.15 nm (GL261) and 135.2 ± 9.24 nm (4T1). These values were slightly lower than the corresponding DLS measurements expressed in Figure S16A–D, as expected, and indicated a relatively homogeneous population with no evidence of large aggregates or multiple populations. All the prepared ELNs showed PDI values less than 0.3, indicating a monodisperse system.^63^ Table 2 shows that all the prepared ELNs exhibited negative surface charge in the range of −3.6 ± 0.4 to −8.62 ± 0.9 mV. All the proposed ELNs prepared by the freeze–thaw method had a significantly higher siRNA EE% compared to those prepared via sonication (*p* < 0.05). As previously noted, LNP exhibited a partial positive charge of 5.8 ± 0.7 mV, significantly higher than the measured ELNs’ surface charge (*p* < 0.0001), indicating successful fusion between exosomes and LNPs. As shown in Figure 1F, G, the optimized 4T1-based ELNs exhibited heterogeneity, combining sub-50 nm spherical vesicles with larger 100–200 nm structures. Some of the larger particles carried ∼20 nm vesicular buds. This ELNs-specific trait, absent in the LNP and exosome controls (Figure 1B–E), supports successful LNP–exosome fusion.

**Table 2:** Characterization of the Optimized Exosome Hybrids Prepared by the Freeze-Thaw and Sonication Method

				particle size (nm)				
exosome source	preparation method	formula code	fusion efficiency (%)^a^,^b^	DLS^c^,^b^	NTA^d^,^b^	PDI^c^,^b^	zeta potential (mV)^e^,^b^	siRNA EE (%)^f^,^b^
4T1	Freeze–thaw^g^	FT-ELNs_4T1_	91.6 ± 4.8	148.0 ± 5.5	112.5 ± 6.36	0.25 ± 0.01	–5.1 ± 0.2	88.6 ± 2.5
	Sonication^h^	S-ELNs_4T1_	68.8 ± 2.1	166.3 ± 3.5	135.2 ± 9.24	0.27 ± 0.02	–4.5 ± 0.7	79.6 ± 3.5
B16F10	Freeze–thaw	FT-ELNs_B16F10_	85.2 ± 5.1	120.8 ± 6.9	105.0 ± 6.11	0.21 ± 0.03	–3.6 ± 0.3	89.5 ± 3.9
	Sonication	S-ELNs_B16F10_	77.0 ± 5.2	114.6 ± 9.5	102.1 ± 8.41	0.15 ± 0.02	–4.3 ± 0.5	80.6 ± 3.6
BL6	Freeze–thaw	FT-ELNs_BL6_	82.1 ± 6.4	119.9 ± 6.7	110.3 ± 5.65	0.19 ± 0.02	–8.6 ± 0.9	86.6 ± 4.5
	Sonication	S-ELNs_BL6_	53.9 ± 1.4	129.3 ± 5.5	115.6 ± 4.78	0.24 ± 0.01	–3.5 ± 0.2	77.3 ± 3.2
CT26	Freeze–thaw	FT-ELNs_CT26_	61.1 ± 5.3	136.9 ± 9.5	112.3 ± 8.65	0.29 ± 0.03	–5.8 ± 0.8	87.6 ± 3.5
	Sonication	S-ELNs_CT26_	73.1 ± 8.2	145.2 ± 6.2	128.3 ± 6.11	0.24 ± 0.04	–8.4 ± 0.4	80.1 ± 2.5
GL261	Freeze–thaw	FT-ELNs_GL261_	87.2 ± 3.2	119.3 ± 6.5	102.5 ± 3.25	0.16 ± 0.02	- 5.2 ± 0.9	88.6 ± 4.5
	Sonication	S-ELNs_GL261_	78.9 ± 2.2	105.6 ± 3.2	100.1 ± 7.15	0.11 ± 0.02	–3.6 ± 0.4	78.6 ± 3.9

a Calculated by measuring fluorescence resonance energy transfer (FRET) dissolution efficiency.

b Expressed as mean ± SD (*n* = 3).

c Measured using the dynamic light scattering technique (DLS).

d Measured by nanoparticle tracking analysis (NTA).

e Measured by electrophoresis.

f Measured by RiboGreen assay.

g Prepared using LNP-Exosome ratio of 2, 4 cycles; each cycle is composed of freezing at −80 °C for 30 min, followed by defrosting at room temperature for 30 min.

h Prepared using LNP-Exosome ratio of 2, 4 cycles; each cycle is composed of sonication at 60 °C for 30 s, followed by incubation on ice for 30 s.

### Cancer Cells Present Varied Levels of Expression of CD24, CD44 and CD47

Flow cytometry analysis was performed to assess the expression levels of the CD24, CD44, and CD47 surface markers in various cancer cell types. As shown in Figures 3A and S17, CD47 was highly expressed across all cell types, while CD44 levels varied, being most abundant in BL6 cells, followed by 4T1, B16F10, CT26, and GL261 cells. CD24 was predominantly expressed in 4T1 cells, with lower levels in BL6 and GL261 cells, and was nearly absent in B16F10 and CT26 cells.

**Figure 3:**
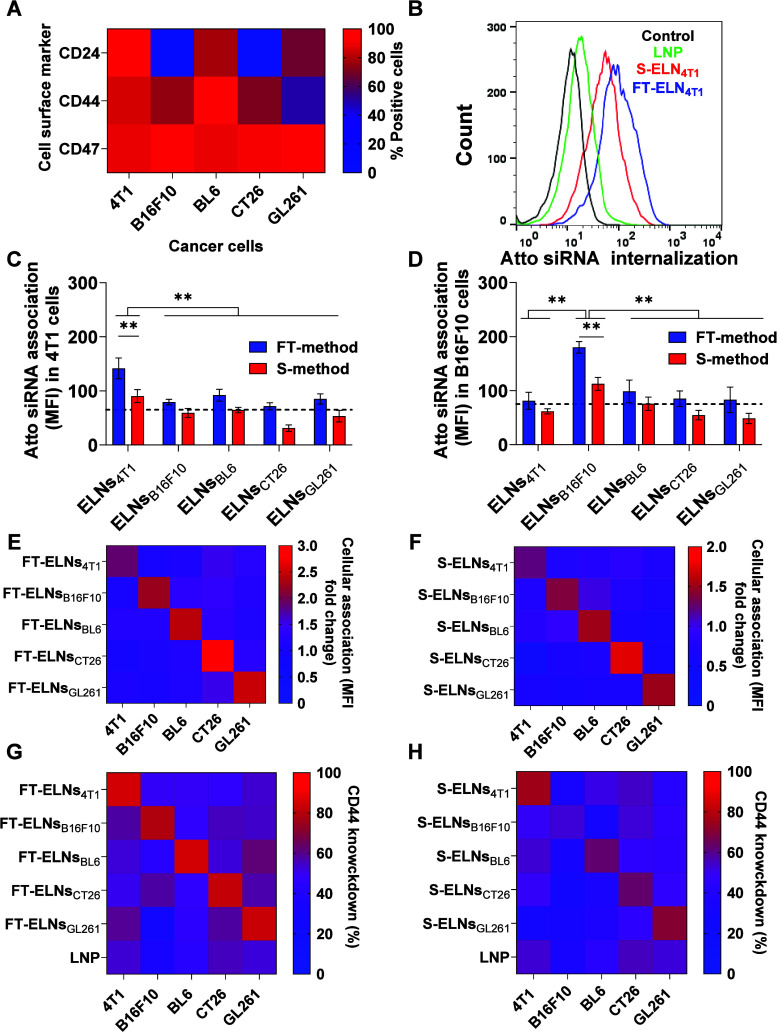
Cancer cell surface markers and in vitrocellular association and gene silencing of the optimized ELNs. Cells (4T1, B16F10, BL6, CT26, and GL261) were stained with antibodies against CD24, CD44, and CD47 prior to flow cytometry analysis. (A) Heatmap illustrating the expression levels of cancer cell markers on various cancer cell types. The heatmap color gradient reflects the magnitude of marker expression, highlighting a high abundance of CD44 and CD47 across all cell types, with minimal CD24 expression observed in B16F10 and CT26 cells. Cells were incubated with the optimized ELNs prepared either by the freeze–thaw or sonication methods loaded with Atto740 siRNA at a concentration of 30 nM for 24 h. (B) Representative flow cytometry histogram of the cellular association of ELNs_4T1_ prepared by freeze–thaw method (blue) or sonication method (red) in comparison with LNP (green) in 4T1 cells. (C, D) Cellular association of different Atto740 siRNA loaded ELNs in 4T1 cells and B16F10 cells expressed as the mean fluorescence intensity (MFI). Dashed line represents the MFI of LNP with respective values of 65.6 ± 5.66 and 75.9 ± 8.17 in 4T1 and B16F10 cells, respectively. Cellular association of ELNs prepared by freeze–thaw method is significantly higher than those prepared by the sonication method (*p* < 0.01). (E, F) Cellular association of Atto740 siRNA loaded ELNs in different cell lines prepared by freeze–thaw and sonication methods, respectively. Cellular association was quantified by the fold increase in MFI compared to LNP and visualized as a gradient on a heatmap. (G, H) CD44 knockdown efficiency by various ELNs in different cell lines, presented as a heatmap. Cells were incubated with either ELNs or LNP at a siCD44 concentration of 30 nM for 48 h. CD44 silencing is presented as percentage reduction of positive cells, with gates drawn based on isotype controls. All ELNs had a higher CD44 silencing in their parent cells compared to LNP. The optimized ELNs prepared by freeze–thaw method showed more efficient gene silencing than those prepared by sonication method. Data points represent mean and SD (*n* = 3). Statistical analysis was performed using one-way ANOVA followed by Tukey’s post-test **p < 0.01. The optimized ELNs achieved a higher degree of siRNA association and gene silencing in the corresponding parent cell compared to LNP.

### FT-ELNs Offer Improved Cellular siRNA Association and In Vitro Gene Silencing

Cellular association of Atto740-siRNA-loaded ELNs was modeled in 4T1, B16F10, BL6, CT6, and GL261 cells. Cell lines were incubated with ELNs prepared by either freeze–thaw or sonication methods or with LNP containing Atto740-siRNA (30 nM) for 24 h. The extent of cellular internalization was expressed as the mean fluorescence intensity (MFI). Representative flow cytometry histograms of cellular association of FT-ELNs_4T1_, S-ELNs_4T1_, and LNP in 4T1 cells are shown in Figure 3B. The MFI values displayed in Figure 3C, D as well as Figure S18 illustrate a significantly higher siRNA association in the form of FT-ELNs compared to S-ELNs (*p* < 0.01). Additionally, the cellular association of LNPs was quantified across all cell lines and showed MFI values of 65.6 ± 5.66 in 4T1, 75.9 ± 8.17 in B16F10, 80.8 ± 10.13 in BL6, 110.5 ± 9.17 in CT26, and 150.9 ± 10.58 in GL261. Therefore, the proposed ELNs prepared by either freeze–thaw or sonication methods exhibited superior siRNA association compared to conventional LNP in the parent cells. The targeting potential of the optimized FT-ELNs or S-ELNs in different cancer cells is illustrated in Figure 3E, F, respectively. Interestingly, all of the proposed ELNs showed preferential association in their parent cells over other cancer cells. Figure S19 shows the in vitro CD44 silencing of the prepared FT-ELNs, S-ELNs, and LNP in different cancer cells at a siRNA dose of 30 nM and 48 h of incubation. Expectedly, the improved cellular association of all FT-ELNs resulted in higher CD44 marker knockdown compared to S-ELNs and LNP, expressed as the remaining %CD44 positive cells (Figure 3G, H). The increased potency of ELNs likely demonstrates their preferential homing potential to their parental cancer cells. The superiority of ELNs in improving cellular association and consequently the gene silencing is in agreement with a previous study utilizing ELNs for CRISPR–Cas9 delivery to mesenchymal stem cells, which neither liposome nor exosome could achieve.^23^ Jhan et al. engineered extracellular vesicles that achieved a 14-fold higher cellular uptake to lung cancer cells and showed an effective GFP-siRNA gene silencing effect comparable to commercial Lipofectamine RNAiMax.^64^ Although all ELNs demonstrated efficient targeting and gene silencing across the different tumor-derived exosome sources, 4T1 and B16F10 cell lines were chosen due to their robust and reproducible response to treatment, and their relevance as widely used and aggressive tumor models in breast cancer and melanoma, respectively. Based on the superior cellular association and gene silencing potential of FT-ELNs over S-ELNs, both FT-ELNs_4T1_ and FT-ELNs_B16F10_ were selected for further studies.

The ability of FT-ELNs_4T1_ to knock down CD24, CD44, and CD47 expression in 4T1 cells was studied using three different concentrations (10, 20, and 30 nM) following 48-h incubation. Figures 4A and S20A,B show representative flow cytometry histograms obtained after incubating 4T1 cells with siCD24, siCD44, and siCD47, respectively, at a dose of 30 nM loaded in either FT-ELNs_4T1_ or LNP. A significant reduction in expression of all markers was obtained following incubation with either FT-ELNs_4T1_ or LNP in a dose-dependent manner (*p* < 0.05). As a consequence of higher cellular internalization of FT-ELNs_4T1_ compared to LNP, a significantly higher knockdown efficiency could be noticed in 4T1 following incubation with the former system (*p* < 0.05). Using FT-ELNs_4T1_, it was possible to reduce CD24, CD44, and CD47 expression by more than 80% with 30 nM siRNA compared to untreated cells (Figure 4B). Similarly, Figure 4C shows that the ability of FT-ELNs_B16F10_ to knock down both CD44 and CD47 was significantly higher than LNP in B16F10 cells in a dose-dependent manner (*p* < 0.05). Representative flow cytometry histograms obtained after incubating B16F10 cells with siCD44 and siCD47 (30 nM) loaded FT-ELNs_B16F10_ and LNP are shown in Figure S20C,D.

**Figure 4:**
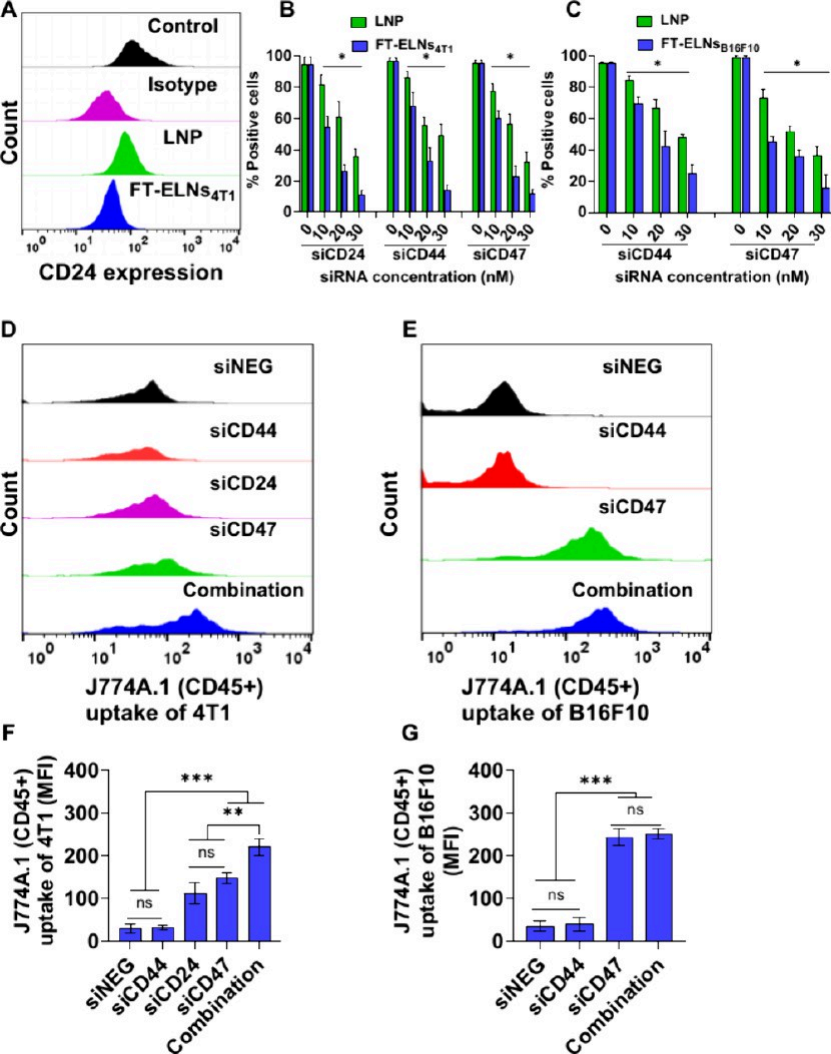
In vitro gene silencing and functional studies of the optimized carrying combinatory siRNA. 4T1 cells were incubated with FT-ELNs_4T1_ containing siCD24, siCD44 and siCD47 at concentrations of 10, 20, and 30 nM each for 48 h. B16F10 cells were incubated with FT-ELNs_B16F10_ loaded with siCD44 and siCD47 at concentrations of 10, 20, and 30 nM each for 48 h. (A) Representative flow cytometry histograms were obtained after incubating 4T1 cells with 30 nM siCD24. (B) Knockdown efficiency of siCD24, siCD44, and siCD47 in 4T1 cells shown as the percentage of positive cells compared to untreated cells. (C) Knockdown efficiency of siCD44 and siCD47 in B16F10 cells shown as the percentage of positive cells compared to untreated cells. The gene silencing of all tested siRNA showed a dose dependent manner (*p < 0.05). The ability to downregulate the “do not eat me” signals CD24 and CD47 to improve macrophage uptake was tested by flow cytometry. Both 4T1 cells and B16F10 cells were stained with CellTrace before being incubated with FT-ELNs_4T1_ and FT-ELNs_B16F10_ for 48 h. 4T1 cells were transfected with 30 nM siCD24, siCD44, siCD47, or a combination, while B16F10 cells were transfected with 30 nM siCD44, siCD47, or a combination. After incubation, cells were collected and cocultured with J774A.1 macrophage cells for 6 h. Cells were then harvested and stained with antimouse CD45 monoclonal before being acquired on a FACs Calibur flow cytometer. Analysis involved gating on CD45 expression followed by quantification of CellTrace fluorescence in the CD45+ population. A representative histogram for macrophage uptake of (D) 4T1 cells, (E) B16F10 cells following gene silencing of the assigned siRNA. (F) Relative MFI of CD45+ J774A.1 cell uptake of 4T1 cells. (G) Relative MFI of CD45+ J774A.1 cells uptake of B16F10 cells. Data represent the average and SD (*n* = 3). Statistical analysis was conducted using one-way ANOVA followed by Tukey’s post-test, ns: nonsignificant, *p < 0.05, **p < 0.01, ***p < 0.001. The optimized FT-ELNs successfully downregulated the target markers and consequently improved macrophage uptake of 4T1 and B16F10 cells.

### Downregulation of “Do Not Eat Me Signal” Improved Macrophage Recognition and Phagocytosis of Cancer Cells

The effect of knocking down CD24 and CD47 as “do not eat me signals” on the macrophage phagocytosis of cancer cells was studied. Both 4T1 cells and B16F10 cells were labeled with CellTrace before being incubated with either siCD24, siCD44, siCD47, or a combination loaded into FT-ELNs_4T1_ or with siCD44, siCD47, or a combination loaded into FT-ELNs_B16F10,_ at a concentration of 30 nM for 48 h, followed by 6 h incubation of treated cells with J774A.1 macrophage cells. FT-ELNs_4T1_ and FT-ELNs_B16F10_ loaded with siNEG were used as the control. Representative histograms for 4T1 cells and B16F10 cells uptake by J774A.1 are displayed in Figure 4D, E. The macrophage uptake represented by MFI is displayed in Figure 4F, G. Downregulation of CD24 or CD47 significantly improved macrophage phagocytosis of 4T1 cells by 3 and 4-fold, respectively, compared to cells transfected with either siNEG or siCD44. Moreover, an enhanced effect could be noticed upon the treatment of 4T1 cells with FT-ELNs_4T1_ loaded with siCD24, siCD44, and siCD47 with ≈2, 1.5, and 6-fold increase compared to siCD24, siCD47, and control groups, respectively. Similarly, knockdown of the CD47 marker using FT-ELNs_B16F10_ on its maternal cells with or without CD44 significantly improved macrophage phagocytosis by 5-fold, compared to B16F10 cells incubated with either siNEG- or siCD44-loaded FT-ELNs_B16F10_. These results suggest the potential consequence of downregulating “do not eat me signals” on macrophage recognition. The limited effect of siCD44 on macrophage uptake is consistent with its biological function. Unlike CD47, which actively suppresses phagocytosis *via* the SIRPα pathway, CD44 is not directly involved in immune evasion signaling. Its primary roles lie in cancer cell adhesion, migration, and stemness, which may explain why its silencing does not enhance macrophage-mediated clearance.

### FT-ELNs Exhibited Better Tumor Deposition Compared to Conventional LNP

The in vivo biodistribution profile of FT-ELNs_4T1_ and FT-ELNs_B16F10_ was assessed in 4T1 tumor-bearing BALB/c mice and B16F10 tumor-bearing C57BL/6 mice, respectively, after systemic administration. As a benchmark, animals were also injected with LNP. Both ELNs and LNP were labeled with DiR dye (1 mol % of total lipid) during formulation to enable fluorescence tracking. Figure 5A, B shows whole-body imaging of mice following a single i.v. injection of either FT-ELNs or LNP. Both ELNs displayed preferential tumor accumulation compared to LNP at all tested time points. Animals were sacrificed 24 h postinjection, and at post-mortem, organs and tumors were imaged, and a significantly higher quantities of FT-ELNs compared to LNP were detected in tumors (*p* < 0.05) in both tumor models (Figure 5C, D). As expected, since both FT-ELNs and LNP contain ionizable lipid, a high signal was detected in the liver. However, FT-ELNs had a significantly higher tumor-to-liver ratio than LNP in both models (*p* < 0.05) (Figure 5E, F).

**Figure 5:**
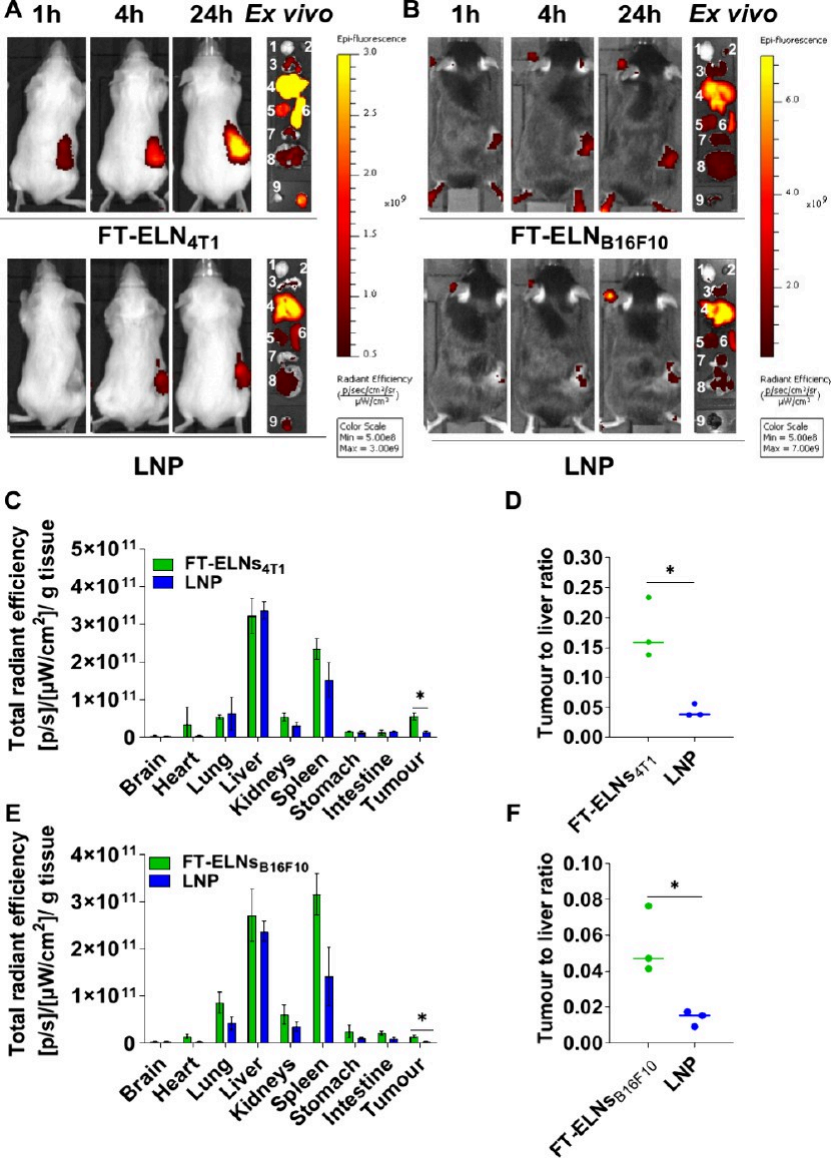
In vivo biodistribution of DiR-labeled FT-ELNs_4T1_and FT-ELNs_B16F10_in 4T1 tumor-bearing BALB/c mice and B16F10 tumor-bearing C57BL/6 mice after systemic administration. BALB/c mice and C57BL/6 mice were inoculated subcutaneously with 1 × 10^6^ 4T1 cells or B16F10 cells respectively in right flank. When the tumor reached ∼70–80 mm^3^, mice were i.v. injected with 100 μL of either DiR labeled (1 mol % of total lipid) ELNs or LNP in HEPES buffer pH 7 containing approximately 1 nmol of siNEG. Animals were imaged using an IVIS imaging system. (A, B) Representative whole-body live (dorsal) imaging. BALB/c and C57BL/6 mice were imaged at 750/780 nm to track DiR-labeled LNP and ELNs_4T1_ or ELNs_B16F10_ at 1, 4, and 24 h postinjection. Animals were culled at 24 h postinjection, and their organs were excised for analysis. (C–F) Ex vivo organ biodistribution profile and tumor to liver ratio of DiR labeled LNP and ELNs_4T1_ or ELNs_B16F10_ in 4T1 tumor-bearing BALB/c mice and B16F10 tumor-bearing C57BL/6 mice normalized to the organ weight. The organs are labeled numerically (1. brain, 2. heart, 3. lung, 4. liver, 5. kidneys, 6. spleen, 7. stomach, 8. intestine and 9. Tumour). Bars represent mean and SD (*n* = 3). Statistical analysis was performed using Student *t* test *p < 0.05, **p < 0.01. ELNs showed higher tumor uptake than LNP.

### Systemic Administration of FT-ELNs Resulted in Reduced Tumor Growth Compared to LNP

The therapeutic outcomes of the optimized FT-ELNs_4T1_ and FT-ELNs_B16F10_ were evaluated in comparison to LNP in the 4T1 and B16F10 animal bearing tumor model, respectively. 4T1 breast tumor bearing BALB/c mice were injected by either PBS, LNP_siNeg_, FT-ELNs_4T1‑siNeg_, LNP, or FT-ELNs_4T1_. The doses of siCD24, siCD44, and siCD47 were 33.3 μg/kg each. B16F10 melanoma-bearing C57BL/6 mice were injected with either PBS, LNP_siNeg_, FT-ELNs_B16F10‑siNeg_, LNP, or FT-ELNs_B16F10_, with doses of siCD44 and siCD47 at 33.3 μg/kg each. A two-dose regimen was administered on days 8 and 14 for BALB/c mice and on days 6 and 9 for C57BL/6 mice. The tumor growth was evaluated in terms of tumor volume. A significant tumor growth retardation following administration of FT-ELNs_4T1_ in comparison to other groups, including PBS, LNP_siNeg_, FT-ELNs_4T1‑siNeg_ LNP (*p* < 0.01) was observed (Figure 6A). Moreover, a significant difference was observed between PBS and LNP (*p* = 0.0361), indicating modest therapeutic activity of siRNA-loaded LNP. Similarly, in the B16F10 model, FT-ELNs_B16F10_ outperformed the other treatments in reducing tumor progression (*p* < 0.01) (Figure 6B). No significant differences were observed between PBS and LNP, LNP_siNEG_, or FT-ELNs_B16F10‑siNEG_, suggesting a more resistant tumor phenotype under the current treatment conditions. These findings support the selective therapeutic benefit of the optimized siRNA-loaded ELNs in aggressive tumor models. This outcome further supports the platform’s translational promise and warrants future exploration under extended dosing schedules and combination therapy strategies.

**Figure 6:**
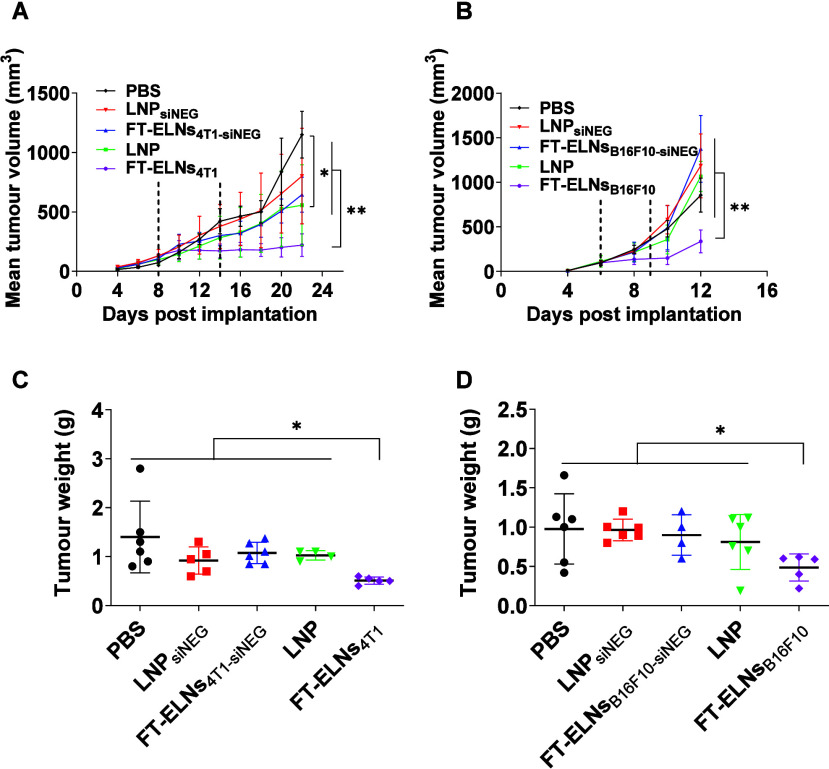
In vivo therapeutic efficacy assessment of FT-ELN in 4T1 and B16F10 tumor models. BALB/c mice and C57BL/6 mice (*n* = 6 per group) were implanted subcutaneously with 1 × 106 4T1 cells or B16F10 cells, respectively. On days 8 and 14 (dashed lines), BALB/c mice were i.v. injected with either PBS, LNPsiNeg, FT-ELNs4T1-siNeg, LNP, or FT-ELNs4T1. The doses of siCD24, siCD44 and siCD47 were 33.3 μg/kg each. On days 6 and 9 (dashed lines), C57BL/6 mice were i.v. injected with either PBS, LNPsiNeg, FT-ELNsB16F10-siNeg, LNP, or FT-ELNsB16F10. The dose of siCD44 and siCD47 was 33.3 μg/kg each. Tumour size was monitored every 2 days. At the end point, mice were euthanized, and individual organ and tumor weights were recorded. (A, B) Monitoring tumor size during treatment courses prior to sacrifice. Both FT-ELNs_4T1_ or FT-ELNs_B16F10_ induced significantly greater tumor suppression compared to all other groups. (C, D) Mice reaching their predefined study end point (Day 25 for 4T1, and day 14 for B16F10) were culled and their tumors weighed. Mice which were culled prior to this end point, or whose tumor had resolved, are not plotted on this graph. Data points represent the mean and SD. Statistical analysis was performed using One way ANOVA followed by Tukey post-test, **p < 0.01, *p < 0.05.

C57BL/6 mice were sacrificed on day 14 following B16F10 tumor inoculation, while BALB/c mice bearing 4T1 tumors were euthanized on day 25 postinoculation, and the excised tumors were imaged (Figure S21A,B). The biocompatibility of the injected platforms was monitored by comparing organ weights between groups. Figure S21C,D illustrate nonsignificant differences in organ weights between groups except for the spleen. Notably, the weight of the spleen in BALB/c received FT-ELNs_4T1_ was significantly less than that of other groups (*p* < 0.01). Splenomegaly is often associated with poor prognosis and systemic inflammation in the 4T1 tumor model. Therefore, the lower spleen weight suggests a more favorable therapeutic response.^65^ Moreover, Figure 6C, D shows a significant reduction in tumor weight could be observed in animals receiving either FT-ELNs_4T1_ or FT-ELNs_B16F10_ (*p* < 0.05).

There have been various iterations of the ELN system developed for siRNA delivery. Most commonly, exosomes are fused to liposomes rather than LNP. For example, liposomes have been fused to extracellular vesicles from SKOV3 cells; these hybrids induced comparable gene silencing to liposomes in some cell models but with improved biocompatibility.^66^ Similarly, Zhou et al. fused HCC-derived exosome membranes with liposomes to create ELNs, achieving enhanced HCC-specific targeting via heparan sulfate-mediated uptake, nonlysosomal trafficking, and 1.7-fold greater gene knockdown. These ELNs induced tumor regression in 33% of mice and extended survival, while maintaining low immunogenicity and strong safety.^67^ In another approach, Zhang et al. employed bovine-milk-derived exosomes fused with cationic liposomes to create “hybridosomes” for oral siRNA delivery. These displayed excellent cytocompatibility in Caco-2 cells, stability in simulated intestinal fluid, and superior intestinal transcytosis. Furthermore, genes were comparable to commercial reagents.^68^

To the best of our knowledge, this is the first study to explore the combinatorial silencing of siCD24, siCD44, and siCD47 by using a nanoparticle-based delivery platform. This triple siRNA therapy was specifically investigated in the 4T1 breast cancer model, which exhibits a high expression of all three immune evasion markers. In the B16F10 melanoma model, we evaluated a dual siRNA combination (siCD47 and siCD44) due to its distinct molecular expression profile. The integration of these siRNAs represents a multifaceted therapeutic strategy, targeting key oncogenic and immune evasion pathways that are critical for tumor cell survival, metastatic potential, and resistance to immune clearance. This approach is designed to mitigate the risk of resistance that often hampers monotherapies. Concurrent silencing of these three genes is anticipated to produce enhanced effects, resulting in more substantial inhibition of tumor growth and metastasis, thereby potentially enhancing therapeutic efficacy. Dual CD24 and CD47 blockade *via* transcription suppressor factor (ZBTB28) or bispecific antibody fusion protein enhanced macrophage phagocytosis of breast cancer, which subsequently improved therapeutic outcomes.^69,70^ A previous study demonstrated that CD44 and CD24 expression silencing via lentivirus-based short hairpin RNA suppressed cancer stem cell stemness and epithelial-mesenchymal transition regulator Twist in nasopharyngeal carcinoma cells in vitro.^71^ An important future direction will be the integration of ELNs with traditional chemotherapeutic agents to achieve combination therapy and the long-term follow-up to establish the survival benefits of such a regimen.

## Conclusions

This study presents a systematic approach for the design and optimization of ELNs using the BBD. Rationally engineered via freeze–thaw fusion, the optimized ELNs were successfully developed to codeliver siRNAs targeting the “don’t eat me” signal CD47, with or without CD24 and CD44. These hybrid systems demonstrated improved fusion efficiency, reduced particle size, enhanced cellular uptake, efficient gene silencing, and potent anticancer activity. In vivo studies in aggressive tumor models (4T1 and B16F10) confirmed the therapeutic efficacy of FT-ELNs_4T1_ and FT-ELNs_B16F10_ in delaying tumor growth without signs of toxicity. These findings underscore the promise of freeze–thaw-engineered ELNs as a robust and translatable platform for RNAi-based cancer therapy. Moreover, future research will focus on investigating combination therapies involving ELNs and chemotherapeutics, which are hypothesized to further enhance therapeutic efficacy in preclinical mouse models. Such efforts may support the integration of ELNs into precision medicine strategies for improved cancer patient care.

## Experimental Section

### Materials

Cholesterol, citric acid, absolute ethanol, deuterium oxide (D_2_O), skim milk, 1,1′-dioctadecyl-3,3,3′,3′-tetramethylindocarbocyanine perchlorate (DiI), 1,1’-dioctadecyl-3,3,3′,3′- tetramethylindotricarbocyanine Iodide (DiR), bovine serum albumin (BSA), and glycine were obtained from Sigma-Aldrich, UK. Phosphotungstic acid negative staining solution (2%; PH1308) was purchased from Phygene, Fujian, China. Carbon-coated copper grids (300 mesh, BZ11023a) were purchased from Zhongjingkeyi Technology, Beijing, China. Aldehyde sulfate latex beads, CellTrace far red, RiboGreen reagent, micro-BCA kits, formaldehyde, glutaraldehyde, sucrose, MEM nonessential amino acids (100×), BSA solution (7.5%), β-mercaptoethanol (55 mM), Accutase, N-2 supplement (100×), and B-27 supplement (50×) were obtained from Thermo Fisher Scientific, UK. Dilinoleylmethyl-4-dimethylaminobutyrate (DLin-MC3-DMA, orb507413) was purchased from Biorbyt, UK. 1,2-dioleoyl-sn-glycero-3-phosphoethanolamine (DOPE) and N-palmitoyl-sphingosine-1–1­[succinyl (methoxy polyethylene glycol) 2000] (C16-PEG2000 Ceramide) were purchased from Avanti Polar Lipids, USA. 1,2-dioleoyl-sn-glycero-3-phosphoethanolamine fluorescein isothiocyanate (DOPE-FITC) was purchased from Nanocs Inc., USA. CD24, CD44, and CD47 siRNA and nontargeting siRNA Control (siNEG) were purchased from Dharmacon, UK. Atto740 siRNA was obtained from Eurogentec, Belgium. Isoflurane (IsoFlo) for anesthesia was purchased from Abbott Laboratories Ltd., UK. RPMI-1640 media, Dulbecco’s modified Eagle medium (DMEM), fetal calf serum (FCS), fetal bovine serum (FBS), penicillin/streptomycin, Trypsin/EDTA, l-glutamine, and phosphate-buffered saline (PBS) were obtained from Gibco, Invitrogen, UK. 4T1 breast cancer cells (CRL-2539), B16F10 melanoma cells (CRL-6475), CT26 colon cancer cells (CRL-2638) and J744A.1 macrophage cells (TIB-67) were purchased from ATCC, UK. GL261 glioblastoma cells were obtained from Caliper Life Sciences, UK. BL6-NPE glioblastoma cells were kindly provided by Professor Steven Pollard from the University of Edinburgh,^72^ UK. Antimouse CD24-PE (clone 30-F1), antimouse CD44-APC (clone IM7), antimouse CD47-APC (clone miap301), antimouse CD45-PE monoclonal antibody (clone 30-F11), rat IgG2a-APC isotype, κ, rat IgG2b-APC, κ isotype, rat IgG2c-PE, κ isotype, and rat IgG2b-PE, κ were purchased from Biolegend, UK. Polyclonal anti-CD9/MRP-1 rabbit antimouse (catalogue number bs-2489R) was purchased from Bioss, USA. Monoclonal rabbit anti-CD63 antimouse (clone number EPR21151), monoclonal rabbit recombinant anti-CD81 antimouse (clone number EPR4244), and donkey antirabbit IgG 1-Alexa Fluor 647 secondary antibody (clone ab150075) were obtained from Abcam, UK.

### Methods

#### Cell Culture

Murine 4T1, B16F10, CT26, GL261 cancer cells, and J774A.1 macrophage cells were cultured in RPMI-1640 media supplemented with 10% v/v FCS, 50 U/mL penicillin, 50 μg/mL streptomycin, and 1% v/v l-glutamine. BL6 cells were grown in DMEM media supplemented with 0.145% glucose, 50 U m/L penicillin, 50 μg/mL streptomycin, 1% MEM nonessential amino acids, 0.5% B27 supplement, 0.5% N2 supplement, 0.012% BSA, and 0.1 mM β-mercaptoethanol. Cells were incubated in 5% CO_2_ at 37 °C. All cells were collected by trypsinization, except BL6 was detached using Accutase. Prior to harvesting conditioned culture media for exosome isolation, cells were cultured in serum-free media for 24 h.

#### Isolation of Exosomes from Conditioned Culture Media

Exosomes were isolated from 4T1, B16F10, BL6, CT26, and GL261 cells using ultracentrifugation onto a sucrose cushion (Figure S22).^11^ Conditioned culture media were collected when cell confluency reached 80–90% and centrifuged twice at 500*g* for 10 min at 4 °C to remove dead cells. The collected supernatant was further centrifuged at 2000*g*, then filtered using a 0.22 μm filter (Millipore) to remove large particles, debris, and aggregates. Ultraclear polycarbonate ultracentrifuge tubes (cat# 355631, Beckman Coulter, UK) were filled with clear conditioned culture media prior to ultracentrifugation onto a sucrose cushion (25% w/w sucrose in D_2_O, density 1.18–1.20 g/mL) at 100,000 g for 1.5 h, 4 °C, using swing-out rotor (SW32 Ti, Beckman Coulter, UK). Consequently, the sucrose solution layer (2 mL/tube) was collected, mixed with 20 mL PBS prefilled in polycarbonate ultracentrifuge tubes (cat. no. 355618; Beckman Coulter, UK), and centrifuged at 100,000 for 1.5 h at 4 °C, using a fixed-angle rotor (70 Ti, Beckman Coulter, UK). The resulting pellets were resuspended in 200 μL of sterile PBS and stored at 4 °C for up to 1 week or at −80 °C for longer storage.

#### Characterization of the Isolated Exosomes

##### Size Distribution, Particle Concentration, and Surface Charge of Exosomes

Exosome size distribution and concentration were measured by nanoparticle tracking analysis (NTA) using a Nanosight LM10 system with blue (488 nm) laser (Malvern Instruments, UK) following a previously published procedure.^73^ Briefly, exosomes were diluted with filtered PBS, and three 60 s videos were captured and analyzed using NanoSight NTA 3.2 software.^74^ Dynamic light scattering (DLS) measurements were performed to assess the hydrodynamic diameter of exosomes by using a Zetasizer Nano ZS system (Malvern Instruments, UK). The surface charge, expressed as zeta potential of the exosomes (diluted in deionized water), was measured using Nanosizer ZS Series (Malvern Instruments, UK).

##### Protein Quantification

Protein quantification of the isolated exosomes was performed using micro-BCA kits in a 96-well plate (Thermo Fisher Scientific, UK), following the previously described protocol.^73^

##### Detection of Exosome Surface Markers Using Flow Cytometry

The detection of exosome markers using flow cytometry was performed following a previously established protocol.^75^ Briefly, exosomes suspended in PBS (50 μL, 10 × 10^10^ particles/mL) were coupled with aldehyde sulfate latex beads (10 μL, 4% w/v) at room temperature for 15 min. Exosome-bead mixture was incubated with BSA (5 μL, 100 μM) for 15 min at room temperature. Afterward, filtered PBS (pH 7.4, 1 mL) was mixed with the whole mixture for 90 min under mild agitation at room temperature. Exosome-bead pellets were collected by centrifugation at 580 *g* for 5 min at room temperature. The obtained pellets were resuspended in glycine (1 mL, 100 mM) for 30 min at room temperature. Consequently, the mixture was centrifuged at 580*g* for 5 min at room temperature. The exosome beads were washed with PBS (pH 7.4, 1 mL) twice and resuspended in PBS (100 μL containing 3% FBS). Beads mixed with PBS and treated with the same protocol were used as a control. The exosome-beads were individually stained with anti-CD9/MRP-1, anti-CD63, and anti-CD81 (1:100 v/v) for 45 min at 4 °C. Subsequently, the mixture was stained with Alexa Fluor 647 Donkey antirabbit IgG 1 secondary antibody (1:100 v/v) for 45 min at 4 °C. The expression of exosome surface markers was determined by mean fluorescence intensity (MFI) (BD FACS Calibur flow cytometer, BD Biosciences) and further analyzed using Flowjo software (Treestar, BD Bioscience, USA).

##### Preparation of Stable Nucleic Acid Lipid Nanoparticles

LNP were fabricated using the ethanol injection method, as previously described (Figure S23).^4,5^ Briefly, the lipid mixture (222.5 nmol) composed of cholesterol, DOPE, Dlin-MC3, and ceramide C16-PEG2000 with a molar ratio of 45:15:35:5, respectively, was dissolved in 50 μL of a mixture of ethanol and 20 mM citrate buffer (pH 4.0) in a ratio of 9:1 v/v. siRNA was dissolved in 20 mM citrate buffer (pH 4.0, 50 μL). The weight ratio of Dlin-MC3 to siRNA is 5:1. Both ethanolic and aqueous phases were heated at 60 °C for 5 min. The lipid mixture was added dropwise (10 μL every 30 s) to the aqueous phase while vortexing. The resulting dispersion was then incubated at 40 °C for 1 h. LNP buffer was exchanged with HEPES buffer (20 mM, pH 7) using Amicon Ultra centrifugal filter units (0.5 mL, 30 kDa Merck Millipore, USA). For fluorescent LNP used in determining fusion efficiency and exosome hybrid marker expression, 1% of the DOPE was replaced by DOPE-FITC. Additionally, LNP for fusion efficiency were stained by incorporating DiI (1 mol % of total lipid) in the ethanolic phase. For the biodistribution study, DiR-labeled LNP were prepared by adding DiR to the lipid mixture at a concentration of 1 mol % of total lipid.

##### Preparation of Exosome Lipid Nanoparticles Hybrid (ELNs)

ELNs were prepared by either freeze–thaw method or sonication method as described elsewhere (Figure 7).^19,60,76,77^ Briefly, exosome (derived from 4T1, B16F10, BL6, CT26 or GL261 cells) (60 μL, ∼5–9 × 10^13^ particles/mL depending on the exosome type) and LNPs (40 μL, 2 × 10^14^ particles/mL) were mixed by vortexing for 1 min and incubated at room temperature for 30 min. The ELNs prepared by the freeze–thaw method were obtained by freezing a mixture of LNPs and exosomes (2:1 particle/particle ratio) at −80 °C for 30 min, followed by thawing at room temperature for 30 min, repeated in 4 cycles. For the ELNs prepared by the sonication method, the mixture of LNP and exosomes (at a 2:1 particle/particle ratio) was incubated in an ultrasound bath at 60 °C for 30 s, followed by immediate incubation in an ice bath for 30 s, repeated in 4 cycles. The prepared ELNs were stored at 4 °C for further assay.

**Figure 7:**
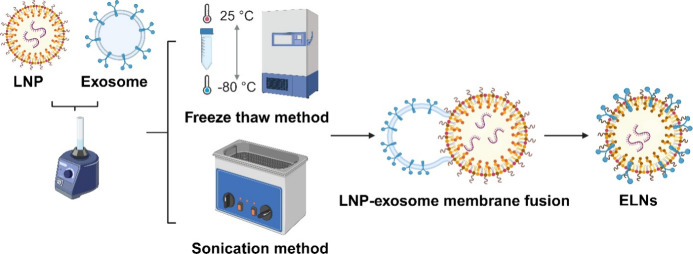
Preparation of exosome lipid nanoparticles hybrid (ELNs) by freeze–thaw and sonication methods. LNPs were prepared by the ethanol injection method using a lipid mixture (cholesterol, DOPE, Dlin-MC3, ceramide C16-PEG2000). siRNA was added with a Dlin-MC3 to siRNA weight ratio of 5:1. After heating both phases at 60 °C for 5 min, the lipid mixture was titrated into the aqueous phase under vortex. After incubation at 40 °C for 1 h, LNP buffer was exchanged with the HEPES buffer. Exosomes and LNP were then mixed by vortexing for 1 min, followed by a 30 min incubation at room temperature. ELNs were prepared either by a freeze–thaw method or sonication method. In the freeze–thaw method, the mixture was frozen at −80 °C and thawed at room temperature in repeated cycles. In the sonication method, the mixture was sonicated at 60 °C, followed by incubation in an ice bath for repeated cycles.

##### Experimental Design and Construction of the Box-Behnken Design (BBD)

Design-Expert software (Design-Expert 13.0.5.0, State-Ease Inc., USA) was utilized to create matrices and explore response surfaces and statistical models for optimizing ELNs prepared by the freeze–thaw or sonication method. Variables selected for each method included the LNP-Exosome ratio (A), number of cycles (B), and duration (C), each at three levels detailed in Tables S1 and S2. ELNs were designed to maximize fusion efficiency % (Y1) and minimize the particle size (Y2). The design matrix comprised 17 formulations for each fabrication method (Tables S3–S6).

##### Determination of Fusion Efficiency

The fusion efficiency % of LNP and exosomes was quantified using a fluorescence resonance energy transfer (FRET) assay.^60^ DOPE-FITC, acting as an electron donor, and DiI, acting as an electron acceptor, were incorporated into the lipid mixture to form FRET LNP. Consequently, ELNs were prepared by either the freeze–thaw method or the sonication method as described earlier. Samples (30 μL) were transferred to a black 96-well plate, and the fluorescence intensity of the fabricated ELNs, diluted in PBS (pH 7.4, 1:3 v/v), was measured using a microplate reader (BMG LABTECH, FLUOstar Omega, UK) at excitation wavelength (λ_exc_) of 485 nm and emission wavelength (λ_emi_) of 520 nm (FITC) and 590 nm (DiI). The fusion efficiency, expressed as % FRET dissolution efficiency, was calculated using the following equation:^60^

$$\%\mathrm{FRET}\;\mathrm{dissolution}\;\mathrm{efficiency}=(\frac{I_{\mathrm{FITC}}}{I_{\mathrm{FITC}}+I_{\mathrm{DiI}}})\times 100$$ 1

where *I*_FITC_ is the emission fluorescence intensity of the donor FITC at 520 nm, and *I*_DiI_ is the emission fluorescence intensity of the acceptor DiI at 590 nm.

##### Determination of the Particle Size and Surface Charge of ELNs

Particle size, polydispersity index (PDI), and zeta potential of the prepared ELNs were measured using a Nanosizer ZS Series (Malvern Instruments, Southborough, MA). Samples were diluted with deionized water (1:100 v/v), and each measurement represented an average of 20 runs conducted in triplicate at 25 °C.^3^ NTA was performed to measure the particle size of the optimized ELNs, following the same protocol used for exosome characterization.

##### Determination of siRNA Entrapment Efficiency % (EE%)

The entrapment efficiency % (EE%) of siRNA in ELNs was quantified indirectly using the Quant-iT RiboGreen assay.^5^ RiboGreen reagent (Thermo Fischer Scientific, UK) was diluted with PBS (1:500 v/v) to obtain the RiboGreen working solution. All PBS and RiboGreen assay solutions were prepared by using RNase-Free water. The prepared ELNs were diluted with PBS (1:50 v/v) and centrifuged using Amicon Ultra centrifugal filter units (0.5 mL, 30 kDa). The filtrate collected after centrifugation was mixed with a RiboGreen working solution in a ratio of 1:1 v/v. Samples (50 μL) were then transferred to a black 96-well plate, and fluorescence intensity was measured at 485 nm excitation and 520 nm emission using a microplate reader (BMG LABTECH, FLUOstar Omega, UK). The percentage EE was calculated using a previously constructed standard siRNA calibration curve within the range of 0.1–5 μg/mL, following this equation:

$$\mathrm{EE}\%=\frac{\mathrm{Initial}\;s\mathrm{iRNA}\;\mathrm{amount}\;\mathrm{added}-\mathrm{measured}\;\mathrm{free}\;s\mathrm{iRNA}}{\mathrm{Initial}\;s\mathrm{iRNA}\;\mathrm{amount}\;\mathrm{added}}\times 100$$ 2

##### Transmission Electron Microscope of ELNs

Exosome suspensions were fixed in 2% (w/v) formaldehyde in deionized water for 15 min. Carbon-coated copper TEM grids were rendered hydrophilic by glow discharge for 60 s (PELCO easiGlow). A 2.5 μL aliquot of each sample (exosomes, LNP, or ELNs) was applied to the grid for 120 s and gently blotted with filter paper. Grids were then negatively stained with 2% (w/v) phosphotungstic acid for 20 s and blotted again. Imaging was performed on a Talos L120C transmission electron microscope (Thermo Fisher Scientific) operated at 120 kV. Micrographs were collected at cryogenic temperatures using a Ceta CMOS camera and Velox software.

##### Detection of Exosome Surface Marker On the Optimized ELNs

The optimized ELNs, prepared using either freeze–thaw or sonication methods, were labeled with DOPE-FITC in place of DOPE. The fluorescently labeled ELNs were coupled with aldehyde/sulfate latex beads following the method described earlier for surface marker detection. The exosome-bead complexes were subsequently stained using a two-step labeling process: primary antibodies (anti-CD9, anti-CD63, or anti-CD81 at 1:100 v/v) followed by Alexa Fluor 647 Donkey antirabbit IgG secondary antibody (1:100 v/v) as described above. Samples were then analyzed based on mean fluorescence intensity (MFI). The expression of exosome surface markers on ELNs was confirmed by colocalized positive staining.

##### Cellular Association of the Optimized ELNs

The cellular association of the optimized ELNs from the five tested exosomes was assessed in 4T1, B16F10, BL6, CT26, and GL261 cells. Confluent cells were incubated with the optimized ELNs, prepared either by a freeze–thaw or sonication method, loaded with Atto740 siRNA at a concentration of 30 nM, for 24 h. Afterward, the cells were washed with PBS twice and trypsinized. The collected cells were centrifuged at 1750 rpm for 3 min at 4 °C. The cellular association of Atto740 siRNA was quantified by measuring mean fluorescence intensity (MFI) with a minimum of 10,000 cell events using flow cytometry (BD FACS Calibur flow cytometer, BD Biosciences, USA). The analysis was performed using Flowjo software (Treestar, BD Biosciences, USA).

##### Detection of Surface Targets on Different Cancer Cells

Surface expressions of CD24, CD44, and CD47 on 4T1, B16F10, BL6, CT26, and GL261 cells were quantified using flow cytometry. Cells were stained individually with either antimouse CD24-PE, antimouse CD44-APC, and antimouse CD47-APC monoclonal antibody (1:100 v/v), and the expression of CD24, CD44, and CD47 was quantified based on mean fluorescence intensity (MFI) (BD FACS Calibur flow cytometer, BD Biosciences, USA). Data were further analyzed by Flowjo software (Treestar, BD Biosciences, USA).

##### In Vitro Gene Silencing

To evaluate the homing ability of ELNs, different cell types were treated with the optimized ELNs prepared by either the freeze–thaw or sonication method, loaded with siCD44 (30 nM) for 48 h. For comparison, cells were treated with LNP containing siCD44 at the same dose. After treatment, the media were aspirated, and the cells were washed with PBS twice and collected by trypsin-EDTA. The cells were then stained with antimouse CD44-APC, and the expression of CD44 was measured as the percentage of positive cells. Additionally, the in vitro silencing efficiency of the selected ELNs in both 4T1 and B16F10 cells was assessed for target dual-gene knockdown. Briefly, 4T1 cells and B16F10 were incubated with the optimized ELNs loaded with siCD44 and siCD47, with and without siCD24, respectively, at concentrations of 10, 20, and 30 nM for 48 h. Consequently, cells were collected and stained for marker expression.

##### Uptake of 4T1 and B16F10 Cancer Cells by Macrophages in a Coculture Study

The ability to down-regulate “do not eat me” signals, CD24 and CD47, to enhance macrophage uptake was tested by flow cytometry. Both 4T1 cells and B16F10 cells were stained with CellTrace before incubation with the corresponding ELNs for 48 h. 4T1 cells were transfected with 30 nM siCD24, siCD44, siCD47 or their combination, while B16F10 cells were transfected with 30 nM siCD44, siCD47 or their combination. After transfection, cells were collected and cocultured with J774A.1 macrophage cells for 6 h. Subsequently, cells were harvested, stained with antimouse CD45 monoclonal antibody, and analyzed using a FACS Calibur flow cytometer. For analysis, cells were first gated based on CD45 expression, and then CellTrace fluorescence was quantified within the CD45+ population.^3^

##### In Vivo Imaging and Organ Biodistribution

All animal studies are conducted with project and personal licenses granted by the UK Home Office and in accordance with the UK Animals (Scientific Procedures) Act 1986 and UK Home Office Code of Practice for the Housing and Care of Animals Used in Scientific Procedures (Home Office 1989). In vivo experiments were conducted in accordance with the project license approved by the King’s College London Animal Welfare and Ethical Review Body (AWERB) and the UK Home Office (PBE6EB195 and PP8950634). BALB/c and C57BL/6 mice, 4–6 weeks (Envigo), were inoculated subcutaneously in the lower flank with 4T1 cells and B16F10 cells, respectively (1 × 10^6^ cells/mouse in PBS). Once the tumors reached approximately 70–80 mm^3^, the mice were anesthetized with isoflurane. Animals were i.v. injected with 100 μL of either DiR-labeled (1 mol % of total lipid) optimized ELNs or LNP in HEPES buffer (pH 7) containing approximately 1 nmol of siNEG. Whole-body images were captured at 1, 4, and 24 h postinjection using the IVIS Lumina Series III In Vivo Imaging System (PerkinElmer, USA) with excitation/emission wavelengths of 740/790 nm. Uninjected animals served as controls. Animals were sacrificed following 24 h of injection, and their tumors, along with vital organs (brain, heart, lung, liver, spleen, and kidneys), were collected, weighed, and imaged.

##### In Vivo Therapeutic Study

BALB/c and C57BL/6 mice (*n* = 6 per group) were implanted subcutaneously with 1 × 10^6^ 4T1 cells or B16F10 cells, respectively. On days 8 and 14, BALB/c mice were i.v. injected with either PBS, LNP, or the corresponding ELNs with either siNEG or a combination of siCD24, siCD44, and siCD47 in a dose of 33.3 μg/kg in the 4T1 tumor model. On days 6 and 9, C57BL/6 mice were i.v. injected with either PBS, LNP, or the optimized ELNs containing either siNEG or siCD44 and siCD47 in the B16F10 melanoma tumor model at a dose of 33.3 μg/kg each. The tumour size was monitored for each mouse until sacrifice. The experiment was terminated when control group mice reached their humane end points, (tumor length >15 mm, body weight loss of 15%) in accordance with institutional ethical guidelines and approved study protocols. Subsequently, the weights of individual organs, tumors, and lymph nodes were recorded.

##### Statistical Analysis

For all in vitro experiments, each experiment was conducted in triplicate with data presented as mean ± standard deviation (SD). For the in vivo therapy study, data were presented as mean (*n* = 6) ± SD. Statistical comparisons between two variables were made using Student’s *t*-test, followed by the Mann–Whitney post-test, while comparisons between groups were conducted using ANOVA followed by Tukey’s HSD test. A *p*-value of less than 0.05 was considered statistically significant. All graphs (except those resulting from BBD analyses) were created by using Prism 10.2.3.

## Supplementary Material



## Notes

Flow cytometry characterization of surface markers (CD9, CD63, CD81) on exosomes and ELNs prepared by freeze–thaw and sonication methods; CPPs, CQAs and QTPP for ELNs fabrication; DoE matrices for all cell-line–derived ELNs; correlation between actual and predicted values for fusion efficiency and particle size of different ELNs; model performance statistics and predicted equations for fusion efficiency and particle size; pareto charts showing the influence of key process parameters on ELNs; main-effect plots for parameter significance across all ELN types; 3D response-surface plots illustrating interactions affecting fusion efficiency and particle size; experimental vs predicted values for optimized ELNs; DLS size-distribution profiles of optimized ELNs; CD24, CD44, CD47 expression panels for all tested cancer cell lines; cellular association quantification of siRNA-loaded ELNs versus LNPs; CD44 silencing efficiency of ELNs versus LNPs across all parent cell types, concentration-dependent downregulation of CD24, CD44, and CD47 using optimized FT-ELNs; in vivo tumor growth inhibition study details including tumor images and organ-weight data; exosome isolation schematic using sucrose-cushion ultracentrifugation; and preparation schematic of lipid nanoparticles (LNPs) using the ethanol-injection method (PDF)

## References

[1] Choi Y., Seok S. H., Yoon H. Y., Ryu J. H., Kwon I. C. (2024). Advancing cancer immunotherapy through siRNA-based gene silencing for immune checkpoint blockade. Adv. Drug Delivery Rev..

[2] Hu B., Zhong L., Weng Y., Peng L., Huang Y., Zhao Y., Liang X.-J. (2020). Therapeutic siRNA: state of the art. Signal Transduction and Targeted Therapy.

[3] Abdel-Bar H. M., Walters A. A., Lim Y., Rouatbi N., Qin Y., Gheidari F., Han S., Osman R., Wang J. T., Al-Jamal K. T. (2021). An ’eat me’ combinatory nano-formulation for systemic immunotherapy of solid tumors. Theranostics.

[4] Walters A. A., Santacana-Font G., Li J., Routabi N., Qin Y., Claes N., Bals S., Wang J. T.-W., Al-Jamal K. T. (2021). Nanoparticle-Mediated In Situ Molecular Reprogramming of Immune Checkpoint Interactions for Cancer Immunotherapy. ACS Nano.

[5] Qin Y., Walters A. A., Rouatbi N., Wang J. T., Abdel-Bar H. M., Al-Jamal K. T. (2023). Evaluation of a DoE based approach for comprehensive modelling of the effect of lipid nanoparticle composition on nucleic acid delivery. Biomaterials.

[6] Hald Albertsen C., Kulkarni J. A., Witzigmann D., Lind M., Petersson K., Simonsen J. B. (2022). The role of lipid components in lipid nanoparticles for vaccines and gene therapy. Adv. Drug Deliv Rev..

[7] Wang J., Ding Y., Chong K., Cui M., Cao Z., Tang C., Tian Z., Hu Y., Zhao Y., Jiang S. (2024). Recent Advances in Lipid Nanoparticles and Their Safety Concerns for mRNA Delivery. Vaccines (Basel).

[8] Sun D., Lu Z. R. (2023). Structure and Function of Cationic and Ionizable Lipids for Nucleic Acid Delivery. Pharm. Res..

[9] Graham R., Gazinska P., Zhang B., Khiabany A., Sinha S., Alaguthurai T., Flores-Borja F., Vicencio J., Beuron F., Roxanis I. (2023). Serum-derived extracellular vesicles from breast cancer patients contribute to differential regulation of T-cell-mediated immune-escape mechanisms in breast cancer subtypes. Front. Immunol..

[10] Xu L., Faruqu F. N., Lim Y. M., Lim K. Y., Liam-Or R., Walters A. A., Lavender P., Fear D., Wells C. M., Tzu-Wen Wang J. (2021). Exosome-mediated RNAi of PAK4 prolongs survival of pancreatic cancer mouse model after loco-regional treatment. Biomaterials.

[11] Xu L., Faruqu F. N., Liam-Or R., Abu Abed O., Li D., Venner K., Errington R. J., Summers H., Wang J. T., Al-Jamal K. T. (2020). Design of experiment (DoE)-driven in vitro and in vivo uptake studies of exosomes for pancreatic cancer delivery enabled by copper-free click chemistry-based labelling. J. Extracell. Vesicles.

[12] Li J., Wang J., Chen Z. (2025). Emerging role of exosomes in cancer therapy: progress and challenges. Mol. Cancer.

[13] Ye J., Li D., Jie Y., Luo H., Zhang W., Qiu C. (2024). Exosome-based nanoparticles and cancer immunotherapy. Biomedicine & Pharmacotherapy.

[14] Kim H. I., Park J., Zhu Y., Wang X., Han Y., Zhang D. (2024). Recent advances in extracellular vesicles for therapeutic cargo delivery. Experimental & Molecular Medicine.

[15] Takakura Y., Hanayama R., Akiyoshi K., Futaki S., Hida K., Ichiki T., Ishii-Watabe A., Kuroda M., Maki K., Miura Y. (2024). Quality and Safety Considerations for Therapeutic Products Based on Extracellular Vesicles. Pharm. Res..

[16] Kalluri R., LeBleu V. S. (2020). The biology, function, and biomedical applications of exosomes. Science.

[17] Kumar M. A., Baba S. K., Sadida H. Q., Marzooqi S. A., Jerobin J., Altemani F. H., Algehainy N., Alanazi M. A., Abou-Samra A.-B., Kumar R. (2024). Extracellular vesicles as tools and targets in therapy for diseases. Signal Transduction and Targeted Therapy.

[18] Tenchov R., Sasso J. M., Wang X., Liaw W. S., Chen C. A., Zhou Q. A. (2022). Exosomes—Nature’s Lipid Nanoparticles, a Rising Star in Drug Delivery and Diagnostics. ACS Nano.

[19] Li L., He D., Guo Q., Zhang Z., Ru D., Wang L., Gong K., Liu F., Duan Y., Li H. (2022). Exosome-liposome hybrid nanoparticle codelivery of TP and miR497 conspicuously overcomes chemoresistant ovarian cancer. J. Nanobiotechnol..

[20] Liu A., Yang G., Liu Y., Liu T. (2022). Research progress in membrane fusion-based hybrid exosomes for drug delivery systems. Front. Bioeng. Biotechnol..

[21] Wang X., Li D., Li G., Chen J., Yang Y., Bian L., Zhou J., Wu Y., Chen Y. (2024). Enhanced Therapeutic Potential of Hybrid Exosomes Loaded with Paclitaxel for Cancer Therapy. International Journal of Molecular Sciences.

[22] Ducrot C., Loiseau S., Wong C., Madec E., Volatron J., Piffoux M. (2023). Hybrid extracellular vesicles for drug delivery. Cancer Letters.

[23] Lin Y., Wu J., Gu W., Huang Y., Tong Z., Huang L., Tan J. (2018). Exosome-Liposome Hybrid Nanoparticles Deliver CRISPR/Cas9 System in MSCs. Adv. Sci..

[24] Soltanmohammadi F., Gharehbaba A. M., Zangi A. R., Adibkia K., Javadzadeh Y. (2024). Current knowledge of hybrid nanoplatforms composed of exosomes and organic/inorganic nanoparticles for disease treatment and cell/tissue imaging. Biomedicine & Pharmacotherapy.

[25] Xu X., Xu L., Wen C., Xia J., Zhang Y., Liang Y. (2023). Programming assembly of biomimetic exosomes: An emerging theranostic nanomedicine platform. Materials Today Bio.

[26] Zheng L., Hu B., Zhao D., Liu W., Liu Q., Huang Y., Ruan S. (2024). Recent progresses of exosome–liposome fusions in drug delivery. Chin. Chem. Lett..

[27] Abdel-Bar H. M., Walters A. A., Wang J. T., Al-Jamal K. T. (2021). Combinatory Delivery of Etoposide and siCD47 in a Lipid Polymer Hybrid Delays Lung Tumor Growth in an Experimental Melanoma Lung Metastatic Model. Adv. Healthc. Mater..

[28] Rouatbi N., Walters A. A., Zam A., Lim Y. M., Marrocu A., Liam-Or R., Anstee J. E., Arnold J. N., Wang J. T., Pollard S. M., Al-Jamal K. T. (2025). CD47 Knock-Out Using CRISPR-Cas9 RNA Lipid Nanocarriers Results in Reduced Mesenchymal Glioblastoma Growth In Vivo. Adv. Sci..

[29] Lau A. P. Y., Khavkine Binstock S. S., Thu K. L. (2023). CD47: The Next Frontier in Immune Checkpoint Blockade for Non-Small Cell Lung Cancer. Cancers (Basel).

[30] Wang H., Shi P., Shi X., Lv Y., Xie H., Zhao H. (2023). Surprising magic of CD24 beyond cancer. Front. Immunol..

[31] Yang Y., Zhu G., Yang L., Yang Y. (2023). Targeting CD24 as a novel immunotherapy for solid cancers. Cell Communication and Signaling.

[32] Altevogt P., Sammar M., Hüser L., Kristiansen G. (2021). Novel insights into the function of CD24: A driving force in cancer. Int. J. Cancer.

[33] Zhao K., Wu C., Li X., Niu M., Wu D., Cui X., Zhao H. (2024). From mechanism to therapy: the journey of CD24 in cancer. Front. Immunol..

[34] Li B., Zhao T., Shao M., Cai J., Chen S., Chen X., Yang M., Zheng Y., Cui C., Guo S., Yang Z., Ren F., Jia H. (2023). Attenuated Salmonella carrying siRNA-CD24 improved the effect of oxaliplatin on HCC. Int. Immunopharmacol..

[35] Su D., Deng H., Zhao X., Zhang X., Chen L., Chen X., Li Z., Bai Y., Wang Y., Zhong Q. (2009). Targeting CD24 for treatment of ovarian cancer by short hairpin RNA. Cytotherapy.

[36] Sagiv E., Starr A., Rozovski U., Khosravi R., Altevogt P., Wang T., Arber N. (2008). Targeting CD24 for Treatment of Colorectal and Pancreatic Cancer by Monoclonal Antibodies or Small Interfering RNA. Cancer Res..

[37] Okabe H., Aoki K., Yogosawa S., Saito M., Marumo K., Yoshida K. (2018). Downregulation of CD24 suppresses bone metastasis of lung cancer. Cancer Science.

[38] Magenau J., Jaglowski S., Uberti J., Farag S. S., Riwes M. M., Pawarode A., Anand S., Ghosh M., Maciejewski J., Braun T. (2024). A phase 2 trial of CD24Fc for prevention of graft-versus-host disease. Blood.

[39] Xu H., Niu M., Yuan X., Wu K., Liu A. (2020). CD44 as a tumor biomarker and therapeutic target. Exp. Hematol. Oncol..

[40] Ponta H., Sherman L., Herrlich P. A. (2003). CD44: From adhesion molecules to signalling regulators. Nat. Rev. Mol. Cell Biol..

[41] Wu B., Shi X., Jiang M., Liu H. (2023). Cross-talk between cancer stem cells and immune cells: potential therapeutic targets in the tumor immune microenvironment. Mol. Cancer.

[42] Martincuks A., Li P. C., Zhao Q., Zhang C., Li Y. J., Yu H., Rodriguez-Rodriguez L. (2020). CD44 in Ovarian Cancer Progression and Therapy Resistance-A Critical Role for STAT3. Front. Oncol..

[43] Liang Y., Wang Y., Wang L., Liang Z., Li D., Xu X., Chen Y., Yang X., Zhang H., Niu H. (2021). Self-crosslinkable chitosan-hyaluronic acid dialdehyde nanoparticles for CD44-targeted siRNA delivery to treat bladder cancer. Bioactive Materials.

[44] Kim H. K., Quan Y. H., Lim J.-Y., Choi B. H., Park J.-H., Choi Y. H. (2017). P2.01–013 HA-Liposome Nanocarrier Containing CD44 siRNA as a Targeted Chemotherapy to CD44 Related Chemoresistant Non-Small Cell Lung Cancer: Topic: Analysis of RNA. J. Thorac. Oncol..

[45] Khan M. A., Anand S., Deshmukh S. K., Singh S., Singh A. P. (2022). Determining the Size Distribution and Integrity of Extracellular Vesicles by Dynamic Light Scattering. Methods Mol. Biol..

[46] Maas S. L. N., de Vrij J., van der Vlist E. J., Geragousian B., van Bloois L., Mastrobattista E., Schiffelers R. M., Wauben M. H. M., Broekman M. L. D., Nolte-’t Hoen E. N. M. (2015). Possibilities and limitations of current technologies for quantification of biological extracellular vesicles and synthetic mimics. J. Controlled Release.

[47] Ahmadi S. E., Soleymani M., Shahriyary F., Amirzargar M. R., Ofoghi M., Fattahi M. D., Safa M. (2023). Viral vectors and extracellular vesicles: innate delivery systems utilized in CRISPR/Cas-mediated cancer therapy. Cancer Gene Ther..

[48] Chernyshev V. S., Rachamadugu R., Tseng Y. H., Belnap D. M., Jia Y., Branch K. J., Butterfield A. E., Pease L. F., Bernard P. S., Skliar M. (2015). Size and shape characterization of hydrated and desiccated exosomes.. Anal Bioanal Chem..

[49] Abo El-Enin H. A., Tulbah A. S., Darwish H. W., Salama R., Naguib I. A., Yassin H. A., Abdel-Bar H. M. (2023). Evaluation of Brain Targeting and Antipsychotic Activity of Nasally Administrated Ziprasidone Lipid-Polymer Hybrid Nanocarriers. Pharmaceuticals (Basel).

[50] Li Y.-J., Wu J.-Y., Liu J., Xu W., Qiu X., Huang S., Hu X.-B., Xiang D.-X. (2021). Artificial exosomes for translational nanomedicine. J. Nanobiotechnol..

[51] Liang Y., Xu X., Xu L., Iqbal Z., Ouyang K., Zhang H., Wen C., Duan L., Xia J. (2022). Chondrocyte-specific genomic editing enabled by hybrid exosomes for osteoarthritis treatment. Theranostics.

[52] Richardson E. S., Pitt W. G., Woodbury D. J. (2007). The role of cavitation in liposome formation. Biophys. J..

[53] Hoshyar N., Gray S., Han H., Bao G. (2016). The effect of nanoparticle size on in vivo pharmacokinetics and cellular interaction. Nanomedicine (Lond).

[54] Chen W., Wang W., Xie Z., Centurion F., Sun B., Paterson D. J., Tsao S. C.-H., Chu D., Shen Y., Mao G. (2024). Size-Dependent Penetration of Nanoparticles in Tumor Spheroids: A Multidimensional and Quantitative Study of Transcellular and Paracellular Pathways. Small.

[55] Akinc A., Maier M. A., Manoharan M., Fitzgerald K., Jayaraman M., Barros S., Ansell S., Du X., Hope M. J., Madden T. D. (2019). The Onpattro story and the clinical translation of nanomedicines containing nucleic acid-based drugs. Nat. Nanotechnol..

[56] Xu J., Song M., Fang Z., Zheng L., Huang X., Liu K. (2023). Applications and challenges of ultra-small particle size nanoparticles in tumor therapy. J. Controlled Release.

[57] Mukherjee A., Bisht B., Dutta S., Paul M. K. (2022). Current advances in the use of exosomes, liposomes, and bioengineered hybrid nanovesicles in cancer detection and therapy. Acta Pharmacol. Sin..

[58] Karmacharya M., Kumar S., Cho Y.-K. (2023). Tuning the Extracellular Vesicles Membrane through Fusion for Biomedical Applications. Journal of Functional Biomaterials.

[59] Abo El-Enin H. A., Ahmed M. F., Naguib I. A., El-Far S. W., Ghoneim M. M., Alsalahat I., Abdel-Bar H. M. (2022). Utilization of Polymeric Micelles as a Lucrative Platform for Efficient Brain Deposition of Olanzapine as an Antischizophrenic Drug via Intranasal Delivery. Pharmaceuticals (Basel).

[60] Sato Y. T., Umezaki K., Sawada S., Mukai S.-a., Sasaki Y., Harada N., Shiku H., Akiyoshi K. (2016). Engineering hybrid exosomes by membrane fusion with liposomes. Sci. Rep..

[61] Ishikawa R., Yoshida S., Sawada S. I., Sasaki Y., Akiyoshi K. (2022). Development and single-particle analysis of hybrid extracellular vesicles fused with liposomes using viral fusogenic proteins. FEBS Open Bio.

[62] Zhang W., Ngo L., Tsao S. C.-H., Liu D., Wang Y. (2023). Engineered Cancer-Derived Small Extracellular Vesicle-Liposome Hybrid Delivery System for Targeted Treatment of Breast Cancer. ACS Appl. Mater. Interfaces.

[63] Karami M. H., Pourmadadi M., Abdouss M., Kalaee M. R., Moradi O., Rahdar A., Díez-Pascual A. M. (2023). Novel chitosan/γ-alumina/carbon quantum dot hydrogel nanocarrier for targeted drug delivery. Int. J. Biol. Macromol..

[64] Jhan Y.-Y., Prasca-Chamorro D., Palou Zuniga G., Moore D. M., Arun Kumar S., Gaharwar A. K., Bishop C. J. (2020). Engineered extracellular vesicles with synthetic lipids via membrane fusion to establish efficient gene delivery. Int. J. Pharm..

[65] duPre S. A., Hunter K. W. (2007). Murine mammary carcinoma 4T1 induces a leukemoid reaction with splenomegaly: Association with tumor-derived growth factors. Exp. Mol. Pathol..

[66] Evers M. J. W., van de Wakker S. I., de Groot E. M., de Jong O. G., Gitz-François J. J. J., Seinen C. S., Sluijter J. P. G., Schiffelers R. M., Vader P. (2022). Functional siRNA Delivery by Extracellular Vesicle-Liposome Hybrid Nanoparticles. Adv. Healthcare Mater..

[67] Zhou X., Miao Y., Wang Y., He S., Guo L., Mao J., Chen M., Yang Y., Zhang X., Gan Y. (2022). Tumour-derived extracellular vesicle membrane hybrid lipid nanovesicles enhance siRNA delivery by tumour-homing and intracellular freeway transportation. Journal of Extracellular Vesicles.

[68] Zhang Y., Luo X., Ding N., Belaid M., Thanou M., Vllasaliu D. (2024). Hybrid Milk Extracellular Vesicles as Potential Systems for Oral Delivery of siRNA. Advanced Therapeutics.

[69] Li L., Gong Y., Tang J., Yan C., Li L., Peng W., Cheng Z., Yu R., Xiang Q., Deng C. (2022). ZBTB28 inhibits breast cancer by activating IFNAR and dual blocking CD24 and CD47 to enhance macrophages phagocytosis. Cell. Mol. Life Sci..

[70] Yang Y., Wu H., Yang Y., Kang Y., He R., Zhou B., Guo H., Zhang J., Li J., Ge C. (2023). Dual blockade of CD47 and CD24 signaling using a novel bispecific antibody fusion protein enhances macrophage immunotherapy. Mol. Ther. Oncolytics.

[71] Shen Y.-A., Wang C.-Y., Chuang H.-Y., Hwang J. J.-J., Chi W.-H., Shu C.-H., Ho C.-Y., Li W.-Y., Chen Y.-J. (2016). CD44 and CD24 coordinate the reprogramming of nasopharyngeal carcinoma cells towards a cancer stem cell phenotype through STAT3 activation. Oncotarget.

[72] Gangoso E., Southgate B., Bradley L., Rus S., Galvez-Cancino F., McGivern N., Güç E., Kapourani C. A., Byron A., Ferguson K. M. (2021). Glioblastomas acquire myeloid-affiliated transcriptional programs via epigenetic immunoediting to elicit immune evasion. Cell.

[73] Liam-Or R., Faruqu F. N., Walters A., Han S., Xu L., Wang J. T.-W., Oberlaender J., Sanchez-Fueyo A., Lombardi G., Dazzi F. (2024). Cellular uptake and in vivo distribution of mesenchymal-stem-cell-derived extracellular vesicles are protein corona dependent. Nat. Nanotechnol..

[74] Khan A. A., Man F., Faruqu F. N., Kim J., Al-Salemee F., Carrascal-Miniño A., Volpe A., Liam-Or R., Simpson P., Fruhwirth G. O. (2022). PET Imaging of Small Extracellular Vesicles via [89Zr]­Zr­(oxinate)­4 Direct Radiolabeling. Bioconjugate Chem..

[75] Al-Jamal K. T., Faruqu F. N., Xu L. (2018). Preparation of Exosomes for siRNA Delivery to Cancer Cells. JoVE.

[76] Lv Q., Cheng L., Lu Y., Zhang X., Wang Y., Deng J., Zhou J., Liu B., Liu J. (2020). Thermosensitive Exosome-Liposome Hybrid Nanoparticle-Mediated Chemoimmunotherapy for Improved Treatment of Metastatic Peritoneal Cancer. Advanced Science.

[77] Sun L., Fan M., Huang D., Li B., Xu R., Gao F., Chen Y. (2021). Clodronate-loaded liposomal and fibroblast-derived exosomal hybrid system for enhanced drug delivery to pulmonary fibrosis. Biomaterials.

