# Supplementary material for: Optimizing Exosome Lipid Hybrid Nanoparticles for Enhanced siRNA Delivery and Improved Therapeutic Anticancer Efficacy In Vivo
Source: ACS Nano. 2025 Dec 11;19(50):42658–74. doi: 10.1021/acsnano.5c16991 (PMC12774390; doi:10.1021/acsnano.5c16991)
Supplement: Supplementary file 1 [file nn5c16991_si_001.pdf]

Optimizing Exosome Lipid Hybrid Nanoparticles for Enhanced siRNA Delivery and Improved Therapeutic Anticancer Efficacy *In Vivo*

Hend Mohamed Abdel-Bar, [†, ‡] Steven Tandiono, [†] Revadee Liam-Or, [†, §] Calvin C. L. Cheung, [§] Osama W. M. Hassuneh, [§] Qingyang Lyu, [§] Yue Qin, [†] Shunping Han, [†] Nadia Rouatbi, [†] Julie Tzu-Wen Wang, [†] Adam A. Walters, [†] Khuloud T. Al-Jamal, [*, †, §]

[†] Institute of Pharmaceutical Science, King's College London, Franklin-Wilkins Building, 150 Stamford Street, London SE1 9NH, UK

[‡] Department of Pharmaceutics, Faculty of Pharmacy, University of Sadat City, P.O. box: 32958 Egypt

[§] Department of Pharmacology and Pharmacy, Li Ka Shing Faculty of Medicine, The University of Hong Kong, Hong Kong Special Administrative Region, China

* Corresponding authors

E-mail: khuloud.al-jamal@kcl.ac.uk

Keywords: Extracellular vesicles, Lipid nanoparticles, DoE, triple negative breast cancer, CD24, CD44, CD47.

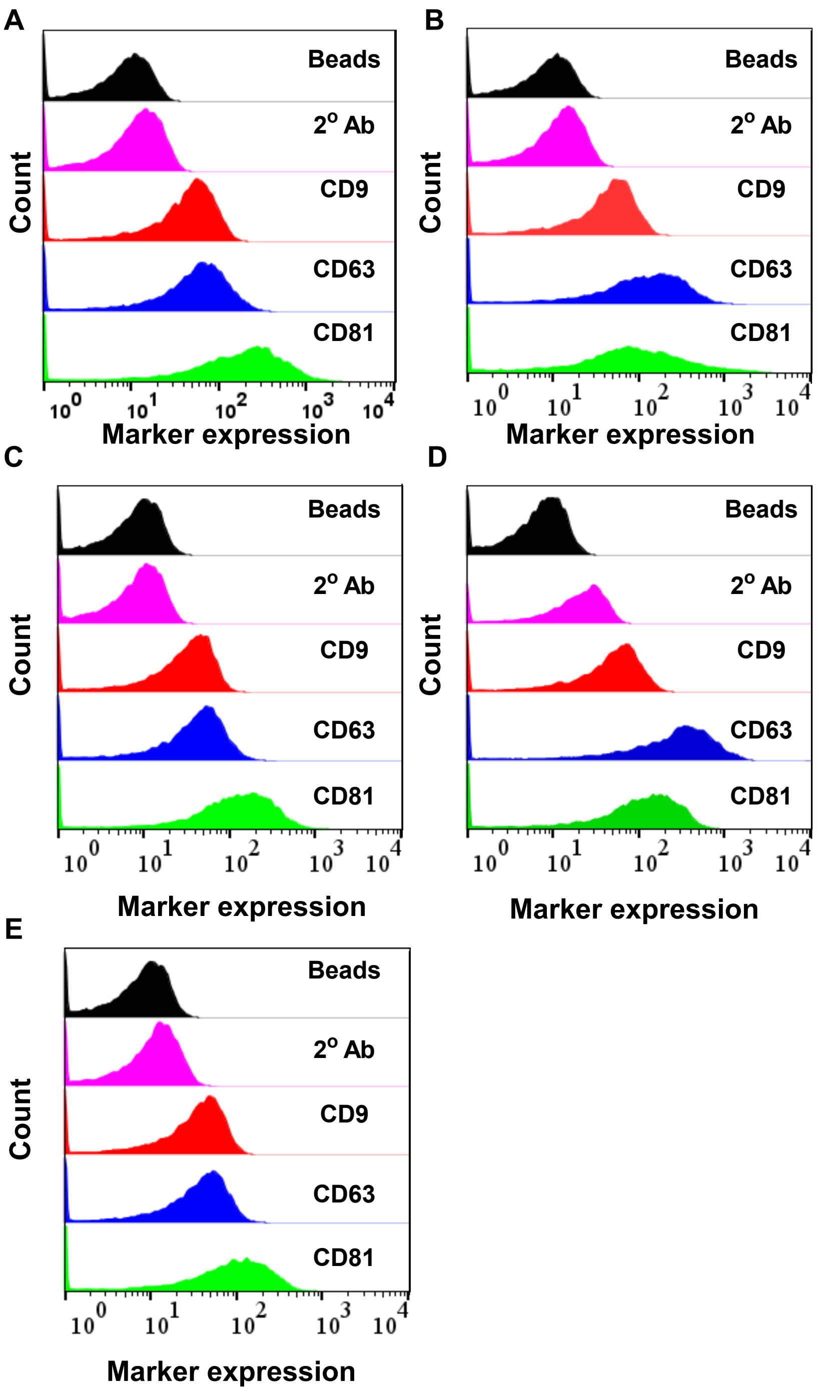

Figure S1: Characterization of the isolated exosomes markers. Detection of tetraspanins (CD9, CD63, and CD81) on exosomes by flow cytometry. Exosomes coupled to aldehyde/sulfate latex beads were stained with primary antibodies (anti-CD9, anti-CD63, or anti-CD81, 1:100 v/v) followed by Alexa Fluor 647 Donkey anti-rabbit IgG secondary antibody (1:100 v/v). MFI values were used to visualize variations in tetraspanin expression levels among different exosome types. Representative flow cytometry histograms displaying tetraspanin expression on the surface of **(A)** 4T1 exosomes, **(B)** B16F10, **(C)** BL6, **(D)** CT26, **(E)** GL261. Black and pink filled histograms represent unstained and secondary antibody-only controls, respectively. Red, blue, or green filled histograms indicate tetraspanin expression levels. Mean fluorescence intensity (MFI), measuring brightness, provides a relative quantification of marker expression. The obtained data revealed highlights the predominant abundance of tetraspanins on exosomes derived from different cancer cells.

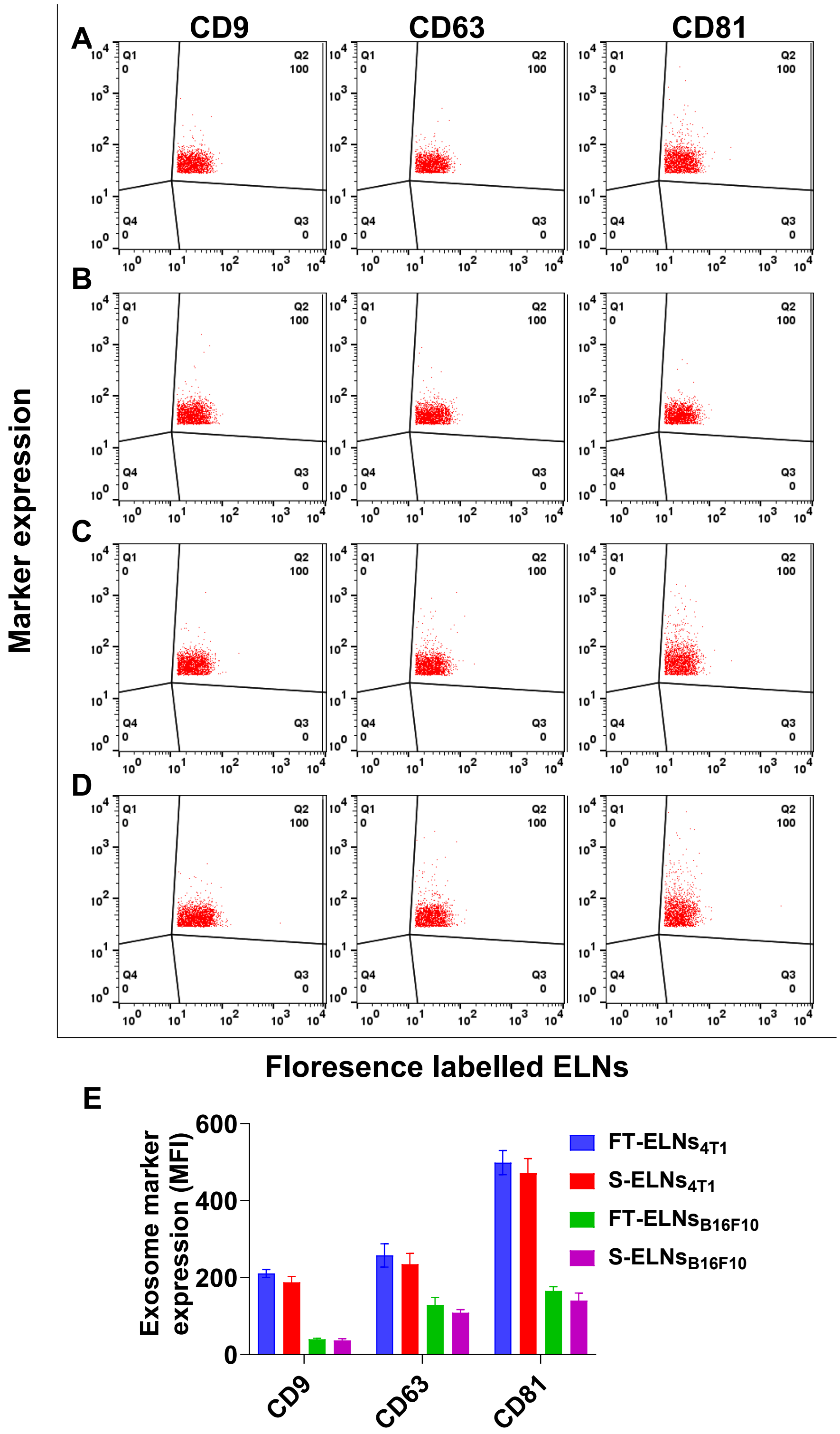

Figure S2: The optimized ELNs expressed the characteristic surface exosome markers. Both ELNs$_{4T1}$ and ELNs$_{B16F10}$ prepared either by freeze-thaw **(A, B)** or sonication method **(C, D)** were prepared by replacing DOPE with DOPE-FITC. ELNs were formed by mixing LNP and exosomes at a 2:1 particle ratio, followed by either freeze–thaw cycles (4 cycles at -80 °C for 30 min then room temperature for 30 min) or sonication cycles (4 cycles at 60 °C for 30 sec followed by 30 sec on ice). The florescence labelled ELNs were coupled to aldehyde/sulphate latex beads prior to detection. Exosome-beads complex were subsequently stained using a 2-step labelling (anti-CD9, anti-CD63 or anti-CD81 1^{ry} Ab 1:100 v/v and Alexa Fluor 647 Donkey antirabbit Ig G 2^{ry} Ab 1:100 v/v. Samples were then analysed using FL-1 and FL-4 channels. Double positive staining indicated the expression of exosome markers on ELNs surface. **(E)** MFI values showed a consistent expression of exosome markers across ELNs fabricated using both methods.

Table S1: Levels of CPPs, CQAs and QTPP for the Preparation of ELNs by Freeze-Thaw Method.

Factors (Coded independent variables)	Levels		
	Low (-1)	Medium (0)	High (1)
A: LNP-Exosome ratio [a]	2	11	20
B: Number of cycles [b]	3	4	5
C: Duration (min)	15	22.5	30
Responses	Constraints		
Y1: Fusion efficiency (%) [c]	Maximize		
Y2: Particle size (nm) [d]	Minimize		

[a] calculated as particles/ particles measured by using nanoparticle tracking analysis (NTA).
[b] each cycle is composed of freezing at -80 °C followed by defrosting at room temperature.
[c] calculated by measuring fluorescence resonance energy transfer (FRET) dissolution efficiency.
[d] measured using dynamic light scattering technique (DLS).

Table S2: Levels of CPPs, CQAs and QTPP for the Preparation of ELNs by Sonication Method.

Factors (Coded independent variables)	Levels		
	Low (-1)	Medium (0)	High (1)
A: LNP-Exosome ratio [a]	2	11	20
B: Number of cycles [b]	3	4	5
C: Duration (Sec)	30	45	60
Responses	Constraints		
Y1: Fusion efficiency (%) [c]	Maximize		
Y2: Particle size (nm) [d]	Minimize		

[a] calculated as particles/ particles measured by using nanoparticle tracking analysis (NTA).
[b] each cycle is composed of sonication at 60 °C followed by incubation in ice.
[c] calculated by measuring fluorescence resonance energy transfer (FRET) dissolution efficiency.
[d] measured using dynamic light scattering technique (DLS).

Table S3: Experimental Design Matrix of the CPPs and the Related CQAs of $ELNs_{4T1}$ and $ELNs_{B16F10}$ Prepared by Freeze-Thaw Method.

Run	A: LNP-Exosome ratio [a]	B: Number of cycles [b]	C: Duration (min)	$ELNs_{4T1}$		$ELNs_{B16F10}$	
				Fusion efficiency (%) [c, e]	Particle size (nm) [d, e]	Fusion efficiency (%) [c, e]	Particle size (nm) [d, e]
1	2	4	30	91.4±3.5	147.6±5.3	83.5±5.2	116.6±8.3
2	20	4	30	58.6±4.6	219.7±4.9	58.3±6.7	176.8±4.3
3	11	4	22.5	85.9±2.6	132.3±4.6	80.4±4.7	104.5±6.9
4	20	5	22.5	82.5±1.9	248.5±10.3	81.3±7.9	196.2±13.7
5	11	4	22.5	88.0±7.6	145.4±5.7	80.5±10.3	114.8±5.5
6	11	4	22.5	91.4±8.4	132.6±3.9	80.5±5.6	104.7±7.3
7	2	3	22.5	91.5±9.7	144.6±7.1	80.3±6.5	114.2±5.7
8	2	5	22.5	93.2±10.3	135.5±5.7	95.6±7.7	107.4±7.7
9	11	3	30	96.6±5.4	176.9±8.1	87.9±4.6	139.7±4.6
10	20	3	22.5	61.5±3.6	205.6±6.2	56.4±7.1	162.4±8.7
11	11	5	15	96.5±4.6	169.7±4.2	92.1±4.9	134.6±4.6
12	11	5	30	96.5±4.7	175.2±7.4	94.6±5.3	138.5±3.7
13	2	4	15	71.9±6.2	125.6±5.9	64.9±4.8	104.1±4.6
14	20	4	15	59.7±3.2	202.6±4.3	51.7±5.6	160.5±7.7
15	11	4	22.5	90.9±4.6	135.6±3.6	78.3±3.2	107.2±2.9
16	11	3	15	82.1±7.4	145.3±4.3	68.0±4.6	114.8±6.6
17	11	4	22.5	91.9±5.6	132.8±4.7	83.6±8.9	104.7±7.9

[a] calculated as particles/ particles measured by using nanoparticle tracking analysis (NTA).
[b] each cycle is composed of freezing at -80 °C followed by defrosting at room temperature.
[c] calculated by measuring fluorescence resonance energy transfer (FRET) dissolution efficiency.
[d] measured using dynamic light scattering technique (DLS).
[e] expressed as mean ± SD (n=3).

Table S4: Experimental Design Matrix of the CPPs and the Related CQAs of ELNs$_{BL6}$, ELNs$_{CT26}$ and ELNs$_{GL261}$ Prepared by Freeze-Thaw Method.

Run	A: LNP-Exosome ratio [a]	B: Number of cycles [b]	C: Duration (min)	ELNs$_{BL6}$		ELNs$_{CT26}$		ELNs$_{GL261}$	
				Fusion efficiency (%) [c, e]	Particle size (nm) [d, e]	Fusion efficiency (%) [c, e]	Particle size (nm) [d, e]	Fusion efficiency (%) [c, e]	Particle size (nm) [d, e]
1	2	4	30	78.3±3.3	115.2±9.6	56.8±2.3	158.5±6.2	84.3±5.2	110.7±5.6
2	20	4	30	52.1±3.7	168.9±3.3	36.5±1.6	241.5±15.3	54.3±3.1	181.7±10.1
3	11	4	22.5	75.7±2.5	102.3±9.3	55.2±4.3	139.3±4.1	79.6±5.4	105.2±6.5
4	20	5	22.5	66.5±4.5	185.2±4.9	50.2±5.9	249.6±11.3	83.2±2.6	186.4±4.5
5	11	4	22.5	74.7±4.7	100.5±8.9	56.9±7.5	144.9±9.0	84.1±8.1	109.5±4.7
6	11	4	22.5	78.9±4.5	105.6±10.6	56.3±4.5	135.5±4.3	84.9±4.3	109.5±6.3
7	2	3	22.5	70.2±1.4	108.5±7.7	55.7±5.8	148.5±4.6	85.6±6.3	115.3±4.7
8	2	5	22.5	84.3±4.7	116.2±6.9	64.5±7.9	143.6±4.2	97.2±1.3	100.2±8.2
9	11	3	30	81.1±4.4	121.2±4.9	59.7±4.3	177.4±7.6	85.3±3.6	135.6±4.1
10	20	3	22.5	57.9±2.1	154.4±4.9	38.7±5.7	210.2±8.1	57.4±5.1	157.9±6.3
11	11	5	15	80.5±5.6	127.2±7.7	65.1±5.3	186.7±4.5	94.0±3.2	124.5±4.6
12	11	5	30	90.6±8.3	131.5±6.3	49.1±4.7	186.6±5.4	95.5±2.5	136.5±4.8
13	2	4	15	61.5±4.2	114.6±5.8	47.3±3.6	135.7±3.2	66.8±5.6	108.3±6.9
14	20	4	15	51.2±3.6	156.2±9.9	35.1±4.6	225.2±5.4	55.8±4.1	151.6±9.7
15	11	4	22.5	77.9±4.5	103.8±9.1	50.3±6.4	147.5±4.5	87.4±7.9	105.6±4.6
16	11	3	15	70.4±4.7	106.6±8.3	37.9±7.5	145.2±4.3	71.5±2.3	104.6±4.9
17	11	4	22.5	74.3±1.9	110.2±5.4	54.8±5.04	127.5±3.2	85.6±4.9	109.7±6.5

[a] calculated as particles/ particles measured by using nanoparticle tracking analysis (NTA).
[b] each cycle is composed of freezing at -80 °C followed by defrosting at room temperature.
[c] calculated by measuring fluorescence resonance energy transfer (FRET) dissolution efficiency.
[d] measured using dynamic light scattering technique (DLS).
[e] expressed as mean ± SD (n=3).

Table S5: Experimental Design Matrix of the CPPs and the Related CQAs of $ELNs_{4T1}$ and $ELNs_{B16F10}$ Prepared by Sonication Method.

Run	A: LNP-Exosome ratio [a]	B: Number of cycles [b]	C: Duration (Sec)	$ELNs_{4T1}$		$ELNs_{B16F10}$	
				Fusion efficiency (%) [c, e]	Particle size (nm) [d, e]	Fusion efficiency (%) [c, e]	Particle size (nm) [d, e]
1	20	3	45	35.1±3.3	166.5±3.2	40.4±3.2	110.7±2.3
2	11	3	60	51.4±4.1	247.6±5.2	58.9±5.2	164.7±5.5
3	11	3	30	41.5±5.3	148.8±4.1	47.6±3.3	99.2±3.3
4	2	4	60	76.4±3.4	258.4±6.3	87.8±4.5	172.2±4.7
5	11	4	45	51.9±6.2	168.6±4.3	59.6±3.2	112.3±6.3
6	11	4	45	53.1±5.5	149.3±6.5	61.9±4.6	99.5±5.5
7	11	4	45	49.2±1.2	162.9±8.9	56.5±3.2	108.6±10.3
8	20	4	60	37.5±6.2	152.9±10.3	43.1±2.4	101.9±6.3
9	2	3	45	69.4±2.3	199.1±3.2	79.8±4.5	132.7±4.6
10	11	5	30	47.1±3.1	239.2±4.7	54.1±4.4	159.3±6.4
11	20	5	45	39.3±5.3	198.9±6.4	45.2±3.2	132.5±3.5
12	11	5	60	56.9±4.2	203.9±7.7	65.4±4.6	135.9±4.7
13	11	4	45	49.9±5.6	141.3±10.3	57.5±4.2	94.2±10.3
14	2	5	45	76.9±6.3	228.9±3.7	88.4±3.2	152.6±6.4
15	11	4	45	51.7±7.5	152.9±5.7	59.8±4.6	101.9±4.6
16	20	4	30	33.4±5.7	180.7±4.7	38.4±3.2	120.4±3.4
17	2	4	30	61.9±6.3	147.9±3.7	71.3±6.2	98.6±5.7

[a] calculated as particles/ particles measured by using nanoparticle tracking analysis (NTA).
[b] each cycle is composed of sonication at 60 °C followed by incubation in ice.
[c] calculated by measuring fluorescence resonance energy transfer (FRET) dissolution efficiency.
[d] measured using dynamic light scattering technique (DLS).
[e] expressed as mean ± SD (n=3).

Table S6: Experimental Design Matrix of the CPPs and the Related CQAs of $ELNs_{BL6}$, $ELNs_{CT26}$ and $ELNs_{GL261}$ Prepared by Sonication Method.

Run	A: LNP-Exosome ratio [a]	B: Number of cycles [b]	C: Duration (Sec)	$ELNs_{BL6}$		$ELNs_{CT26}$		$ELNs_{GL261}$	
				Fusion efficiency (%) [c, e]	Particle size (nm) [d, e]	Fusion efficiency (%) [c, e]	Particle size (nm) [d, e]	Fusion efficiency (%) [c, e]	Particle size (nm) [d, e]
1	20	3	45	33.2±3.7	141.5±4.3	29.6±2.3	155.2±3.7	43.9±3.1	111.5±2.2
2	11	3	60	43.5±5.3	210.5±3.3	41.5±3.6	226.2±3.3	64.9±1.1	165.8±3.2
3	11	3	30	34.4±5.7	126.4±4.1	43.6±4.5	133.5±4.6	51.6±2.3	99.5±1.3
4	2	4	60	63.5±2.3	219.6±3.7	75.6±9.3	245.6±3.6	95.7±4.1	176.8±6.4
5	11	4	45	45.6±4.7	143.3±2.3	48.8±2.3	152.3±4.5	64.8±3.2	112.5±11.2
6	11	4	45	44.2±4.2	126.9±4.4	50.8±1.4	146.5±6.3	66.3±1.3	100.3±3.3
7	11	4	45	40.8±1.2	138.4±2.2	46.4±6.3	154.3±4.1	65.2±3.2	106.5±6.7
8	20	4	60	31.2±3.3	129.9±3.4	40.3±4.7	142.5±2.5	46.7±1.4	102.4±7.6
9	2	3	45	57.6±5.2	169.2±4.6	65.9±5.7	185.6±4.6	86.4±4.3	133.6±6.3
10	11	5	30	42.7±4.3	203.3±3.2	48.5±5.7	201.3±12.3	58.9±4.1	160.5±4.6
11	20	5	45	32.7±4.7	169.7±2.4	37.6±3.4	179.5±5.3	49.6±3.2	133.4±7.7
12	11	5	60	47.2±5.3	173.3±4.7	55.7±7.3	190.2±4.6	75.7±4.1	136.7±3.3
13	11	4	45	41.5±3.2	120.1±2.3	48.4±6.4	151.2±3.3	62.4±2.3	94.6±4.6
14	2	5	45	63.9±2.1	194.5±2.4	75.7±8.9	213.5±4.9	96.5±3.3	153.6±7.3
15	11	4	45	44.7±1.4	129.9±4.2	45.7±6.6	146.5±11.3	64.6±4.7	102.5±6.3
16	20	4	30	31.3±3.3	153.4±3.3	37.5±5.7	165.3±10.3	41.6±3.3	124.7±4.6
17	2	4	30	51.3±4.3	125.7±1.3	71.3±8.3	141.2±4.5	77.4±4.1	99.5±3.2

[a] calculated as particles/ particles measured by using nanoparticle tracking analysis (NTA).
[b] each cycle is composed of sonication at 60 °C followed by incubation in ice.
[c] calculated by measuring fluorescence resonance energy transfer (FRET) dissolution efficiency.
[d] measured using dynamic light scattering technique (DLS).
[e] expressed as mean ± SD (n=3).

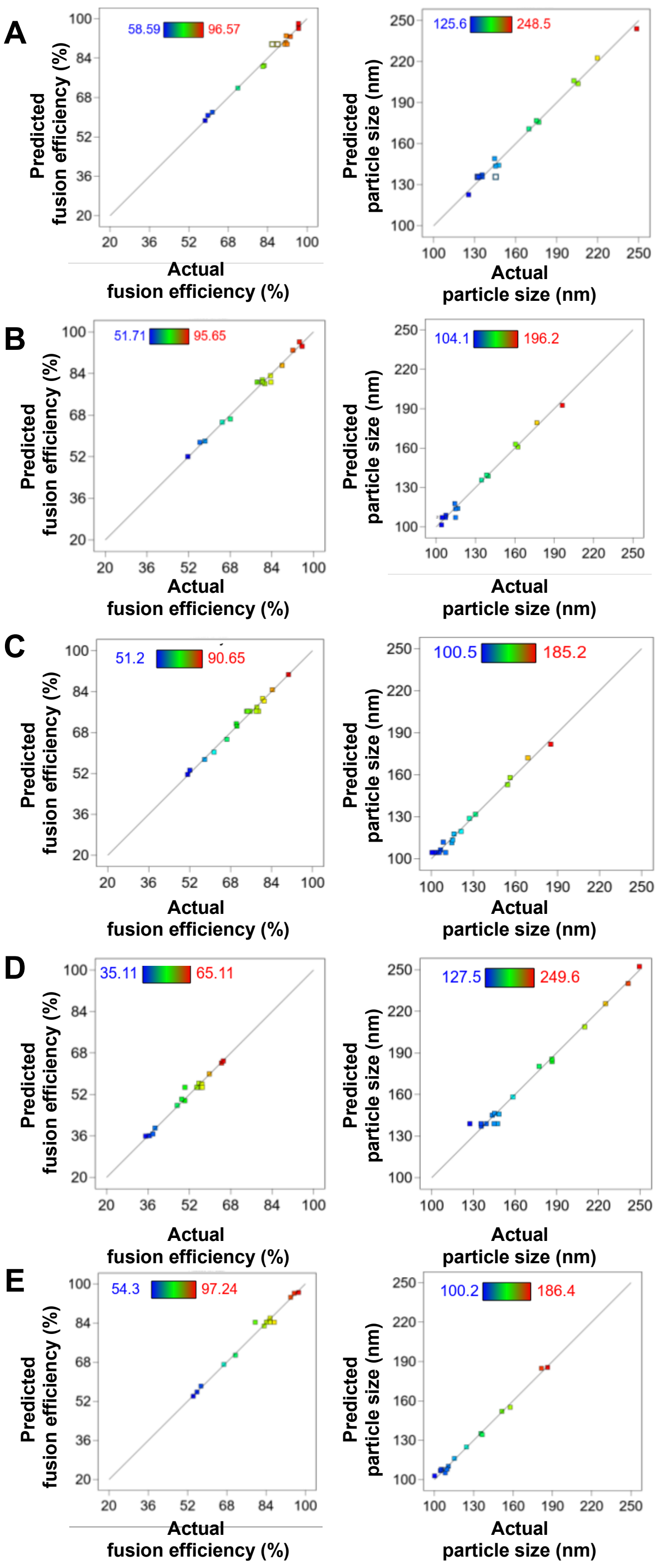

Figure S3: Correlation between actual and predicted values for fusion efficiency and particle size of different ELNs prepared by freeze-thaw technique. Scatter plots show the relationship between experimentally observed (actual) and model-predicted values for fusion efficiency and particle size for **(A)** FT-ELNs$_{4T1}$, **(B)** FT-ELNs$_{B16F10}$, **(C)** FT-ELNs$_{BL6}$, **(D)** FT-ELNs$_{CT26}$, **(E)** FT-ELNs$_{GL261}$. The strong linear correlation and clustering of data points around the identity line (y = x) indicate goodness of fit and predictive reliability of the response surface models generated using BBD.

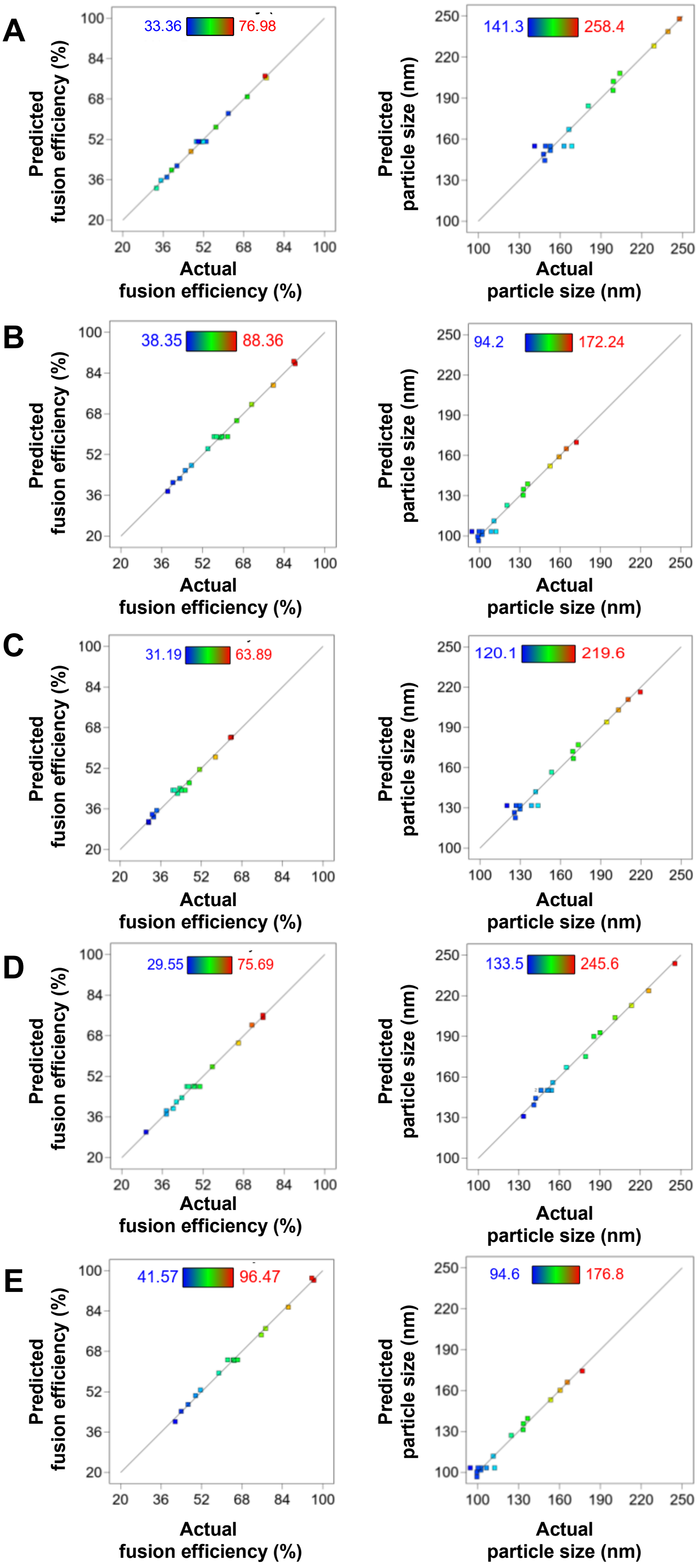

Figure S4: Actual versus predicted plots for fusion efficiency and particle size are shown for each sonication-based ELNs formulation. Scatter plots show the relationship between experimentally observed (actual) and model-predicted values for fusion efficiency and particle size for **(A**) FT-ELNs$_{4T1}$, **(B)** FT-ELNs$_{B16F10}$, **(C)** FT-ELNs$_{BL6}$, **(D)** FT-ELNs$_{CT26}$, **(E)** FT-ELNs$_{GL261}$. The close alignment of data points with the identity line reflects the accuracy and robustness of the BBD model predictions, supporting the validity of the selected design parameters for optimizing ELNs fabrication.

Table S7: Model Summary Statistics for the Prepared ELNs.

ELNs type	Preparation method	Response	R^2	Adjusted R^2	Predicted R^2	PRESS [a]
$ELNs_{4T1}$	Freeze-thaw	Fusion efficiency (%)	0.9855	0.9669	0.9086	251.1
		Particle size (nm)	0.9897	0.9766	0.9203	1687.68
	Sonication	Fusion efficiency (%)	0.9955	0.9898	0.9795	57.37
		Particle size (nm)	0.9766	0.9465	0.9099	2159.24
$ELNs_{B16F10}$	Freeze-thaw	Fusion efficiency (%)	0.9905	0.9784	0.9226	216.43
		Particle size (nm)	0.9896	0.9761	0.9174	1094.61
	Sonication	Fusion efficiency (%)	0.9944	0.9872	0.9788	78.25
		Particle size (nm)	0.9766	0.9466	0.9091	965.46
$ELNs_{BL6}$	Freeze-thaw	Fusion efficiency (%)	0.9876	0.9718	0.9259	146.56
		Particle size (nm)	0.9891	0.9751	0.9017	1032.54
	Sonication	Fusion efficiency (%)	0.9858	0.9676	0.9235	130.77
		Particle size (nm)	0.9764	0.9462	0.9064	1623.26
$ELNs_{CT26}$	Freeze-thaw	Fusion efficiency (%)	0.9766	0.9465	0.9096	127.57
		Particle size (nm)	0.9882	0.9730	0.9573	1062.14
	Sonication	Fusion efficiency (%)	0.9930	0.9840	0.9677	97.44
		Particle size (nm)	0.9927	0.9833	0.9239	1336.13
$ELNs_{GL261}$	Freeze-thaw	Fusion efficiency (%)	0.9875	0.9714	0.9658	100.88
		Particle size (nm)	0.9944	0.9871	0.9351	783.24
	Sonication	Fusion efficiency (%)	0.9961	0.9911	0.9652	154.56
		Particle size (nm)	0.9806	0.9556	0.9208	889.3

[a] Predicted Residual Error Sum of Squares.

Table S8: The Equations of DoE Predicted Models.

ELNs type	Preparation method	Response	Equation
$ELNs_{4T1}$	Freeze-thaw	Fusion efficiency (%)	$Y1= +89.65 -10.70A +4.65B +4.10C +4.82AB -5.15AC -3.61BC -14.99A^2 +7.55B^2 -4.27C^2$
		Particle size (nm)	$Y2 = 135.74 +40.38A +7.06B +9.52C+13AB +27.45A^2 +20.35B^2 +10.68C^2$
	Sonication	Fusion efficiency (%)	$Y1= 51.15 -17.42A +2.89B +4.79C -2.62AC +3.59A^2 -2.46C^2$
		Particle size (nm)	$Y2=+155.00 -16.91A +13.61B +18.28C -34.57AC -33.52BC +34.12B^2 +20.75C^2$
$ELNs_{B16F10}$	Freeze-thaw	Fusion efficiency (%)	$Y1 = 80.68 -9.59A +8.87B +5.94C +2.42AB -2.97AC -4.35BC -11.66A^2 +9.40B^2 -4.40C^2$
		Particle size (nm)	$Y2 = 107.18 +31.7A +5.7B +7.2C +10.15AB +22.73A^2 +15.13B^2 +9.58C^2$
	Sonication	Fusion efficiency (%)	$Y1= +59.04 -20.02A +3.30B +5.48C -2.96AC +4.00A^2 -2.91C^2$
		Particle size (nm)	$Y2= 103.3 -11.32A +9.12B +12.14C -23.04AC -22.23BC +22.64B^2 +13.81C^2$
$ELNs_{BL6}$	Freeze-thaw	Fusion efficiency (%)	$Y1 = 76.31 -8.33A +5.29B +4.79C -3.96AC -13.25A^2 +6.66B^2 -2.30C^2$
		Particle size (nm)	$Y2 = 104.48 +26.27A +8.67B +4.02C +5.77AB +26.84A^2 +9.74B^2 +7.39C^2$
	Sonication	Fusion efficiency (%)	$Y1 = 43.36 -13.49A +2.21B +3.22C -3.05AC +2.91A^2$
		Particle size (nm)	$Y2= +131.72 -14.31A +11.65B +15.56C -29.35AC -28.52BC +29.12B^2 +17.54C^2$
$ELNs_{CT26}$	Freeze-thaw	Fusion efficiency (%)	$Y1 = 54.73 -7.95A +4.60B +2.10C -9.48BC -5.72A^2 +3.26B^2 -5.05C^2$
		Particle size (nm)	$Y2 = 138.94 +42.52A +10.65B +8.9C+11.07AB -8.07BC +32.64A^2 +16.39B^2 +18.64C^2$
	Sonication	Fusion efficiency (%)	$Y1 = 47.99 -17.94A +4.59B +1.50C +2.32BC +6.52A^2 -2.31B^2$
		Particle size (nm)	$Y2 = 150.16 -17.92A + 10.5B + 20.4C -31.8AC -25.95BC +9.57A^2 +23.72B^2 +13.92C^2$
$ELNs_{GL261}$	Freeze-thaw	Fusion efficiency (%)	$Y1 = 84.32 -10.39A +8.76B +3.91C +3.52AB -4.77AC -3.08BC -12.36A^2 +8.91B^2 -6.64C^2$
		Particle size (nm)	$Y2 = 107.9 +30.38A +4.27B +9.43C +10.9AB +6.92AC -4.75BC +22.41A^2 +9.63B^2 +7.76C^2$
	Sonication	Fusion efficiency (%)	$Y1 = 64.64 -21.75A +4.21B +6.69C -3.295AC +3.50A^2 -2.83C^2$
		Particle size (nm)	$Y2 = 103.28 -11.43A +9.22B +12.18C -24.9AC -22.52BC +7.48A^2 +22.26B^2 +15.08C^2$

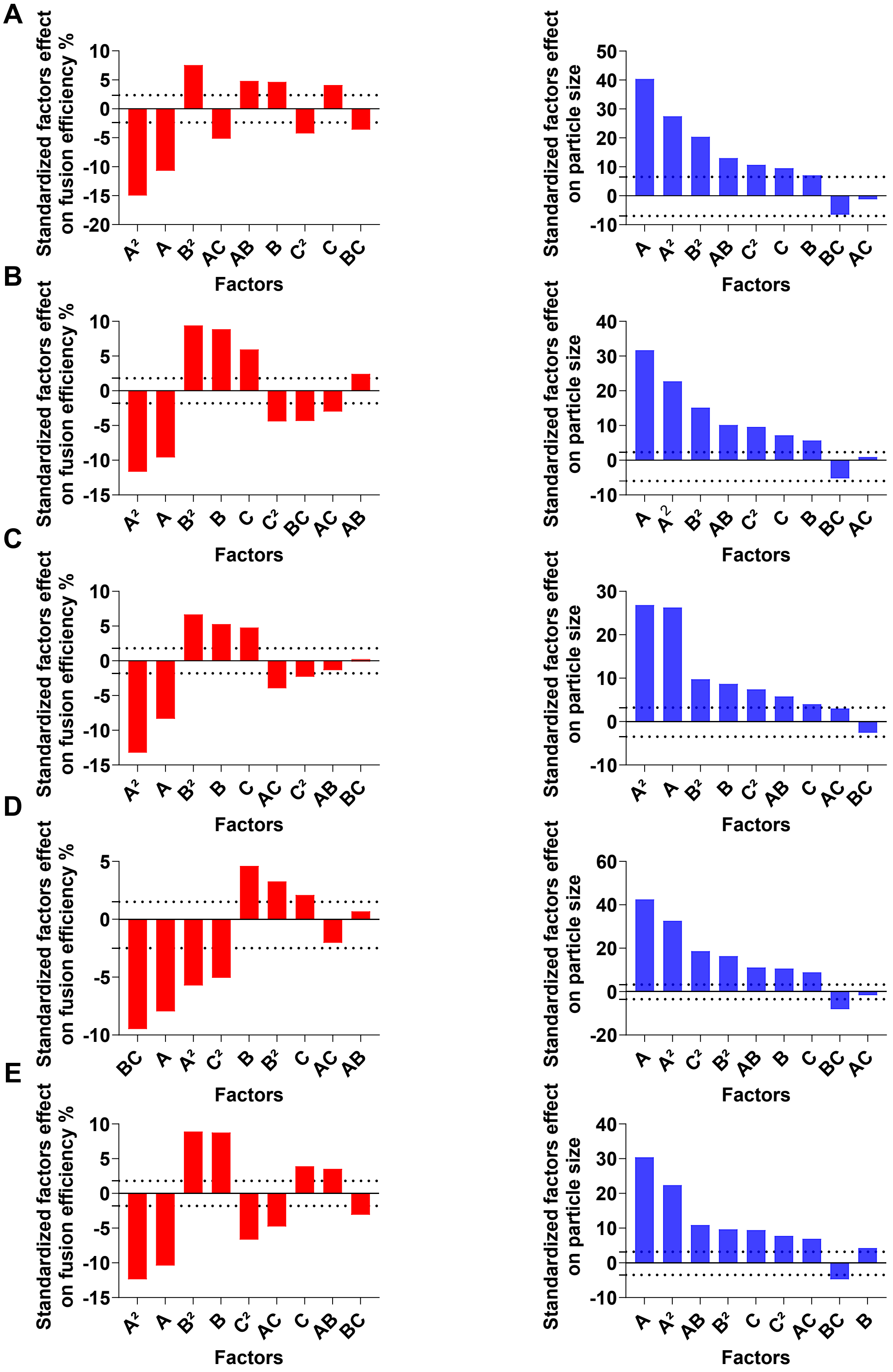

Figure S5: Pareto charts illustrating the impact of critical process parameters and their interactions on ELNs fusion efficiency and particle size prepared by freeze-thaw method. Pareto charts for ELNs derived from **(A)** 4T1, **(B)** B16F10, **(C)** BL6, **(D)** CT26 and **(E)** GL261 based exosomes. Bars represent the standardized effects of LNP-exosome ratio (A), number of cycles (B), and duration (C), as well as their interactions (AB, AC, BC), on fusion efficiency and particle size. The black dashed line indicates the threshold for statistical significance ($p < 0.05$). Positive effects (bars above zero) denote an increase in the response variable, while negative effects (bars below zero) indicate a decrease.

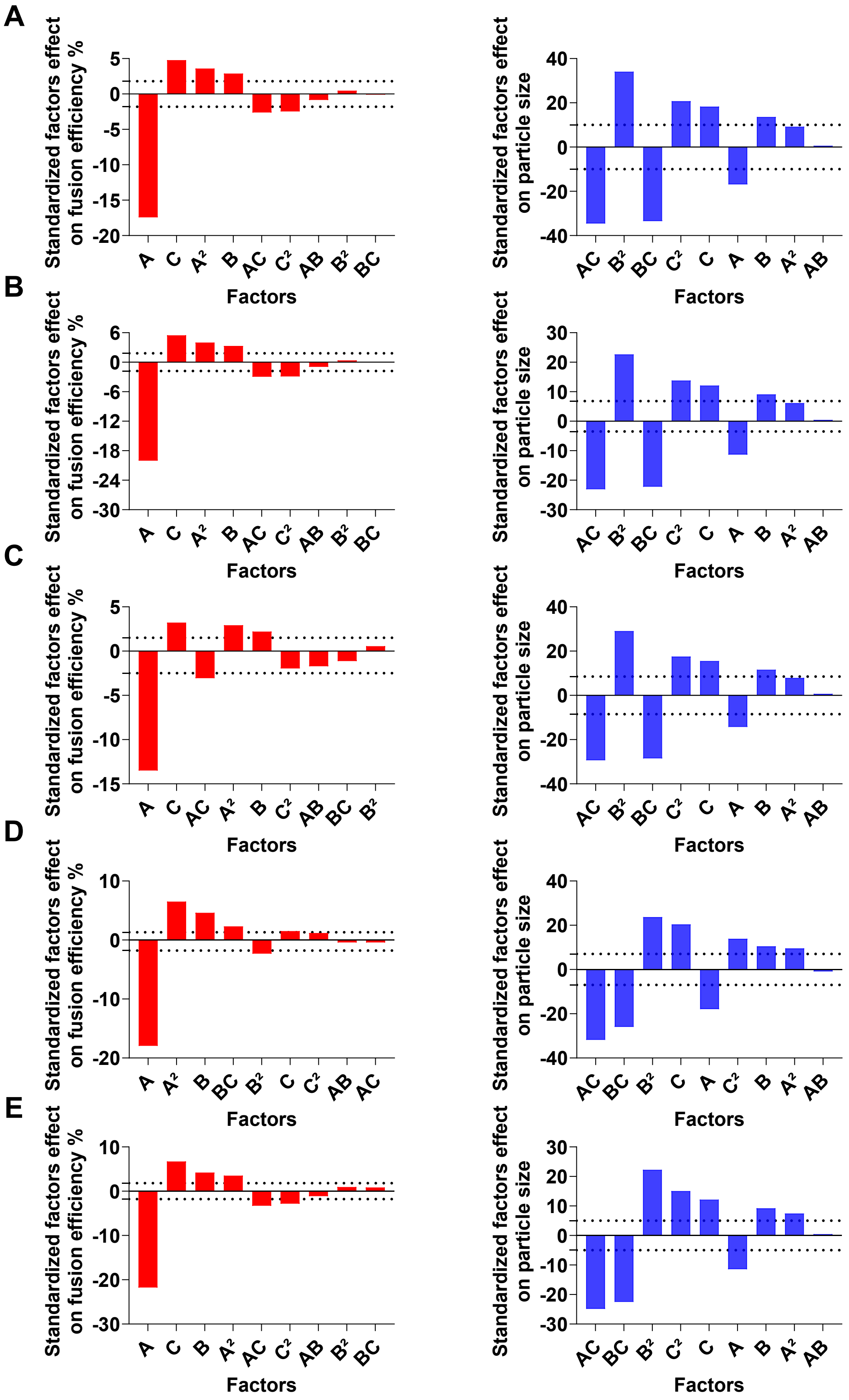

Figure S6: Pareto charts depicting the influence of key process parameters and their interactions on ELNs fusion efficiency and particle size prepared by sonication method. Pareto charts for ELNs derived from **(A)** 4T1, **(B)** B16F10, **(C)** BL6, **(D)** CT26 and **(E)** GL261 based exosomes. The bars reflect the standardized effects of LNP-exosome ratio (A), number of cycles (B) and duration (C), along with their interactions (AB, AC, BC), on fusion efficiency and particle size. The dashed black line marks the significance threshold ($p < 0.05$). Positive effects (bars above zero) correlate with increased response values, whereas negative effects (bars below zero) correspond to reductions.

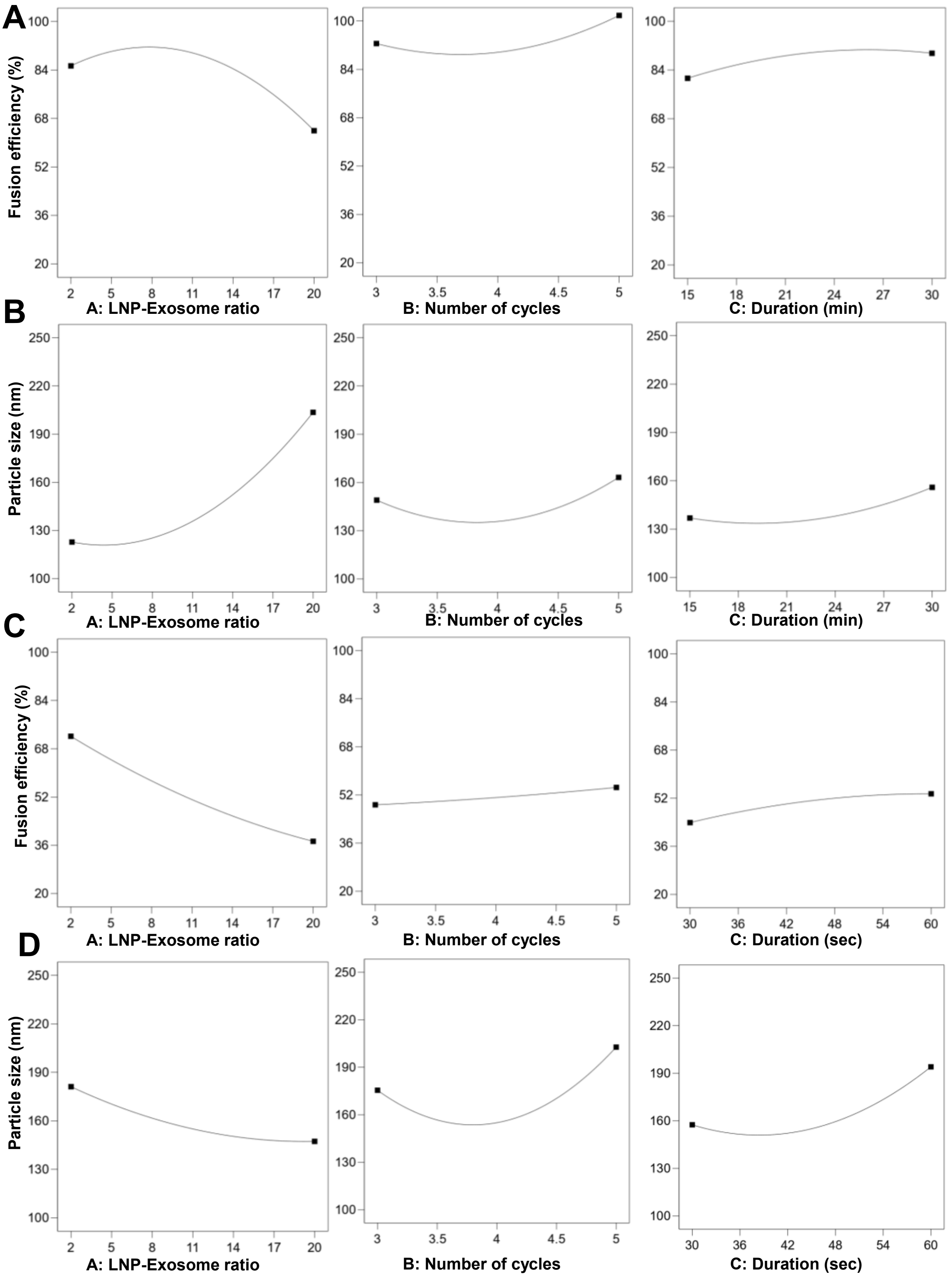

Figure S7: Plots of the main effect of different significant critical process parameters on ELNs$_{4T1}$. (A, B) Fusion efficiency and particle size of FT-ELNs$_{4T1}$ prepared by freeze-thaw method, **(C, D)** fusion efficiency and particle size of S-ELNs$_{4T1}$ prepared by sonication method. LNP-Exosome ratio had a negative influence on fusion efficiency in both hybrids and particle size of hybrids prepared by sonication only. Increasing LNP-Exosome ratio increased particle size of FT-ELNs$_{4T1}$ prepared by freeze-thaw method. Number of cycles and duration increased fusion efficiency and particle size of both ELNs.

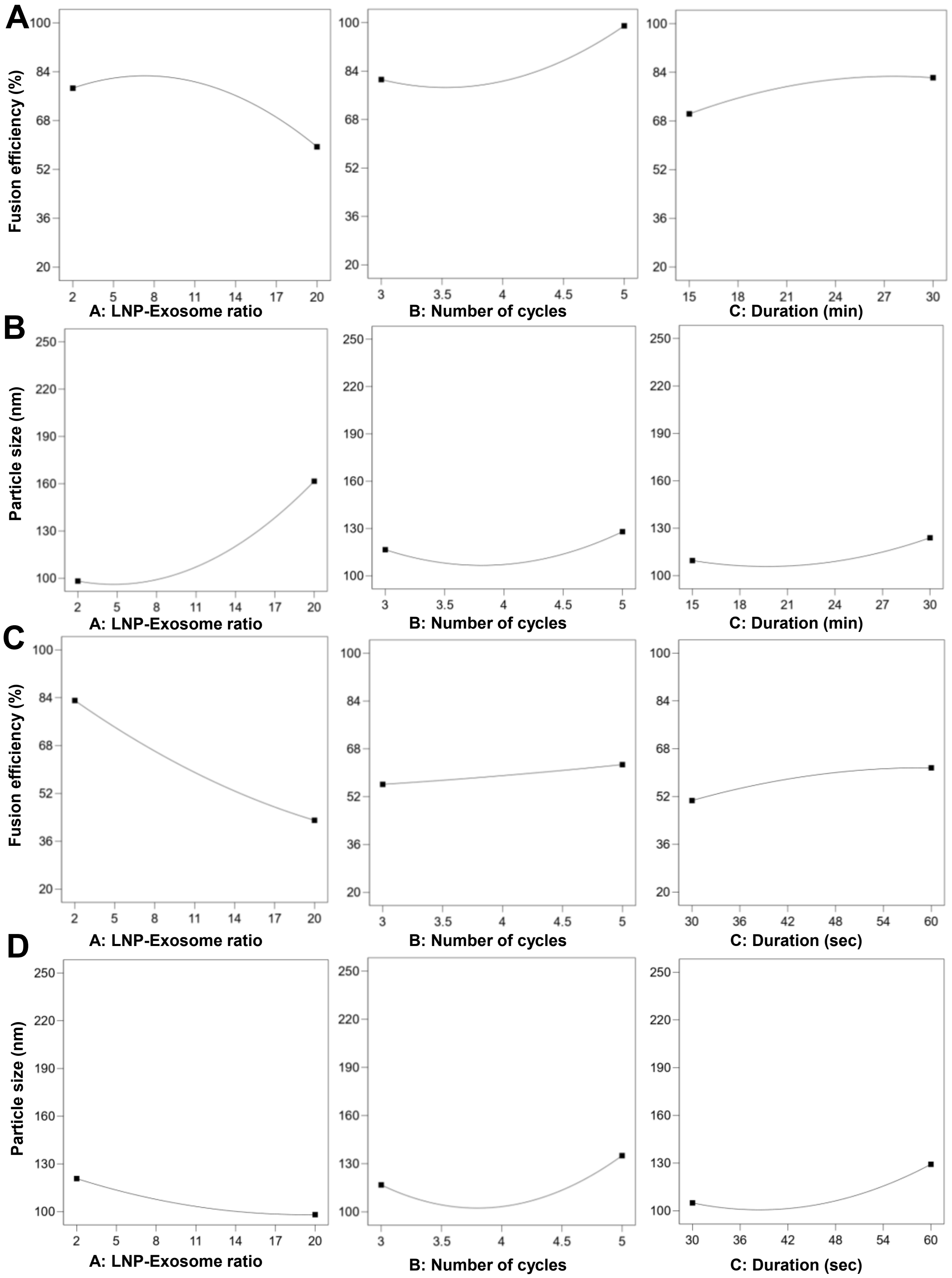

Figure S8: Plots of the main effect of different significant critical process parameters on ELNs$_{B16F10}$. (A, B) Fusion efficiency and particle size of FT-ELNs$_{B16F10}$ prepared by freeze-thaw method, **(C, D)** fusion efficiency and particle size of S-ELNs$_{B16F10}$ prepared by sonication method. LNP-Exosome ratio had a negative influence on fusion efficiency in both hybrids and particle size of hybrids prepared by sonication only. Increasing LNP-Exosome ratio increased particle size of FT-ELNs$_{B16F10}$ prepared by freeze-thaw method. Number of cycles and duration increased fusion efficiency and particle size of both ELNs.

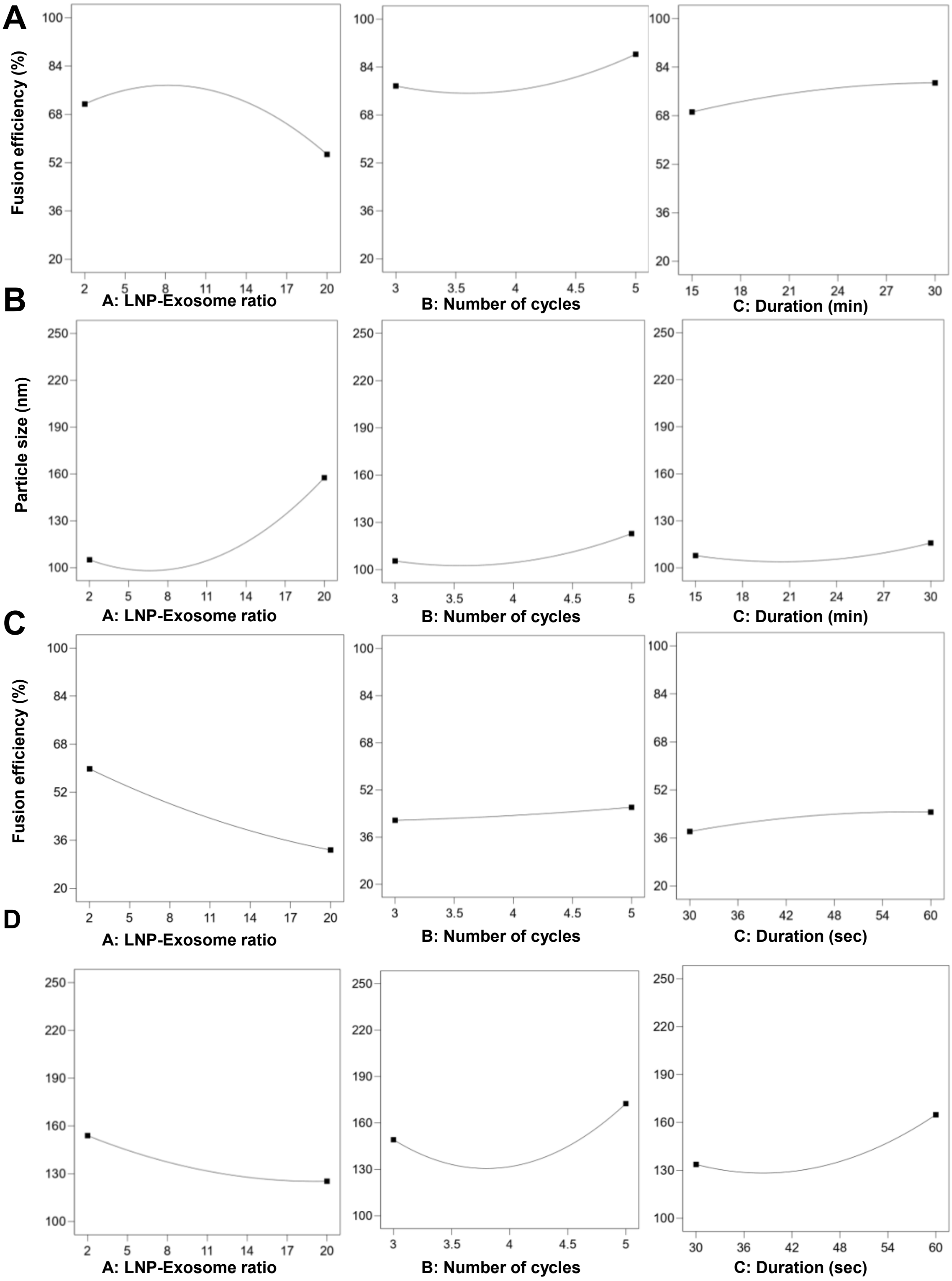

Figure S9: Plots of the main effect of different significant critical process parameters on ELNs$_{BL6}$. (A, B) Fusion efficiency and particle size of FT-ELNs$_{BL6}$ prepared by freeze-thaw method, **(C, D)** fusion efficiency and particle size of S-ELNs$_{BL6}$ prepared by sonication method. LNP-Exosome ratio had a negative influence on fusion efficiency in both hybrids and particle size of hybrids prepared by sonication only. Increasing LNP-Exosome ratio increased particle size of FT-ELNs$_{BL6}$ prepared by freeze-thaw method. Number of cycles and duration increased fusion efficiency and particle size of both ELNs.

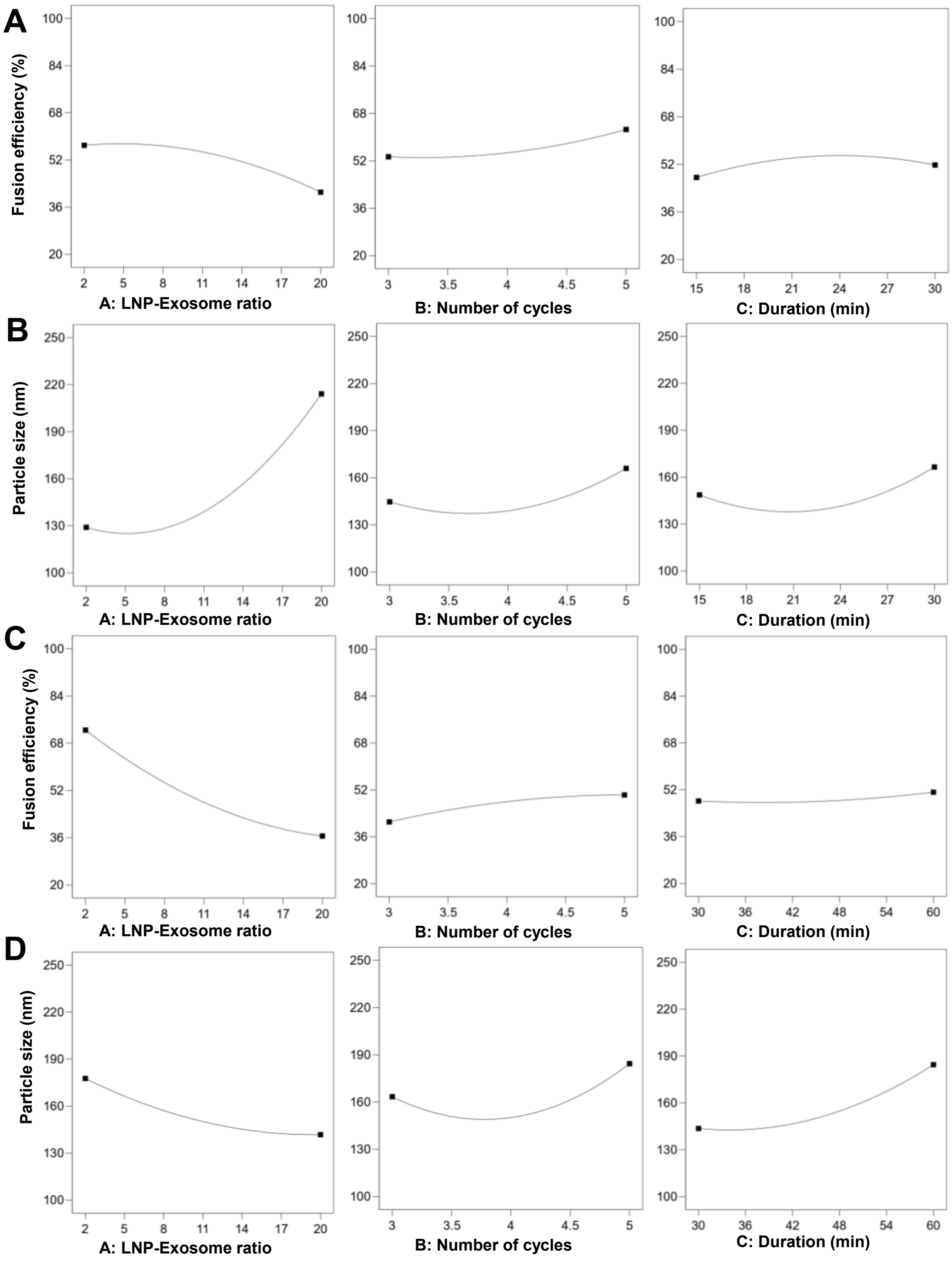

Figure S10: Plots of the main effect of different significant critical process parameters on ELNs$_{CT26}$. (A, B) Fusion efficiency and particle size of FT-ELNs$_{CT26}$ prepared by freeze-thaw method, **(C, D)** fusion efficiency and particle size of S-ELNs$_{CT26}$ prepared by sonication method. LNP-Exosome ratio had a negative influence on fusion efficiency in both hybrids and particle size of hybrids prepared by sonication only. Increasing LNP-Exosome ratio increased particle size of FT-ELNs$_{CT26}$ prepared by freeze-thaw method. Number of cycles and duration increased fusion efficiency and particle size of both ELNs.

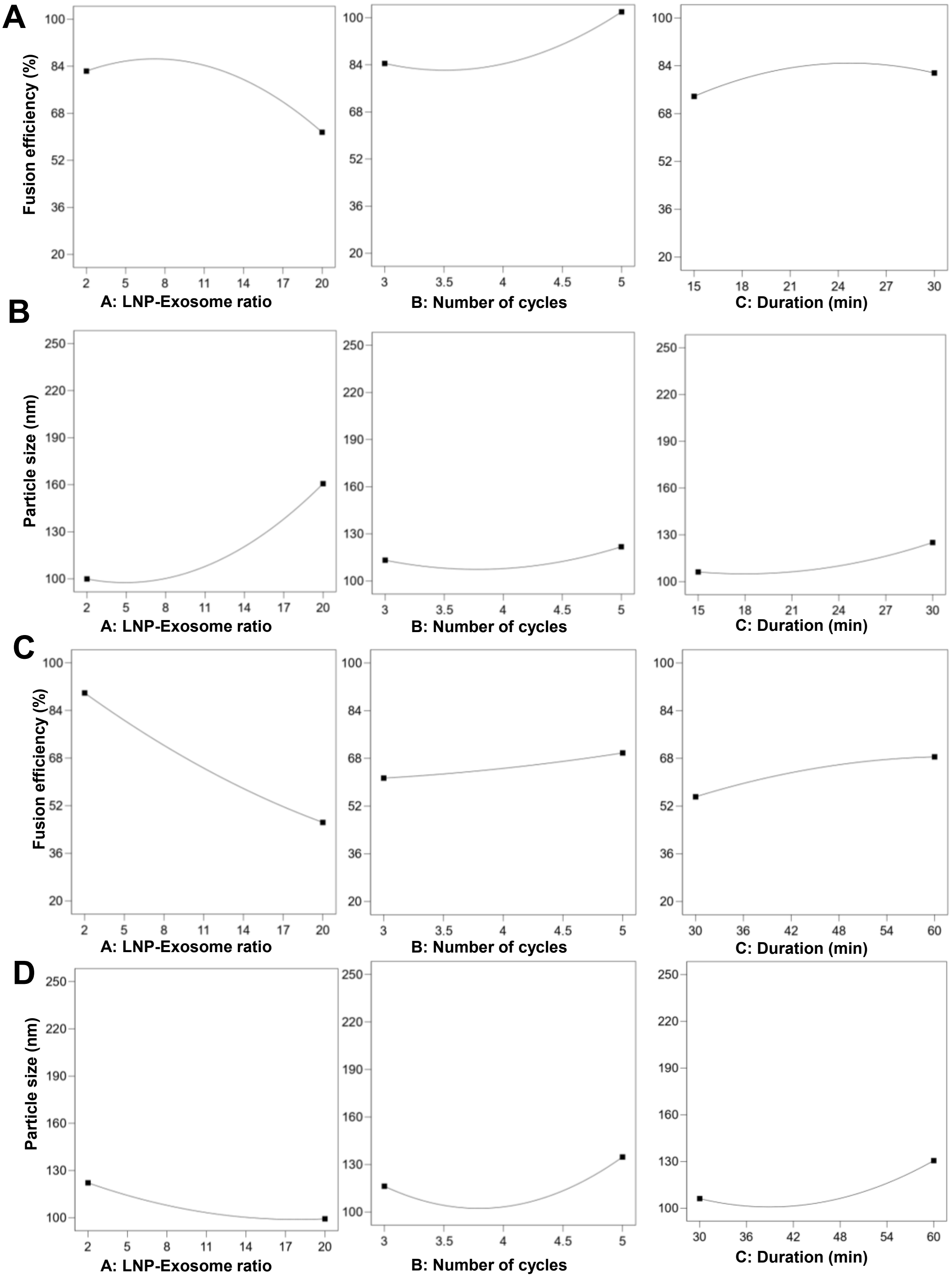

Figure S11: Plots of the main effect of different significant critical process parameters on ELNs$_{GL261}$. (A, B) Fusion efficiency and particle size of FT-ELNs$_{GL261}$ prepared by freeze-thaw method, **(C, D)** fusion efficiency and particle size of S-ELNs$_{GL261}$ prepared by sonication method. LNP-Exosome ratio had a negative influence on fusion efficiency in both hybrids and particle size of hybrids prepared by sonication only. Increasing LNP-Exosome ratio increased particle size of FT-ELNs$_{GL261}$ prepared by freeze-thaw method. Number of cycles and duration increased fusion efficiency and particle size of both ELNs.

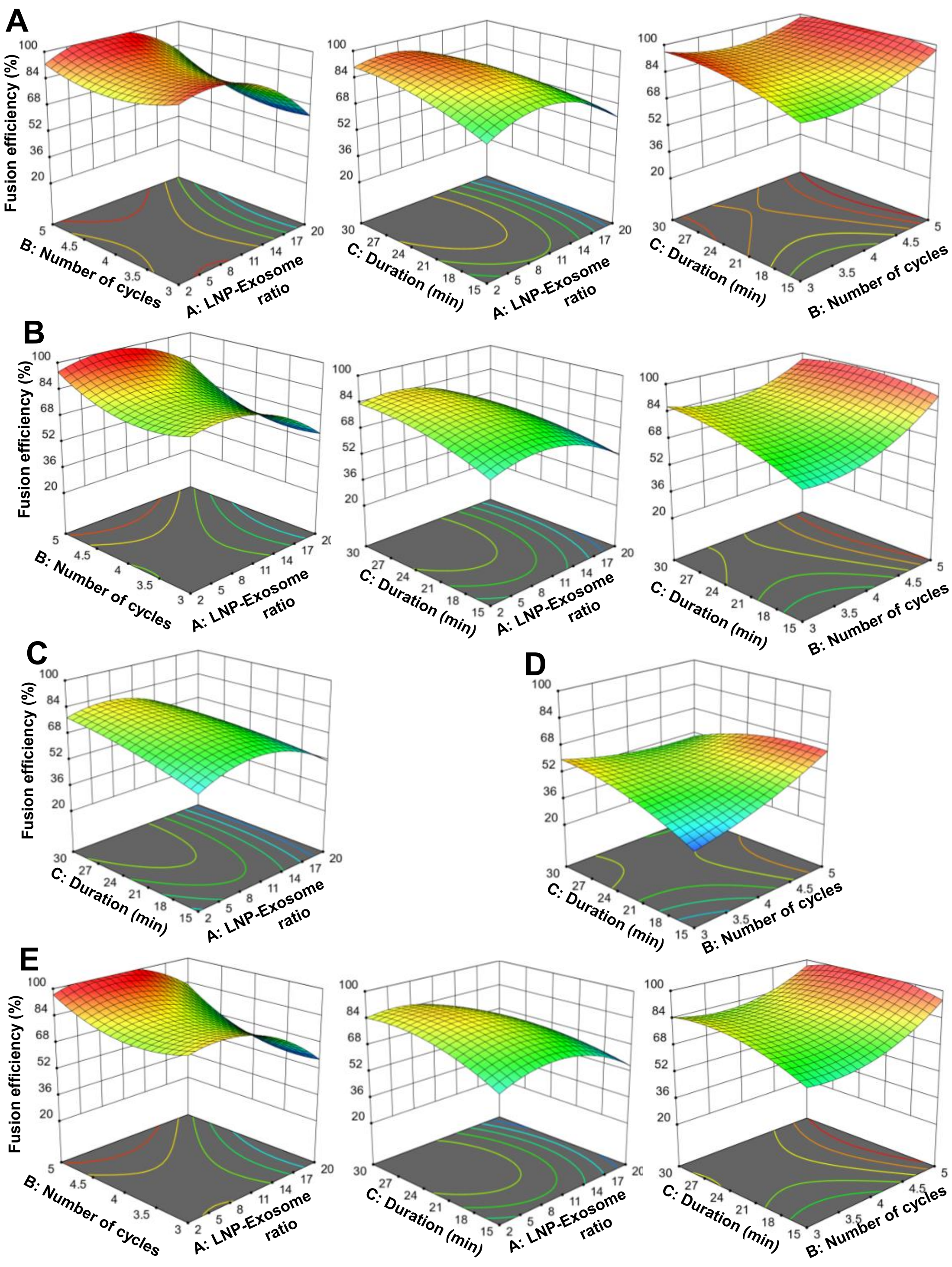

Figure S12: Response 3D plots for the significant parameters' interaction on fusion efficiency of different ELNs prepared by freeze-thaw technique. (A) FT-ELNs$_{4T1}$, **(B)** FT-ELNs$_{B16F10}$, **(C)** FT-ELNs$_{BL6}$, **(D)** FT-ELNs$_{CT26}$, (**E)** FT-ELNs$_{GL261}$. The interaction of LNP-Exosome ratio and number of cycles (AB) increased fusion efficiency of FT-ELNs$_{4T1}$, FT-ELNs$_{B16F10}$ and FT-ELNs$_{GL261}$. The interaction of LNP-Exosome ratio and duration (AC) and of number of cycles and duration (BC) had a negative influence on fusion efficiency of FT-ELNs$_{4T1}$, FT-ELNs$_{B16F10}$ and FT-ELNs$_{GL261}$. FT-ELNs$_{BL6}$ fusion efficiency is inversely proportional to the interaction of LNP-Exosome ratio and time (AC) only. The interaction of number of cycles and time (BC) is the only significant parameter interaction that negatively affects FT-ELNs$_{CT26}$.

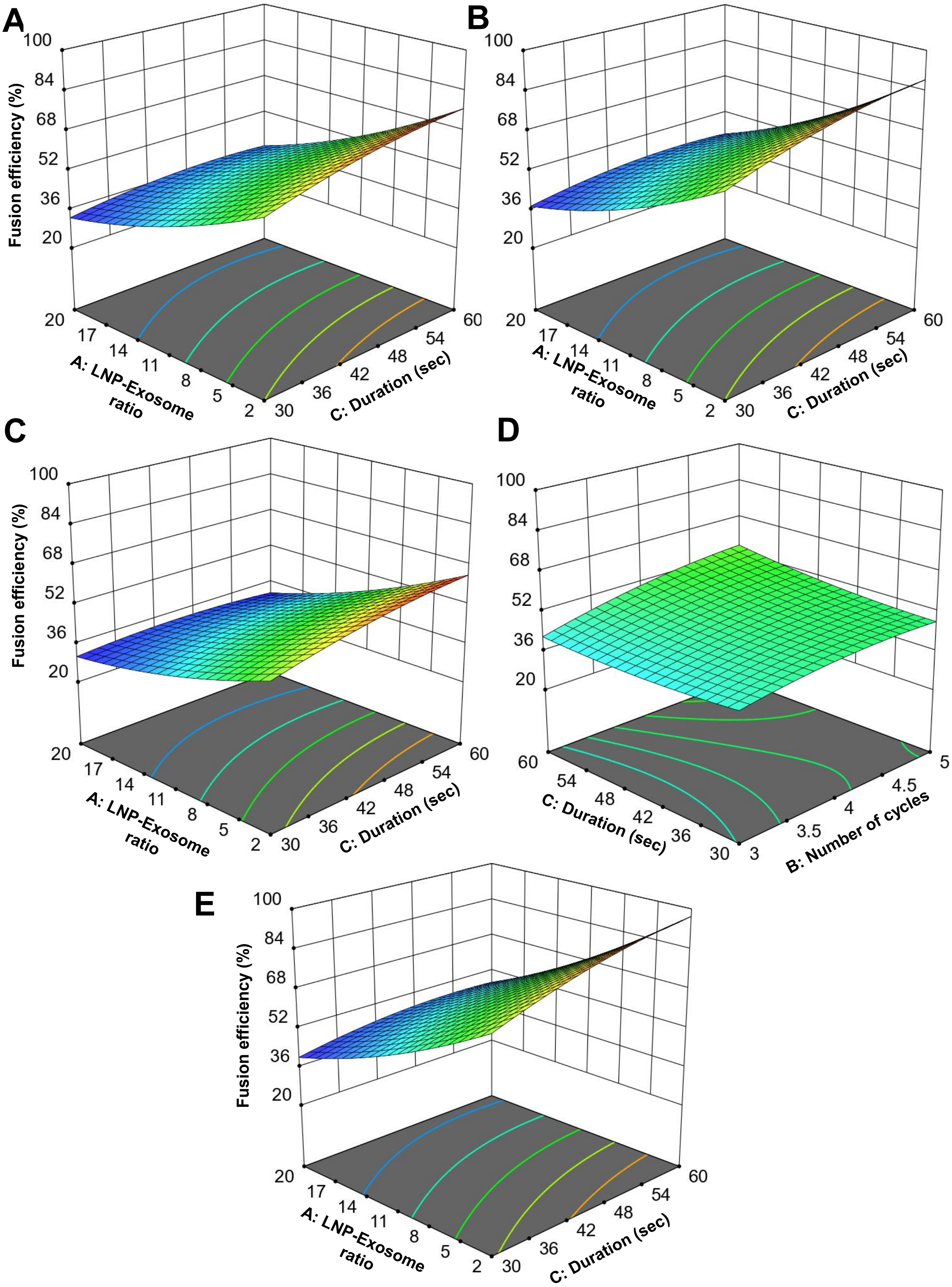

Figure S13: Response 3D plot for the significant parameters' interaction on fusion efficiency of different ELNs prepared by sonication method. (A) S-ELNs$_{4T1}$, **(B)** S-ELNs$_{B16F10}$, **(C)** S-ELNs$_{BL6}$, **(D)** S-ELNs$_{CT26}$, **(E)** S-ELNs$_{GL261}$. The interaction of LNP-Exosome ratio and **duration** (AC) had a negative influence on fusion efficiency of S-ELNs$_{4T1}$, S-ELNs$_{B16F10}$, S-ELNs$_{BL6}$, S-ELNs$_{GL261}$. The interaction of number of cycles and duration (BC) is the only significant interaction that positively increase fusion efficiency of S-ELNs$_{CT26}$.

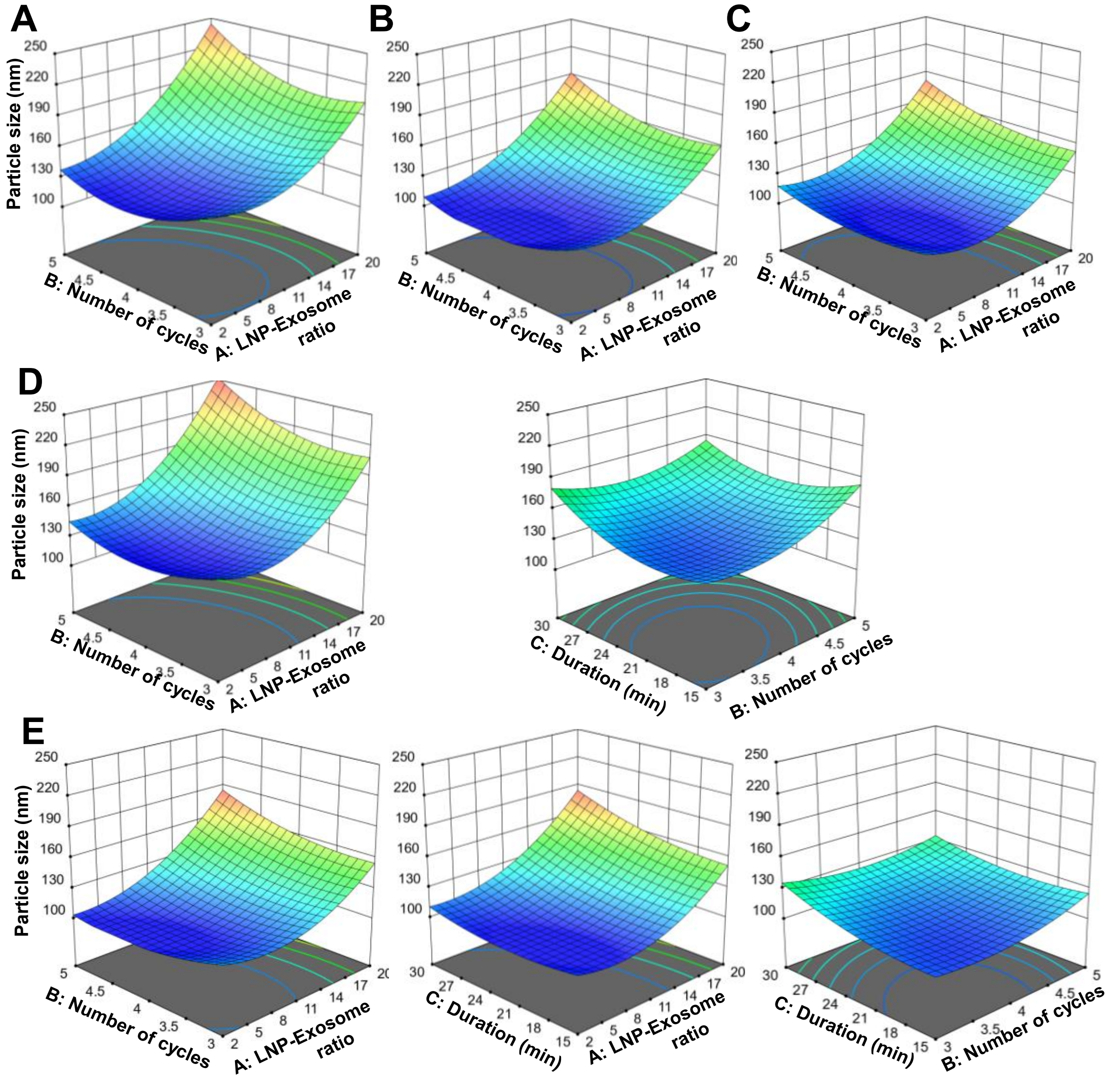

Figure S14: Response 3D plots for the significant parameters' interaction on particle size of different ELNs prepared by freeze-thaw technique. (A) FT-ELNs$_{4T1}$, **(B)** FT-ELNs$_{B16F10}$, **(C)** FT-ELNs$_{BL6}$, **(D)** FT-ELNs$_{CT26}$, **(E)** FT-ELNs$_{GL261}$. The interaction of LNP-Exosome ratio and number of cycles (AB) increased particle size across all preparations. The interaction of number of cycles and duration (BC) had a negative influence on particle size of FT-ELNs$_{CT26}$ and FT-ELNs$_{GL261}$. FT-ELNs$_{GL261}$ particle size is directly proportional with the interaction of LNP-Exosome ratio and duration (AC).

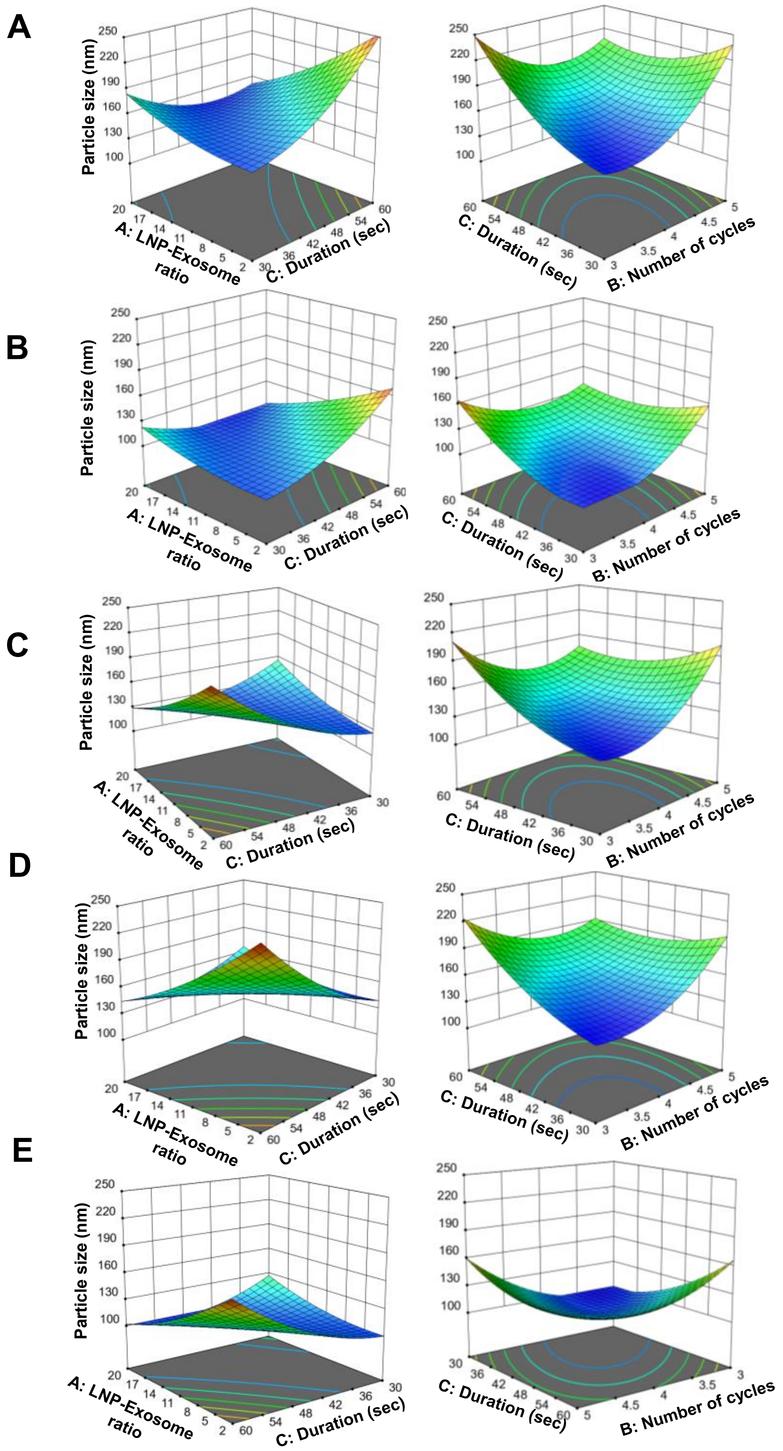

Figure S15: Response 3D plot for the significant parameters' interaction on particle size of different ELNs prepared by sonication method. (A) S-ELNs$_{4T1}$, **(B)** S-ELNs$_{B16F10}$, **(C)** S-ELNs$_{BL6}$, **(D)** S-ELNs$_{CT26}$, **(E)** S-ELNs$_{GL261}$. The interaction of LNP-Exosome ratio and duration (AC) and of number of cycles and duration (BC) had a negative influence on particle size of S-ELNs$_{4T1}$, S-ELNs$_{B16F10}$, S-ELNs$_{BL6}$, S-ELNs$_{CT26,}$ S-ELNs$_{GL261}$.

Table S9: Experimental and Predicted Fusion Efficiency and Particle Size of the Optimized ELNs Prepared by Freeze-Thaw Method.

Exosome source	LNP-Exosome ratio	Number of cycles	Duration (min)	Fusion efficiency (%)			Particle size (nm)		
				Pred.	Exp. [a, c]	% Pred. error	Pred.	Exp. [b, c]	% Pred. error
4T1	2	4	30	90.4	91.7± 4.8	1.4	144.2	148.0±5.5	2.6
B16F10				83.1	85.3± 5.1	2.6	114.1	120.8±6.9	5.5
BL6				77.9	82.1± 6.4	5.1	113.5	119.9±6.7	5.3
CT26				57.4	61.1±5.3	6.1	130.7	136.9±9.5	4.5
GL261				84.4	87.3± 3.3	3.3	110.2	119.3±6.5	7.6

[a] calculated by measuring fluorescence resonance energy transfer (FRET) dissolution efficiency.
[b] measured using dynamic light scattering technique (DLS).
[c] expressed as mean ± SD (n=3).

Table S10: Experimental and Predicted Fusion Efficiency and Particle Size of the Optimized ELNs Prepared by Sonication Method.

Exosome source	LNP-Exosome ratio	Number of cycles	Duration (sec)	Fusion efficiency (%)			Particle size (nm)		
				Pred.	Exp. [a, c]	% Pred. error	Pred.	Exp. [b, c]	% Pred. error
4T1	2	4	30	63.2	68.8±2.1	8.1	155.1	166.3±3.5	6.7
B16F10				71.6	77.0±5.2	7.0	97.0	114.6±9.5	15.4
BL6				50.6	53.9±1.4	6.1	120.1	129.3±5.5	7.1
CT26				72.2	73.0±8.2	1.1	139.3	145.2±6.2	4.1
GL261				76.2	78.9±2.3	3.4	94.6	105.6±3.2	10.4

[a] calculated by measuring fluorescence resonance energy transfer (FRET) dissolution efficiency.
[b] measured using dynamic light scattering technique (DLS).
[c] expressed as mean ± SD (n=3).

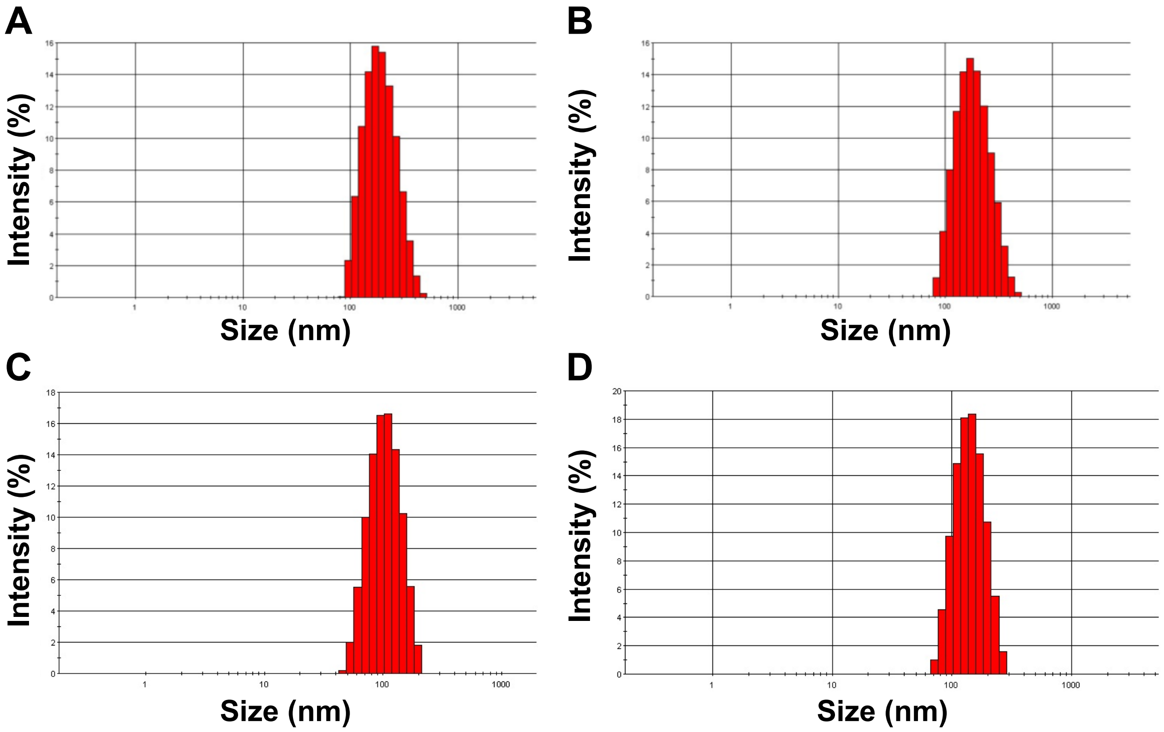

Figure S16: *In vitro* characterization of the optimized FT-ELNs. (A, B) Size distribution profiles of FT-ELNs$_{4T1}$ and FT-ELNs$_{B16F10}$ **(C, D)** S-ELNs$_{4T1}$ and S-ELNs$_{B16F10}$ as determined by dynamic light scattering (DLS), demonstrating a narrow monomodal size distribution.

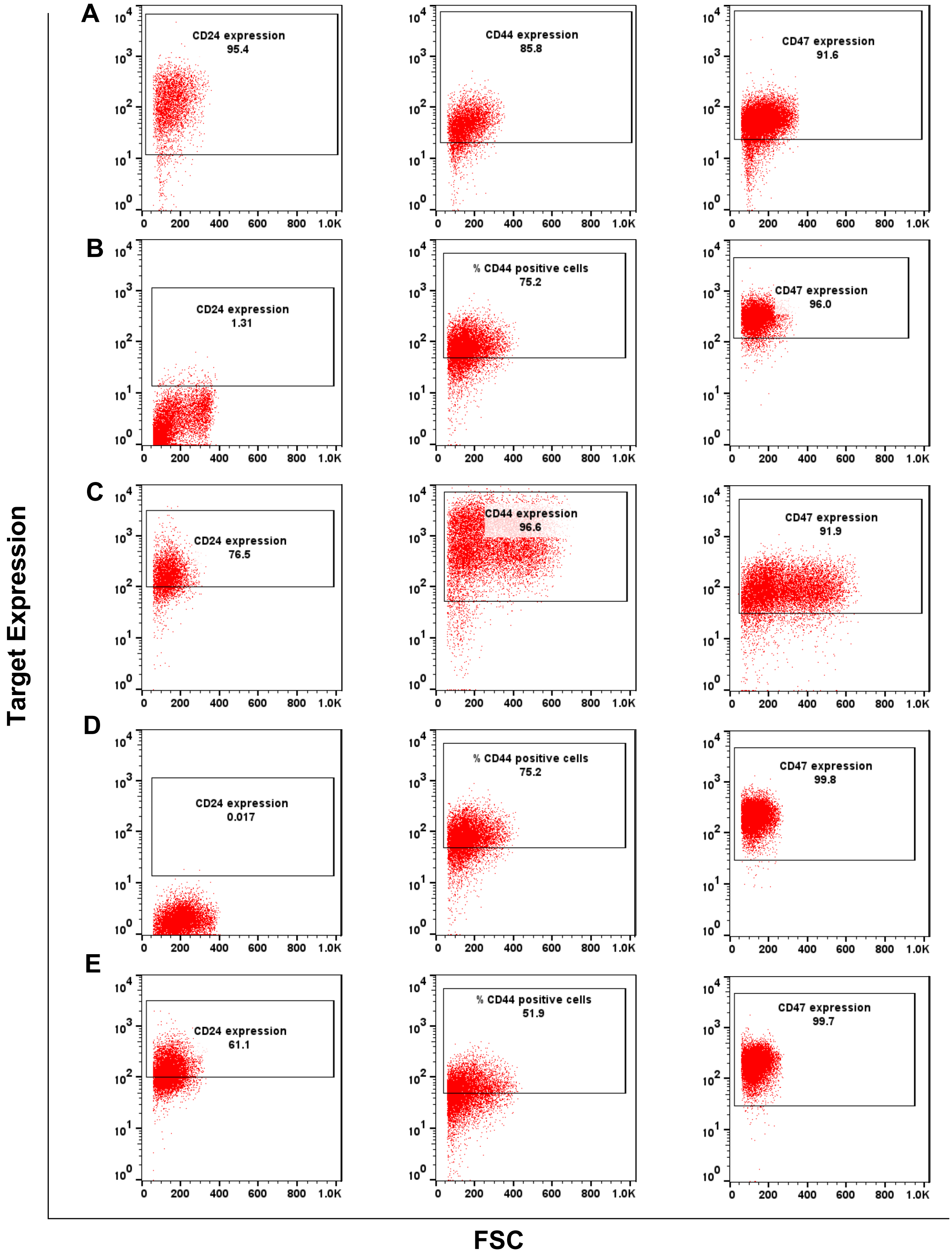

Figure S17: Target expression of different cancer cells. (A) 4T1 cells, **(B)** B16F10 cells, **(C)** BL6 cells, **(D)** CT26 cells, **(E)** GL261 cells. Different cells were stained with either anti-CD24, anti-CD44 or anti-CD47 (1: 100 v/v). Marker expression is presented as dot plot flow cytometry graphs. Gates were drawn based on isotype controls. CD24 is abundantly expressed on 4T1, BL6 and GL261 cells. Both CD44 and CD47 could be detected on all tested cell lines.

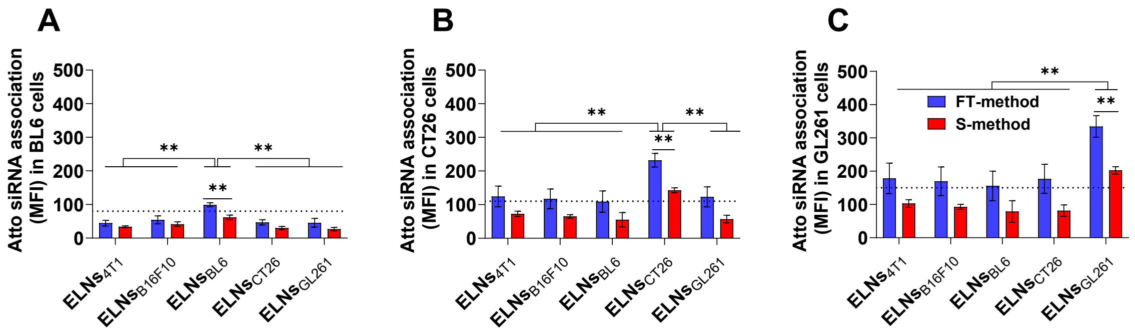

Figure S18: The optimized ELNs expressed higher siRNA association in the corresponding parent cell compared to LNP. Cells were incubated with the optimized ELNs prepared either by freeze-thaw method or sonication method loaded with Atto740 siRNA at a concentration of 30 nM for 24 h. **(A)** Cellular association of different Atto740 siRNA loaded ELNs in BL6 cells expressed as mean florescence intensity (MFI). **(B)** Cellular association of different Atto740 siRNA loaded ELNs in CT26 cells expressed as mean florescence intensity (MFI). **(C)** Cellular association of different Atto740 siRNA loaded ELNs in GL261 cells expressed as mean florescence intensity (MFI). Dashed line represents the MFI of LNP with respective values of 80.8± 10.13, 110.5± 9.17 and 150.9± 10.58 in BL6, CT26 and GL261. Cellular association of ELNs prepared by freeze-thaw method is significantly higher than that prepared sonication method ($p < 0.01$).

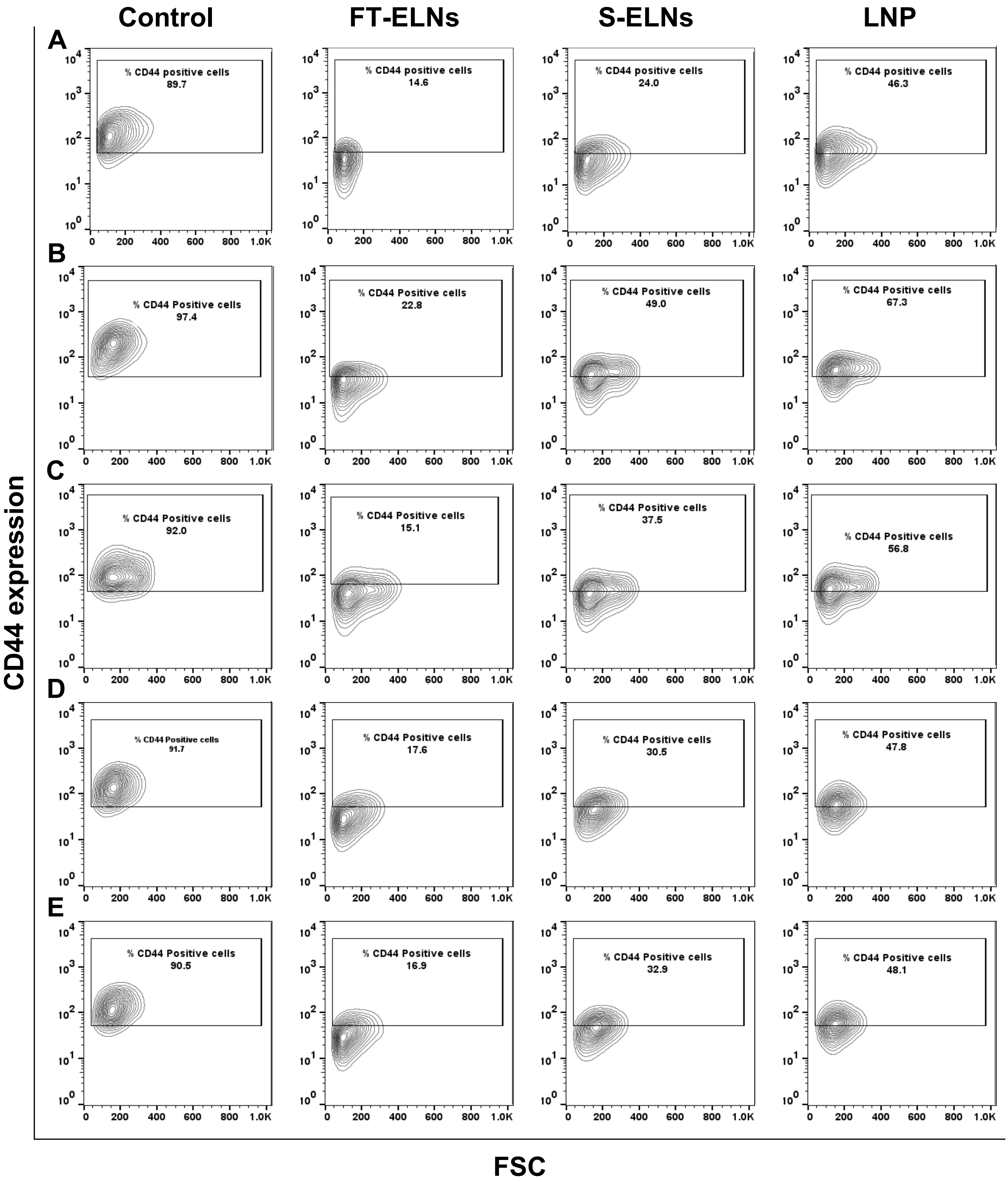

Figure S19: The optimized ELNs expressed higher gene silencing in the corresponding parent cell compared to lipid nanoparticles. (A) 4T1 cells, **(B)** B16F10 cells, **(C)** BL6 cells, **(D)** CT26 cells, **(E)** GL261 cells. Cells were incubated with either ELNs or LNP at siCD44 concentration (30 nM) for 48 h. The knock-down efficiency of CD44 is presented as percentage positive cells. Gates were drawn based on isotype controls. All ELNs had a higher CD44 silencing in its parent cells compared to LNP. The optimized ELNs prepared by freeze-thaw method showed more efficient gene silencing than that prepared by sonication method.

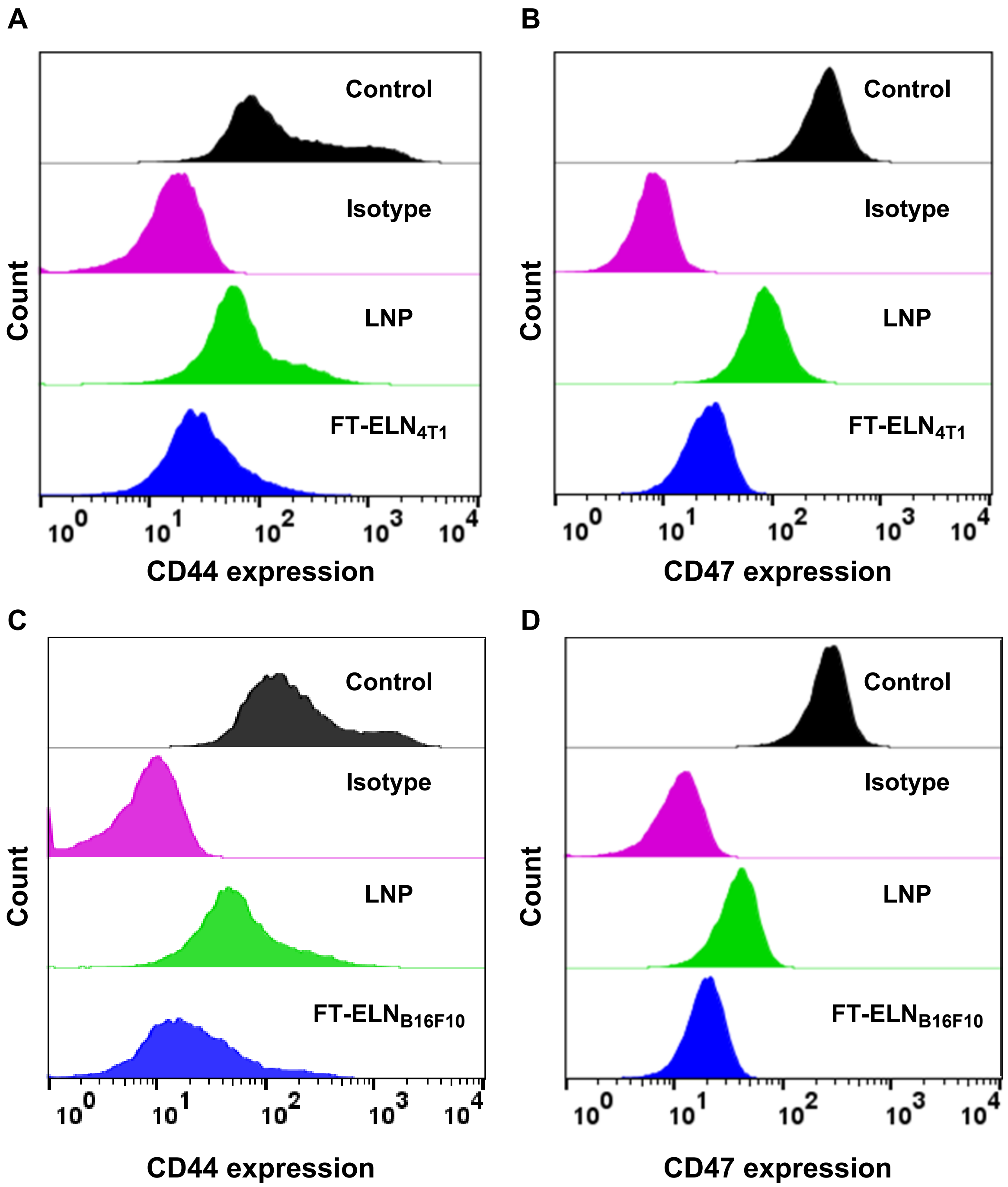

Figure S20: The optimized FT-ELNs successfully downregulate CD44 and CD47 in 4T1 and B16F10 cells. 4T1 cells were incubated with FT-ELNs$_{4T1}$ containing siCD24, siCD44 and siCD47 at concentration 10, 20 and 30 nM for 48 h. B16F10 cells were incubated with FT-ELNs$_{B16F10}$ loaded with siCD44, siCD47 at concentration 10, 20, 30 nM for 48 h. **(A)** Representative flow cytometry histograms obtained after incubating 4T1 cells with 30 nM siCD44. **(B)** Representative flow cytometry histograms obtained after incubating 4T1 cells with 30 nM siCD47. **(C)** Representative flow cytometry histograms obtained after incubating B16F10 cells with 30 nM siCD44. **(D)** Representative flow cytometry histograms obtained after incubating B16F10 cells with 30 nM siCD47.

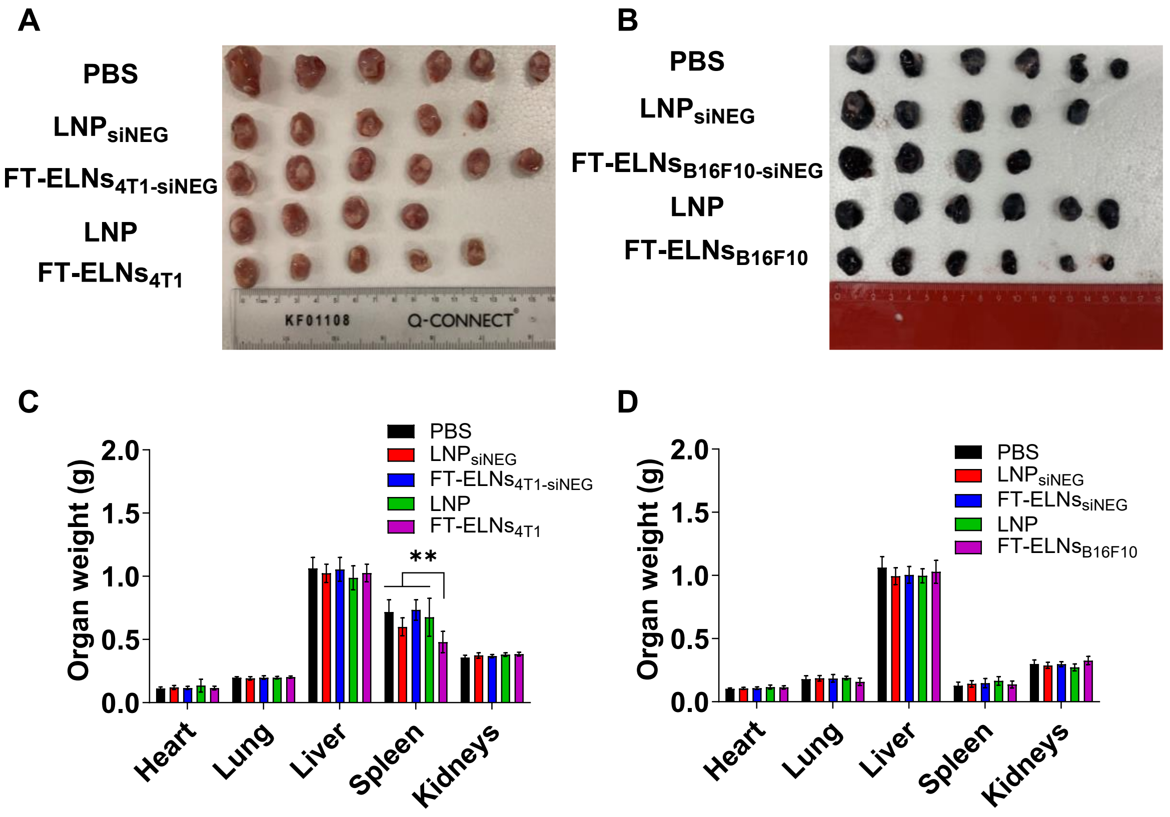

Figure S21: *In vivo* assessment of FT-ELN in 4T1 and B16F10 tumor model. BALB/c mice and C57BL/6 mice (n=6 per group) were implanted subcutaneously with 1 x 10^6 4T1 cells or B16F10 cells respectively. On day 8 and 14, BALB/c mice were i.v. injected with either PBS, LNP_{siNeg}, $FT\text{-}ELNs_{4T1\text{-}siNeg}$, LNP_s, $FT\text{-}ELNs_{4T1}$. Dose of siCD24, siCD44 and siCD47 was 33.3 µg/kg each. On day 6 and 9, C57BL/6 mice were i.v. injected with either PBS, LNP_{siNeg}, $FT\text{-}ELNs_{B16F10\text{-}siNeg}$, LNP_s, $FT\text{-}ELNs_{B16F10}$. Dose of siCD44 and siCD47 was 33.3 µg/kg each. At the terminal time point, mice were culled and tumors were imaged and weighed. (A, B) 4T1 and B16F10 tumor images at the end of the study. (C, D) Organ weights at terminal time point. Statistical analysis was performed using One way ANOVA followed by Tukey post-test, **p<0.01, data points represent the mean and SD.

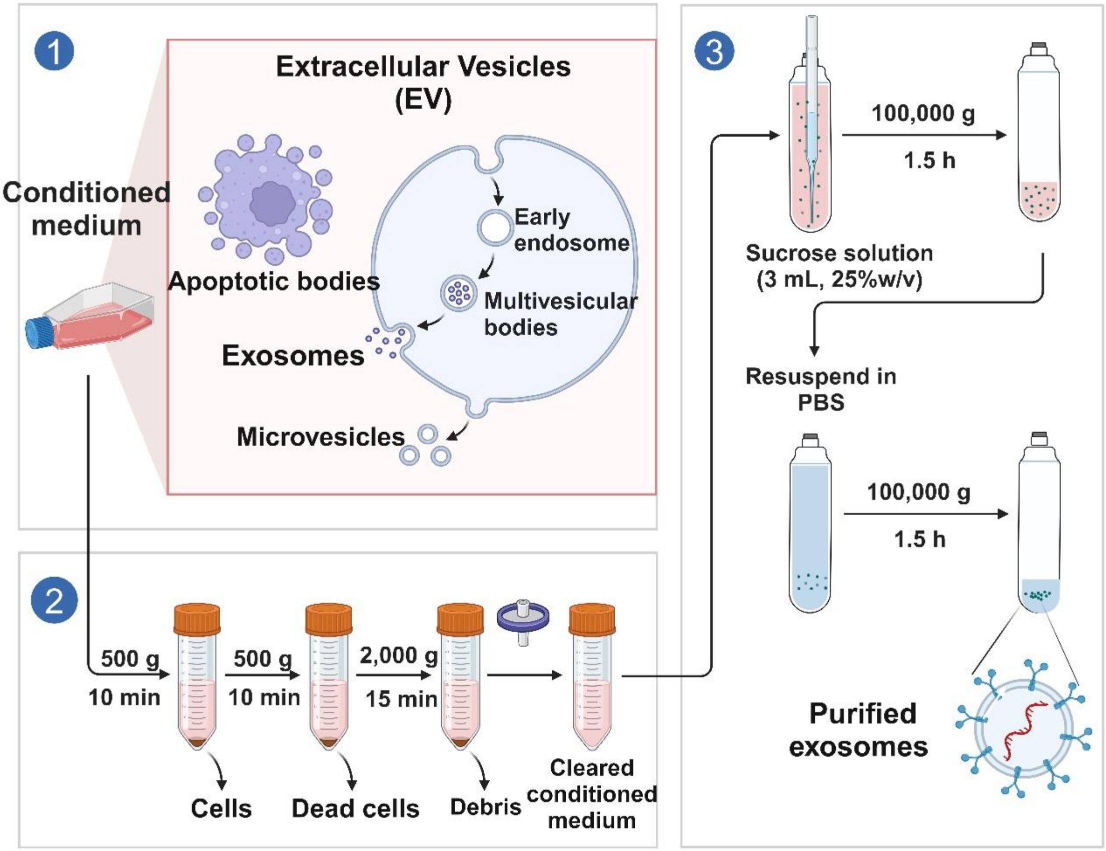

Figure S22: Exosome isolation using Ultracentrifugation onto sucrose cushion. The collected serum free culture medium from four T75 tissue culture flasks was purified from dead cells and debris by centrifugation. The exosomes were isolated from cleared conditioned medium by ultracentrifugation onto a sucrose cushion (25% w/w sucrose in D2O, density 1.18–1.20 g/mL) at 100,000 × g for 1.5 h. The collected exosome in the sucrose layer were washed by PBS and centrifuged at 100,000 × g for 90 min at 4°C. The final pellet containing exosomes was resuspended in 200 μL sterile PBS and aliquoted before storage at −80°C.

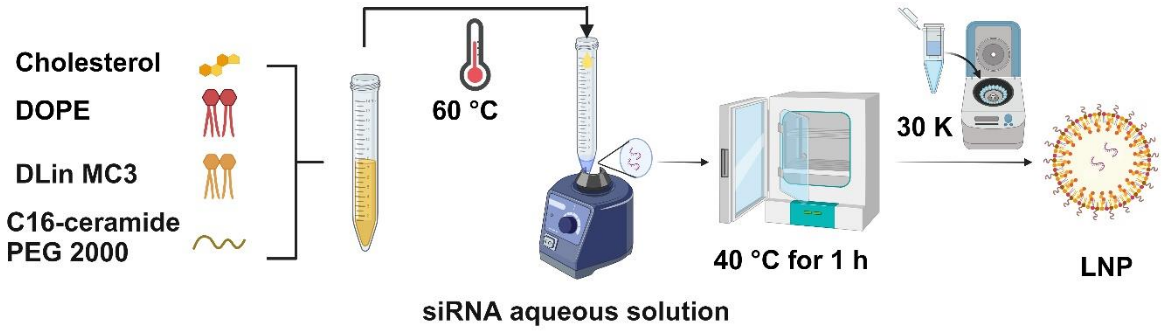

Figure S23: Preparation of lipid nanoparticles (LNP). LNP were prepared by the ethanol injection method using a lipid mixture (cholesterol, DOPE, Dlin-MC3, ceramide C16-PEG2000). siRNA was added with a Dlin-MC3 to siRNA weight ratio of 5:1. After heating both phases at 60°C for 5 min, the lipid mixture was titrated into the aqueous phase under vortex. After incubation at 40°C for 1 h, LNP buffer was exchanged with HEPES buffer.
